# Accelerating electrochemical CO_2_ reduction to multi-carbon products via asymmetric intermediate binding at confined nanointerfaces

**DOI:** 10.1038/s41467-023-36926-x

**Published:** 2023-03-09

**Authors:** Jin Zhang, Chenxi Guo, Susu Fang, Xiaotong Zhao, Le Li, Haoyang Jiang, Zhaoyang Liu, Ziqi Fan, Weigao Xu, Jianping Xiao, Miao Zhong

**Affiliations:** 1 College of Engineering and Applied Sciences, Jiangsu Key Laboratory of Artificial Functional Materials, National Laboratory of Solid State Microstructures, Nanjing University, Nanjing, 210023 China; 2 State Key Laboratory of Catalysis, Dalian National Laboratory for Clean Energy, Dalian Institute of Chemical Physics, Chinese Academy of Sciences, Zhongshan Road 457, Dalian, 116023 China; 3 Key Laboratory of Mesoscopic Chemistry, School of Chemistry and Chemical Engineering, Nanjing University, Nanjing, 210023 China; 4 University of Chinese Academy of Sciences, Beijing, 100049 China

**Keywords:** Electrocatalysis, Electrocatalysis, Electrocatalysis, Electrocatalysis

## Abstract

Electrochemical CO_2_ reduction (CO_2_R) to ethylene and ethanol enables the long-term storage of renewable electricity in valuable multi-carbon (C_2+_) chemicals. However, carbon–carbon (C–C) coupling, the rate-determining step in CO_2_R to C_2+_ conversion, has low efficiency and poor stability, especially in acid conditions. Here we find that, through alloying strategies, neighbouring binary sites enable asymmetric CO binding energies to promote CO_2_-to-C_2+_ electroreduction beyond the scaling-relation-determined activity limits on single-metal surfaces. We fabricate experimentally a series of Zn incorporated Cu catalysts that show increased asymmetric CO* binding and surface CO* coverage for fast C–C coupling and the consequent hydrogenation under electrochemical reduction conditions. Further optimization of the reaction environment at nanointerfaces suppresses hydrogen evolution and improves CO_2_ utilization under acidic conditions. We achieve, as a result, a high 31 ± 2% single-pass CO_2_-to-C_2+_ yield in a mild-acid pH 4 electrolyte with >80% single-pass CO_2_ utilization efficiency. In a single CO_2_R flow cell electrolyzer, we realize a combined performance of 91 ± 2% C_2+_ Faradaic efficiency with notable 73 ± 2% ethylene Faradaic efficiency, 31 ± 2% full-cell C_2+_ energy efficiency, and 24 ± 1% single-pass CO_2_ conversion at a commercially relevant current density of 150 mA cm^−2^ over 150 h.

## Abstract (web summary)

CO_2_ electroreduction to multi-carbon products in acids remains challenging due to the low efficiency and poor stability of C–C coupling. Here, the authors show that asymmetric CO binding at confined nanointerfaces enhances multi-carbon production, improves CO_2_ utilization, and limits H_2_ evolution.

## Introduction

Owing to rapid population and economic growth as well as increased anthropogenic activities, global energy-related CO_2_ emission reached 31.5 billion tons in 2020^1^. The resulting global warming and environmental crises require the rapid implementation of clean and efficient recycling measures to reduce the global carbon footprint.

Electrochemical CO_2_ reduction is a mild, carbon-neutral route for the large-scale transformation of waste CO_2_ into valuable chemicals using renewable electricity and water^2–7^. This approach is particularly beneficial when targeting highly demanding multicarbon (C_2+_) products such as ethylene and ethanol^8–13^. To achieve a positive net present value in techno-economic analyses of CO_2_R, > 80% selectivity and ~50% energy efficiency are required^14^. Furthermore, >15% CO_2_ conversion at a commercially relevant current density of >100 mA cm^−2^ is necessary to obtain a C_2+_ yield similar to that produced by conventional thermocatalytic CO_2_ hydrogenation^10,14^. Although much effort has been made in alkaline CO_2_R electrolysis, the relatively low CO_2_ utilization causes a great energy penalty that must be addressed before large-scale implementation. Performing CO_2_R in acidic electrolytes improves CO_2_ utilization because carbonate is difficult to form at pH ≤ 4^15^. It therefore urges the development of robust catalysts with high C–C coupling activity and potentially commercial viability.

In electrocatalytic CO_2_R systems, a fundamental issue is the slow reaction kinetics of carbon–carbon (C–C) coupling. Mechanistically, C–C coupling (C–C bond formation between CO*–CO*, CO*–CHO*, or CO*–COH* intermediates) is a (thermo-)chemical step that does not involve electron/proton transfer. Accordingly, catalyst surface polarization using external electrical energy does not significantly reduce its high reaction energy^16–18^. Considering the Brønsted–Evans–Polanyi (BEP) relations, weaker CO* adsorption is essential for lowering the C–C coupling barrier. However, according to the adsorption scaling relation, protonation to form key intermediates such as COOH*, CHO*, OCCOH*, and OCCHO* is difficult on weakly reactive catalysts. Over-weakening of the CO* binding energy also favours CO* desorption instead of C_2+_ selectivity.

Prior computational work employing volcano-type activity scaling relations predicted that stepped Cu (N11, N ≥ 2) facets show moderate CO binding energies for promoting C_2+_ production^19–21^. Indeed, experimental efforts, including facet engineering^22^, oxidation-state steering^11,17^, and grain-boundary design^23^, have realized ethylene (C_2_H_4_) selectivity of over 60% via the exposure of these Cu surfaces. Unfortunately, as reported in the literature, these stepped Cu facets are relatively unstable in the aqueous solutions^11,17^. After a few hours of CO_2_R, the stepped Cu facets are reconstructed as Cu (111) facets, which are less active for C_2+_ production, leading to substantial performance deterioration over time.

We theorize that alloying strategies, in which an enhancer metal is introduced into the low-activity but relatively stable Cu (111) facets, can create neighbouring binary sites having asymmetric surface CO binding energies to improve C–C coupling beyond the scaling-relation-determined activity limitations on single-metal surfaces. We develop an active global-energy-optimization diagram to identify the optimal route for C–C coupling on different active binary sites in electrolytes under reduction conditions. Using in situ Raman spectroscopy, electrochemical hydrodynamic simulations, and electrochemical operating experiments, we determine the key features of intermediate adsorption and electrocatalytic environment that lead to the marked CO_2_R performance with improved CO_2_ utilization in acidic conditions.

## Results

### Mechanistic studies and catalyst design

**Fig. 1:**
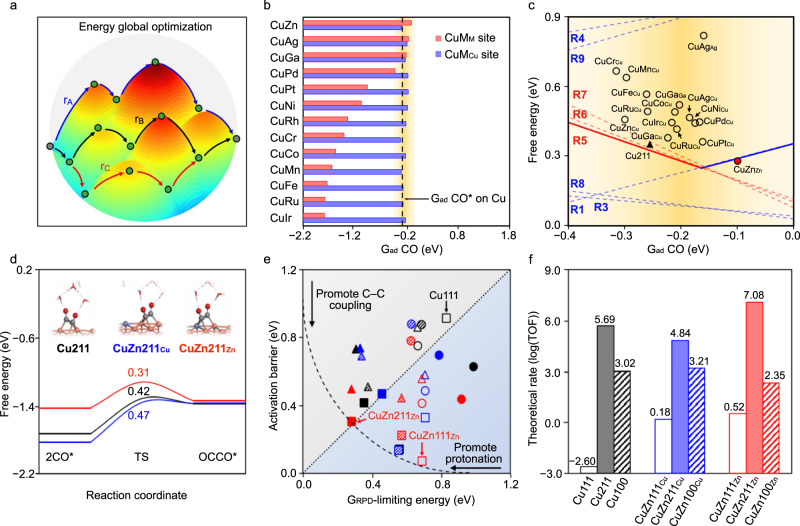
Computational studies to identify the optimal path for electrochemical C–C coupling on binary sites. **a** Energy-global-optimisation scheme. The red, blue, and black paths show different reaction channels, where r_A_, r_B_, r_C_ refer to the limiting step in relevant paths (Supplementary Fig. 5). **b** Screening of CO adsorption energies on Cu-based binary active sites. CuM_Cu_ and CuM_M_ refer to the bridge sites for the adsorbate binding between two Cu atoms, and Cu and M atoms, where M is the first neighbour atom of Cu (Supplementary Fig. 4). The yellow band shows the expected promising activities. **c** Reaction phase diagram for CO_2_R to C_2+_ at −0.6 V_RHE_. The dashed lines (red: C–C coupling steps, blue: protonation steps) indicate the reaction free energies for all considered elementary steps. The solid lines indicate the G_RPD_-limiting steps and energies. The points (triangle and circles) show the limiting energies calculated as the maximum of the reaction energy of R1 and R5. The black-filled triangle and red-filled circle refer to the reaction energy for Cu211 and CuZn_Zn_ (the most promising site). The subscript indicates the adsorption site. R1–R9 are detailed in Supplementary Table 5. **d** Activation energies for CO*–CO* coupling on Cu and CuZn (211). **e** Activation energies (black for Cu, blue for CuZn_Cu_, and red for CuZn_Zn_) plotted against the corresponding limiting potentials of the relevant paths (paths I and V for CO*–CHO*, paths II and VI for CO*–COH*, and paths III and IV for CO*–CO*; Supplementary Table 6). The squares, triangles, and circles show CO*–CO*, CO*–CHO*, and CO*–COH* coupling steps. The empty, filled, and shaded symbols correspond to the (111), (211), and (100) surfaces. **f** Reaction rates calculated using microkinetic models for CO_2_R to C_2+_ at −0.6 V_RHE_ (Methods).

To search for promising catalysts, we set out from a mechanistic analysis of CO_2_-to-C_2+_ conversion over the simplified reaction network (Supplementary Fig. 1) and calculated activity trends via density functional theory (DFT). Three major C–C coupling mechanisms were considered: CO*–CO* (R5), CO*–CHO* (R6), and CO*–COH* (R7) (Supplementary Table 5). In addition to several important protonation steps, six reaction pathways were enumerated (Supplementary Tables 5, 6). We then followed an energy-global-optimization scheme^24,25^ to determine the optimal limiting energy for a given catalyst (Fig. 1a, Methods).

To construct the activity trend, we established scaling relations, where the reaction free energies for the elementary steps were described by using the CO* adsorption energy as a descriptor on a series of catalyst surfaces (Supplementary Fig. 2). The energy-global-optimization scheme produces a volcano-like activity trend for CO_2_R to C_2+_ over single metal catalysts, described by the G_RPD_-limiting energy in the reaction phase diagram (RPD) at −0.6 V versus reversible hydrogen electrode (V_RHE_) (Supplementary Fig. 3). Our calculations reveal that the CO_2_R-to-C_2+_ activity was high on the Cu (211) surface, close to the activity volcano maximum. Our calculations also indicate that further CO*–CO* coupling could be promoted by incorporating an element that weakly binds CO* into the Cu facets to create asymmetric surface CO* bindings on the binary sites nearby. Note that over-weakened CO* binding (>−0.16 eV) on the Cu facets is undesirable^19^ because of the difficulty in forming key intermediates of COOH*, which would make protonation as the rate-limiting step for C_2+_ production with high reaction energies.

Accordingly, we used CO* adsorption energy as a descriptor to screen a series of Cu-based binary active sites (Fig. 1b). Considering a CO*-adsorption-energy window of ~0.40 eV, 16 candidate binary sites were found promising for higher CO_2_-to-C_2+_ activities (Fig. 1b). We then calculated the reaction energy in the limiting steps, namely R1 and R5 in Supplementary Table 5, for the 16 candidate sites identified from the above adsorption energy screening. The limiting energy, which is the maximum within the reaction energies of R1 and R5, was further present on the reaction phase diagram (Fig. 1c). As a result, Zn was found as an appropriate candidate for alloying with Cu, displaying a weaker CO* binding on CuZn_Zn_ site (the subscript indicates the adsorption site) than Cu to create asymmetric surface CO* binding energies with the lowest limiting energy for C–C coupling in investigated candidates.

As shown in the reaction phase diagram in Fig. 1c, CO* binding on the CuZn_Cu_ site (Supplementary Fig. 4) is relatively stronger, whereas CO* binding on the CuZn_Zn_ site is weaker (Supplementary Fig. 4). The asymmetric CO* adsorption energies on the nearby CO* adsorption sites leads to reduced reaction energy for CO*–CO* coupling than on pure Cu surfaces (Supplementary Fig. 6). Further, the G_RPD_-limiting energy on CuZn_Zn_ (R1, Supplementary Table 5) was lower than that on pure Cu (R5, Supplementary Table 5), confirming the promotion of C_2+_ activity on CuZn surfaces.

We calculated the activation barriers as critical kinetic parameters for evaluating the activity of C–C coupling. Figure 1d shows CO*–CO* coupling on Cu, CuZn_Cu_, and CuZn_Zn_ (211) facets. The kinetic energy is reduced by ~0.16 eV on the CuZn_Zn_ site. We compared the activation energies for CO*–CO*, CO*–CHO*, and CO*–COH* coupling versus the corresponding limiting potentials of the relevant paths on low-index Cu, CuZn_Cu,_ and CuZn_Zn_ (111), (211), and (100) surfaces (Fig. 1e). CuZn211_Zn_ was identified as the most active site, with a small C–C coupling barrier and a low limiting potential (Fig. 1e). Microkinetic modelling^26^ further validated the enhanced activity of the CuZn alloy for CO_2_R to C_2+_, with CuZn211_Zn_ showing the highest theoretical activity (Fig. 1f). Importantly, the most stable CuZn111_Zn_ shows significantly increased C_2+_ activity compared to Cu111, which enhanced C_2+_ production during extended CO_2_R. The HER activity was also calculated on (111), (100), and (211) surfaces of Cu and CuZn. In general, a worse HER activity was obtained on all the CuZn surfaces compared with that on Cu due to the weaker H* binding (Supplementary Fig. 7). Overall, our global energy optimization scheme indicated that alloying Zn with Cu enables asymmetric surface CO* bindings on surface binary sites to reduce the reaction energy for C–C coupling, therefore effectively promoting CO_2_-to-C_2+_ reduction.

### Model catalyst and co-sputtered catalyst studies

To verify the computational results, we fabricated a series of Cu_*x*_Zn_1−*x*_ (*x* = 0.95, 0.9, 0.85, 0.8, 0.7) model catalysts by wet etching commercial Cu_0.6_Zn_0.4_ powder (Sigma-Aldrich, Product No.: 593583-5 G, < 150 nm). The *x* values correspond to the surface Cu/Zn ratios determined by X-ray photoelectron spectroscopy (XPS) (Supplementary Fig. 8 and Table 9). The fabricated Cu_*x*_Zn_1−*x*_ model catalysts exhibited similar surface and crystal structures as that of the commercial Cu_0.6_Zn_0.4_ powder (Supplementary Fig. 9). To examine the electrochemical CO_2_R performance, we sprayed the Cu_*x*_Zn_1−*x*_ (*x* = 0.95, 0.9, 0.85, 0.8, 0.7) model catalysts and commercial Cu catalysts (Aladdin, Product No.: C103844-10G, 80–100 nm) on polytetrafluoroethylene (PTFE) substrates. Volcano-shaped curves were obtained for the C_2+_ Faradaic efficiency (FE). Notably, at 150 mA cm^−2^, the C_2+_ FE of the Cu_0.9_Zn_0.1_ model catalyst was considerably higher than that of the commercial Cu catalyst (66 ± 2% and ~50%, respectively; Supplementary Fig. 10 and Table 10). Further, for the Cu_0.9_Zn_0.1_ model catalyst, the C_2+_/C_1_ ratio of ~11 was higher than that of ~6 for the commercial Cu catalyst (Supplementary Fig. 11).

**Fig. 2:**
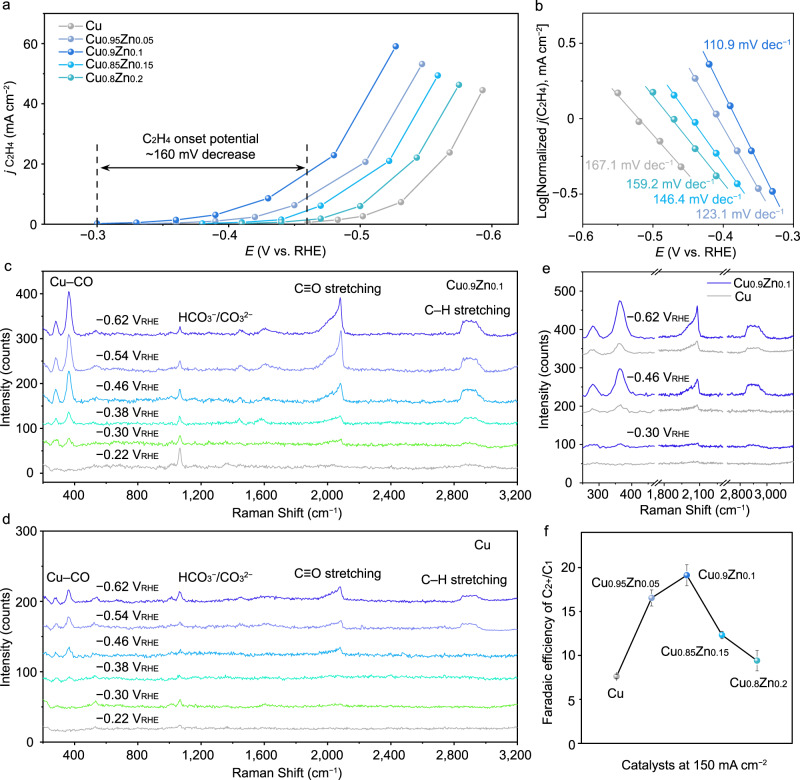
Electrochemical and in situ Raman analyses for the co-sputtered Cu_y_Zn_1−y_ (y = 0.95, 0.9, 0.85, 0.8) and Cu catalysts in 0.75 M KOH electrolyte. **a** The C_2_H_4_ partial current density near the onset potentials. **b** Tafel plots for C_2_H_4_. In situ Raman peaks for CO adsorption on **c** Cu_0.9_Zn_0.1_ and **d** Cu at various potentials during CO_2_R. **e** In situ Raman peaks at 250–470, 1750–2300 cm^−1^ and 2700–3200 cm^−1^. **f** C_2+_/C_1_ FEs. Error bars represent the standard deviation based on three independent measurements. All potentials are with respect to the reversible hydrogen electrodes (RHE).

Considering the above results, we co-sputtered Cu_*y*_Zn_1−*y*_ (*y* = 0.95, 0.9, 0.85, 0.8) catalysts on PTFE gas-diffusion electrodes. The thickness of the sputtered Cu_*y*_Zn_1−*y*_ was ~200 nm, which enabled a short distance for CO_2_ diffusion from PTFE to the Cu_*y*_Zn_1−*y*_ surface. Remarkably, the CO_2_-to-ethylene onset potential with the co-sputtered, best-performing Cu_0.9_Zn_0.1_ (−0.3 V_RHE_) was ~0.16 V smaller than that with pure Cu under the same reaction conditions (Fig. 2a). The Tafel slopes for CO_2_-to-ethylene conversion on pure Cu and Cu_0.9_Zn_0.1_ were 167.1 and 110.9 mV decade^−1^, respectively, highlighting that C–C coupling occurred faster after Zn incorporation (Fig. 2b).

We performed in situ Raman studies to explore the intermediate adsorption on the co-sputtered Cu_*y*_Zn_1−*y*_ (*y* = 0.95, 0.9, 0.85, 0.8) and Cu surfaces at different potentials in a homemade flow-cell system (Supplementary Fig. 12). As shown in Fig. 2c, d, and Supplementary Figs. 13, 14, characteristic Raman peaks were observed at ~280 and 370 cm^−1^, attributed to frustrated rotation and stretching vibrations of Cu–CO bonds^27^, at ~1900–2100 cm^−1^, attributed to stretching vibrations of C ≡ O bonds (bridge-type CO at 1900–2000 cm^−1^ and atop-type CO at 2000–2100 cm^−1^)^27,28^, and at 2800–3000 cm^−1^, attributed to C–H stretching^29^. The above Raman bands disappeared when Ar was used instead of CO_2_ under the same electrochemical conditions (Supplementary Fig. 13f), confirming that the above observed Raman peaks were representative of CO adsorption on the surfaces. Right after flowing CO_2_, quick recovery of the Cu–CO, C ≡ O, and C–H stretching peaks were observed, suggesting fast CO_2_R and C–C coupling on Cu_0.9_Zn_0.1_ (Supplementary Fig. 13g). For all the Cu and Cu_*y*_Zn_1−*y*_ surfaces, the Raman peak at ~370 cm^−1^ was slightly red-shifted as the potential became more negative, consistent with the electrochemical Stark effect^27^. The peak at ~1900–2100 cm^−1^ was also slightly red-shifted, likely due to C ≡ O dipole stretching with the increase in the surface electric field. Importantly, the Raman bands at ~280, 370, 1900–2100 cm^−1^, and 2800–3000 cm^−1^ appeared at a more positive voltage for Cu_0.9_Zn_0.1_ (−0.30 V_RHE_) than for Cu (−0.46 V_RHE_) (Fig. 2e), which is consistent with the ethylene onset potential in the electrochemical tests. The C–H stretching (2800–3000 cm^−1^) is likely attributed to intermediates containing C–H bonds such as (1) C_2_H_2_O*^29^ – the C_2+_ intermediate after C–C coupling and hydrogenation, or (2) CHO* or COH*^30^ – the key intermediates for C–C coupling via the CO*–CHO* and CO*–COH* pathways. The Raman signals of CO_3_^2−^ and HCO_3_^−^ were observed in electrolytes at pH 7 and 13.5. In clear contrast, there was no CO_3_^2−^ and HCO_3_^−^ signal in electrolytes at pH 1 and 4 during in situ Raman measurement (Supplementary Fig. 14). We observed an improved CO* peak in in situ Raman analysis with Cu_0.9_Zn_0.1_^31^. Consequently, the C_2+_/C_1_ ratio for Cu_0.9_Zn_0.1_ (~20) was ~3-fold higher than that for the Cu catalyst (~7) (Fig. 2f). Based on the above computational, in situ spectroscopic, and electrochemical operating experiments, we conclude that the increase of asymmetric CO* adsorption on the same catalyst surface is essential to promote the C–C coupling kinetics in electrolytes, considering the fact of low gas-phase CO solubility in aqueous solutions.

**Fig. 3:**
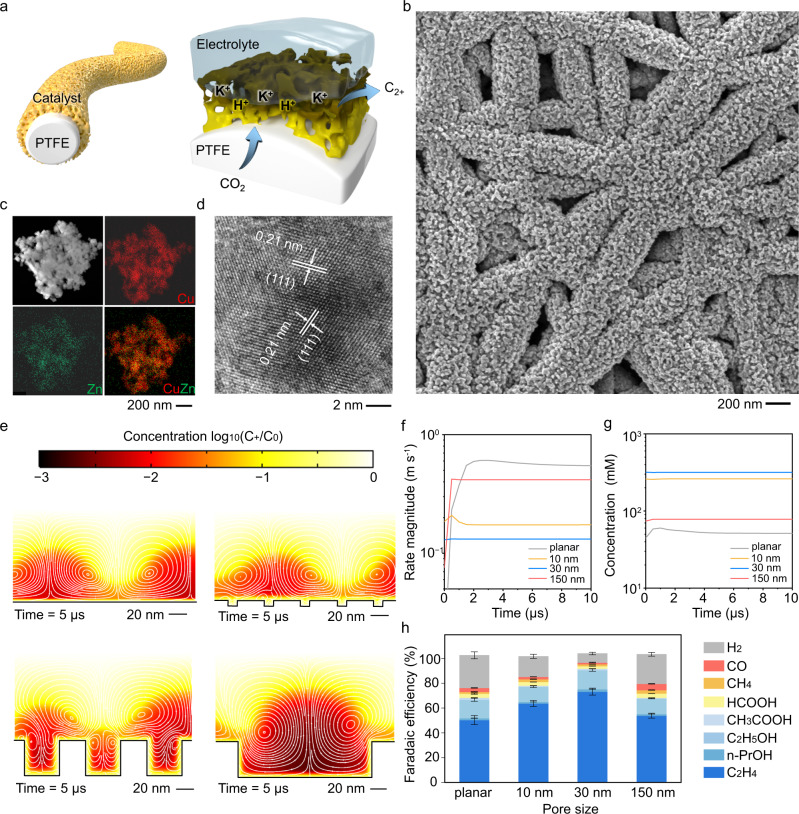
Characterisation and performance of nanoporous Cu_0.9_Zn_0.1_ catalysts fabricated by co-sputtering and wet-chemical-etching. **a** Schematic of nanoporous Cu_0.9_Zn_0.1_ for electrochemical CO_2_R. **b** SEM images of nanoporous (30 nm) Cu_0.9_Zn_0.1_. **c** STEM and EDS and **d** HRTEM images of Cu_0.9_Zn_0.1_. **e** Simulated ion concentrations and electro-kinetic flows near the planar and nanoporous (10, 30, and 150 nm) Cu_0.9_Zn_0.1_ surfaces. The white lines show the electrokinetic fluctuating vortex. **f** Magnitudes of flow rate and **g** concentrations of positive ions within the nanocavities. **h** CO_2_R performance of planar and nanoporous (10, 30, and 150 nm) Cu_0.9_Zn_0.1_ at 150 mA cm^−2^ in 0.75 M KOH electrolyte. Error bars represent the standard deviation based on three independent measurements.

To further improve the CO_2_-to-C_2+_ activity, we developed an alloying–dealloying strategy to synthesize nanoporous Cu_0.9_Zn_0.1_ catalysts on PTFE over a large area (Fig. 3a, b, and Supplementary Figs. 15, 16). The sputtered Cu forms continuous film layers on top of PTFE, different from the nanoporous structure of Cu_0.9_Zn_0.1_ after wet etching. The nanoporous structure offers a favourable local environment to concentrate ions and intermediates at the Cu_0.9_Zn_0.1_/electrolyte/CO_2_ three interfaces for improved C_2+_ conversion. Scanning transmission electron microscopy (STEM) and energy-dispersive spectroscopy analysis (EDS) images revealed that both Cu and Zn were uniformly distributed in the nanoporous Cu_0.9_Zn_0.1_ catalysts (Fig. 3c). High-resolution transmission electron microscopy (HRTEM) image gave a d-spacing of 0.21 nm for Cu_0.9_Zn_0.1_, corresponding to the Cu (111) facet (Fig. 3d). Similarly, X-ray diffraction (XRD) patterns of all the co-sputtered Cu_*y*_Zn_*1−y*_ and Cu catalysts only showed the Cu (111) diffraction peak, the position of which shifted with increasing Zn incorporation (Supplementary Fig. 17). No CuZn alloy phase was formed for the co-sputtered Cu_0.9_Zn_0.1_ before and after wet etching, suggesting that Zn was incorporated into the Cu (111) lattice which is different from the previously reported CuZn catalysts with higher Zn concentrations^32^ or in different CuZn alloy phases or phase segregated CuZn bimetallic compounds^33^. EDS in scanning electron microscopy (SEM), XPS, and inductively coupled plasma atomic emission spectroscopy (ICP-AES) analyses further confirmed a Zn concentration of ~10% for the Cu_0.9_Zn_0.1_ catalyst (Supplementary Figs. 18, 19 and Table 11). Based on the above characterization, we conclude that 10 at.% Zn incorporated Cu (111) catalysts provide abundant surface CuZn_Zn_ and CuZn_Cu_ sites with asymmetric CO binding energies improving the electrochemical C–C coupling.

To further clarify the geometrical effects at the heterogeneous nanointerfaces, we fabricated Cu_0.9_Zn_0.1_ catalysts with pore sizes of 10, 30, and 150 nm (Supplementary Figs. 20–22). Despite the increased surface area, the nanocavities concentrated ions^34^, and confined CO* intermediates to improve C_2+_ conversion^35^. Electrochemical hydrodynamic simulations in Fig. 3e showed that fast fluctuation of charges causes chaotic flows that reduce the ion concentration and intermediate retention near the catalyst surface. Compared with the planar surface or the surface with 150 nm pores, the surface with 30 nm pores generated a much lower ionic vortex velocity (Fig. 3f), which in turn increased the potassium and hydroxide ion concentrations near the catalyst surfaces to suppress hydrogen evolution and increase CO_2_R inside the nanopores (Fig. 3g)^34^. Thus, an increased local ion concentration with a prolonged duration of intermediates is expected to promote CO_2_ hydrogenation and C_2+_ selectivity (Fig. 3h).

### Electrochemical CO_2_ reduction performance

We performed CO_2_R tests for the co-sputtered and wet-chemical-etched Cu_*y*_Zn_1−*y*_ (*y* = 0.95, 0.9, 0.85, 0.8) and Cu catalysts in 0.75 M KOH electrolyte (pH 13.5). We first tested the linear sweep voltammetry (LSV) curves for Cu and Cu_*y*_Zn_1-*y*_ (*y* = 0.95, 0.9, 0.85, 0.8) catalysts in the same flow cell in Ar-saturated and CO_2_-saturated KOH electrolytes, respectively (Supplementary Fig. 23). LSV curves for all catalysts showed similar trends: the curves are flat at ~−0.3 – −0.6 V_RHE_ and show a rapid HER increase at potentials more negative than ~−0.75 V_RHE_. This ~−0.75 V_RHE_ gives ~120–180 mA cm^−2^ in CO_2_R. Since CO_2_R increases at more negative potentials, however, HER also increases at potentials more negative than −0.6 V_RHE_ and competes with CO_2_R. We thus obtained a volcano-shaped CO_2_R performance with an optimal current density of around 120–180 mA cm^−2^. The ethylene and C_2+_ FEs for nanoporous Cu_*y*_Zn_1−*y*_ were higher than those for Cu under the same current densities (100–300 mA cm;^−2^ Supplementary Figs. 24–27 and Table 12). A high C_2+_ FE of 91 ± 2% with a notable ethylene FE of 73 ± 2% was obtained for nanoporous Cu_0.9_Zn_0.1_ at 150 mA cm^−2^ and a cathodic potential of −0.55 V_RHE_ (Supplementary Fig. 24).

**Fig. 4:**
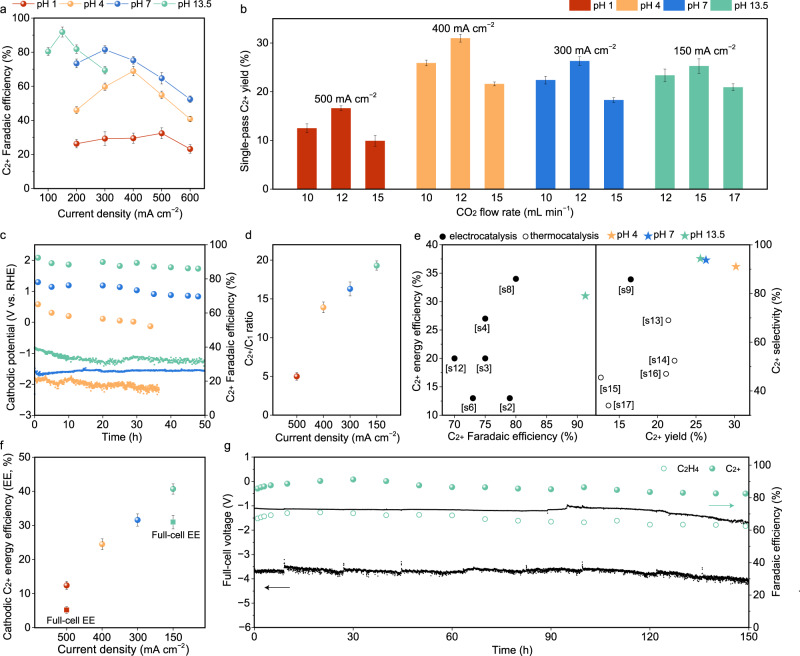
Electrochemical CO_2_R performance on Cu_0.9_Zn_0.1_ catalysts fabricated by co-sputtering and wet-chemical-etching at pH 1–13.5. **a** C_2+_ FEs at different current densities at pH 1, 4, 7, and 13.5, respectively. **b** Single-pass C_2+_ yield at different CO_2_ flow rates and current densities at pH 1, 4, 7, and 13.5 in a 13.5 cm^2^ cell. **c** CO_2_R stability curves with C_2+_ FEs at pH 4, 7, and 13.5 in a three-electrode flow cell. Current densities were optimized: 400 mA cm^−2^ at pH 4, 300 mA cm^−2^ at pH 7, and 150 mA cm^−2^ at pH 13.5, respectively (spheres: C_2+_ FEs, lines: cathodic potentials). **d** C_2+_/C_1_ ratios at different current densities at pH 1, 4, 7, and 13.5. **e** Comparison of C_2+_ FE, single-pass C_2+_ yield, and full-cell C_2+_ EE with previous reports (solid sphere: C_2+_ in electrocatalysis, hollow sphere: C_2–4_ in thermocatalysis, Supplementary Tables 20, 21). **f** C_2+_ energy efficiency (EE) at different current densities at pH 1, 4, 7, and 13.5 (sphere: cathodic C_2+_ EE, square: full-cell C_2+_ EE). **g** CO_2_R stability with C_2+_ and ethylene FEs at 150 mA cm^−2^ for Cu_0.9_Zn_0.1_ coated with graphite/carbon nanoparticles using a slim two-electrode flow-cell (The changed current density cycles consist of the work period at −150 mA cm^−2^ for 120 s (lower black line) and the regeneration period at −1 mA cm^−2^ for 30 s (upper black line)). Error bars in **a**, **b**, **d**, **f** represent the standard deviation based on three independent measurements.

We then evaluated the CO_2_R performance of Cu_0.9_Zn_0.1_ in 3 M KCl electrolytes with different pH. As reported^15^, using a concentrated, 3 M K^+^ electrolyte can efficiently decrease H^+^ concentration near the CO_2_R interface to suppress hydrogen evolution. Remarkably, Cu_0.9_Zn_0.1_ shows greatly enhanced C_2+_ performance of 69 ± 2% C_2+_ FE at 400 mA cm^−2^ at pH 4, 81 ± 2% C_2+_ FE at 300 mA cm^−2^ at pH 7, and 91 ± 2% C_2+_ FE at 150 mA cm^−2^ at pH 13.5 (Fig. 4a, Supplementary Figs. 41–47 and Tables 1–4, 13). The C–C coupling efficiency is over 95% at pH ≥4 (Supplementary Fig. 28) with a highly selective C_2+_/C_1_ ratio of ~19 at pH 13.5 (Fig. 4d). The high electrochemical CO_2_-to-C_2+_ selectivity is stable over 30 h of continuous operation at pH ≥4 (Fig. 4c, Supplementary Fig. 29). We didn’t see an obvious pH change in the catholyte and anolyte before and after the reaction (Supplementary Tables 14, 15). These results indicate that nanoporous Cu_0.9_Zn_0.1_ efficiently and stably dimerizes CO* intermediates to boost C_2+_ production.

To investigate the surface pH on electrodes during CO_2_R, we coated the Cu_0.9_Zn_0.1_ electrodes with *para*-mercaptobenzoic acid (*p*-MBA) and examined the (COO^−^)/(C = O) ratio as a function of the applied current densities. This is the same method developed by Halas and co-workers to in situ monitor the local pH near their electrode surfaces during the electrochemical reactions^36^. It is revealed that the surface pH increased linearly with the increase of current density (Supplementary Figs. 30–32), in line with the previous report^15^. (Supplementary information).

To improve the C_2+_ yield, we optimize the CO_2_ flow rate and the serpentine channel area to increase the reaction between CO_2_ and electrolyte at the interface (Supplementary Figs. 33–35 and Table 16). We achieved a single-pass C_2+_ yield of 31 ± 2% at pH 4 electrolyte at a high current density of 400 mA cm^−2^ with a CO_2_ flow rate of 12 mL min^−1^ in a 13.5 cm^2^ cell (Fig. 4b, Supplementary Table 17). This high single-pass C_2+_ yield is a 2-fold increase compared to the current best C_2+_ yield of 16.5% using fluorine modified Cu in alkaline electrolytes^10^. As reported^15^, pH ≤4 electrolytes show almost no measurable CO_2_ loss of forming carbonate. We confirmed that our single-pass CO_2_ utilization efficiency is over 80% at pH 4 under our 31 ± 2% single-pass C_2+_ yield conditions (Supplementary Tables 18, 19).

We compared the CO_2_R overpotentials and cathodic energy efficiency (EE) of C_2+_ products in the different pH electrolytes in Fig. 4f. At pH 1 and 13.5, we used a Pt plate for acidic OER and a Ni foam for alkaline OER in a silm, two-electrode CO_2_R electrolyzer, respectively. The full-cell C_2+_ EE (without *iR* compensation) reaches 31 ± 2% at pH 13.5 (Fig. 4f). This high EE is accompanied by a single-pass C_2+_ yield of 24 ± 1% at the same current density of 150 mA cm^−2^ with a C_2+_ FE of ≥90% and at a CO_2_ flow rate of 15 mL min^−1^ in a 13.5 cm^2^ cell (Supplementary Fig. 34). Significantly, we demonstrated high CO_2_R performance in a single CO_2_R system that combines: (1) a C_2+_ FE over 90% and an uncompensated full-cell C_2+_ energy efficiency over 30% for a positive net present value in techno-economic analyses of CO_2_R, and (2) C_2+_ yield over 25% at a commercially relevant current density exceeding 100 mA cm^−2^ for a C_2+_ yield similar to that produced by conventional thermal catalytic CO_2_ hydrogenation. For a broader context, we compared our data with reported electrochemical and thermal catalytic C_2+_ synthesis values (Fig. 4e, Supplementary Tables 20–22). The achieved C_2+_ yield and FE outperform the literature benchmarks, indicating critical material and system advancements in the field of CO_2_R.

Finally, we investigated the CO_2_R stability with full-cell energy efficiency in the two-electrode electrolyzer. Sargent et al.^37,38^ reported that using alternating regenerative cell potentials (−3.8 V for 60 s and −2.0 V for 30 s) improved the CO_2_R stability by reducing carbonate formation on the front and back of catalysts (Supplementary Fig. 36). Using a similar procedure, we applied alternating current density cycles of −150 mA cm^−2^ for 120 s and −1 mA cm^−2^ for 30 s. C_2+_ and ethylene were stably produced with FEs of ~90% and ~70%, respectively, at 150 mA cm^−2^ over >150 h of continuous operation (Fig. 4g). The Pourbaix diagrams of Cu and Zn indicate that cathodic protection occurs when the applied potential is more negative than the oxidation potentials of Cu and Zn (Supplementary Fig. 37). The Zn dissolution energy calculations also show that CuZn (111) surface is stable under our electrochemical conditions (Supplementary Fig. 38). In addition, we didn’t observe obvious carbonate precipitation on the front or back of the electrodes after the 150 h stability test (Supplementary Fig. 36). ICP-AES results confirmed that no obvious leaching of Cu and Zn into the electrolyte occurred during CO_2_R over 150 h (Supplementary Table 23). The full-cell voltage was stable at ~−3.7 V, enabling an uncompensated, stable full-cell CO_2_-to-C_2+_ energy efficiency of 28–32% at 150 mA cm^−2^. SEM, XRD, and XPS revealed no obvious change for the catalyst before and after the 150 h stability test (Supplementary Figs. 39, 40).

## Discussion

To sum up, this work demonstrates that the increase of asymmetric CO* adsorption and CO* surface coverage is essential for improving the electrochemical CO_2_-to-C_2+_ conversion. Further optimizing the reaction local microenvironment suppresses hydrogen evolution, thereby simultaneously improving C_2+_ selectivity and CO_2_ utilization in acidic conditions. Experimentally, we developed an alloying-dealloying strategy to fabricate homogeneously alloyed, nanoporous Cu_0.9_Zn_0.1_ catalysts that achieved a single-pass C_2+_ yield of 31 ± 2% in a mild acid pH 4 electrolyte with a single-pass CO_2_ utilization efficiency over 80%. We expect that our findings could extend the understanding of intermediates bindings and interactions on heterogeneous interfaces as these could be transformative toward practical electrochemical operation. We further note that lowering the OER overpotentials is an important future work for achieving a high full-cell energy efficiency for CO_2_R in acid or mild acid electrolytes in combination of high selectivity and high CO_2_ utilization at high CO_2_R operating current densities.

## Method

### Synthesis of CuZn model catalysts

The CuZn model catalysts were prepared by wet etching commercial Cu_0.6_Zn_0.4_ alloy powder (Sigma-Aldrich, Product No.: 593583-5 G, < 150 nm). Commercial CuZn powder (50 mg) was placed in a 30 mL Teflon inlet with 10 mL nitric acid solutions of different concentrations (0.02, 0.03, 0.05, 0.08, and 0.1 M) at 50 °C for 1 h. After wet etching, the obtained Cu_0.7_Zn_0.3_, Cu_0.8_Zn_0.2_, Cu_0.85_Zn_0.15_, Cu_0.9_Zn_0.1_, and Cu_0.95_Zn_0.05_ model catalysts were washed with deionized water and dried overnight under vacuum. Subsequently, the model catalysts were sprayed on a polytetrafluoroethylene (PTFE) substrate with the mass loading of 6 mg cm^−2^ for electrochemical CO_2_ reduction (CO_2_R) tests.

### Synthesis of nanoporous CuZn catalysts

Nanoporous CuZn catalysts with different Cu/Zn ratios were fabricated via co-sputtering and etching. First, ~200 nm CuZn thin films were co-sputtered (PD-500 magnetron sputtering coating machine) on PTFE (Beijing Zhongxingweiye Instrument Co., Ltd.) under a base pressure of 10^*−*6^ Torr (Wuhan PDVACUUM Technologies Co., Ltd.). The Cu/Zn ratios in the CuZn films were controlled by varying the Cu and Zn sputtering rates. Sputtering rates of ~2 Å s^−1^ for Cu and ~*x* Å s^−1^ (*x* = 0.2, 0.4, 0.6, 0.8) for Zn were used to produce homogeneously alloyed CuZn films. Subsequently, the as-prepared CuZn films were placed in a 30 mL Teflon inlet with 10 mL nitric acid solution (0.001 M) at 50 °C for 1 h wet etching. After wet etching, the catalysts were washed with deionized water and dried using an air gun. Cu catalysts were prepared by sputtering Cu onto PTFE. Screening of various PTFE pore sizes (0.1–0.45 μm) revealed that PTFE with 0.1 µm pores delivered the best CO_2_R performance. For the stability tests, thin layers of carbon black nanoparticles (Sigma-Aldrich, <100 nm) and graphite (Kurt J. Lesker Company) were coated on the nanoporous electrode surfaces.

### Characterization

The morphologies of the prepared samples were studied using scanning electron microscopy (SEM; Hitachi SU 5000 VPSEM) and transmission electron microscopy (TEM; FEI Talos F200X) with a field-emission gun at 200 kV. For TEM studies, the samples were scratched into an ethanol solution for dispersion. The ethanol solution was then dropped onto carbon-coated molybdenum TEM grids and dried under ambient conditions. The compositions of the samples were studied using energy-dispersive X-ray spectroscopy (EDS) system (Bruker Quantax EDS) coupled to the SEM and TEM instruments. X-ray diffraction (XRD) patterns were collected using an X’Pert-Pro MPD diffractometer (PANalytical) with a Cu Kα X-ray source (λ = 1.540598 Å). X-ray photoelectron spectroscopy (XPS) studies were conducted using an SSI S-Probe XPS spectrometer. The carbon peak (284.6 eV) was used as a reference to correct the charging effect. To investigate the bond vibrations during CO_2_R, in situ Raman spectra (200–3200 cm^−1^) were collected using a microspectrophotometer (Horiba-LabRAM HR), a long-working-distance water immersion objective (40×; U M Plan Semi Apochromat), a grating with 100 lines mm^−1^, and a homemade flow cell with a 633 nm laser as the excitation source (excitation energy of 1.96 eV). The objective lens was immersed into electrolytes in the homemade flow cell. After the sample focus, the working distance was ~2 mm from the lens to the electrode surface. All spectroscopic data were baseline corrected.

### Electrochemical reduction of CO_2_

Electrochemical CO_2_R was studied at room temperature (20–25°C) under ambient pressure using a three-electrode setup in a flow-cell reactor with an Autolab PGSTAT302N potentiostat. The prepared catalyst was used as work electrode, the work electrode reaction area was 1–4.5 cm^2^. Ag/AgCl (3.0 M KCl, Pine Instruments) was used as the reference electrode, Pt plate (Tianjin Aida Hengsheng Technology Development Co., Ltd.) and Ni foam (Shenzhen Teensky Technology Co., Ltd.) were used as the counter electrodes in pH 1, 4, 7 and 13.5 solution, respectively. The long-term stability test was carried out in a two-electrode flow cell without using an Ag/AgCl reference electrode and without *i*R correction. We used a cation exchange membrane (CEM, NafionTM 117, Fuel Cell Store) for electrochemical CO_2_ reduction in electrolytes at pH 1, 4, 7, and an anion exchange membrane (AEM, Fumasep FAB-PK-130, Fuel Cell Store) at pH 13.5. The catholyte of pH 1 were 0.05 M sulfuric acid (H_2_SO_4_) and 3 M potassium chloride (KCl) solution, and pH was adjusted to around 1 by a few drops of 5 M potassium hydroxide (KOH). The catholyte of pH 4 were 3 M KCl solution, and pH was adjusted to around 4 by a few drops of H_2_SO_4_. The catholyte of pH 7 were 3 M KCl solution, and pH was adjusted to around 7 by a few drops of 1 M KOH. The catholyte of pH 13.5 were 0.75 M KOH solution. The anolyte of pH 1 and 4 were 0.5 M H_2_SO_4_, the anolyte of pH 7 were 1 M KHCO_3_, and the anolyte of pH 13.5 were 0.75 M KOH. Electrochemical impedance spectroscopy (EIS) was measured to estimate the electrolyte resistance for *i*R compensation. The electrolyte resistance was measured at open circuit potential in a frequency range from 10 MHz to 0.1 Hz with an amplitude of 10 mV. All potentials versus the reference electrodes were converted to potentials versus the reversible hydrogen electrode (V_RHE_) using the following equations:

1 $${V}_{{RHE}}={V}_{{Ag}/{AgCl}}+0.059\times {{{{{\rm{pH}}}}}}+{E}_{{AgCl}}^{0},$$

2 $${E}_{{AgCl}}^{0}\left(3.0{{{{{\rm{M\; KCl}}}}}}\right)=0.197\,{{{{{\rm{V}}}}}}(25\,^\circ {{{{{\rm{C}}}}}}),$$

CO_2_ was passed through the cathodic compartment at a constant flow rate of 20 mL min^−1^. The gaseous CO_2_ reduction products were quantified using gas chromatography (Perkin Elmer Clarus 680). The liquid products were quantified using NMR spectroscopy (Bruker AVIII 600 MHz) and dimethyl sulfoxide (≥99.9%, Alfa Aesar) was added as an internal standard. The ^1^H NMR spectrum was measured by water suppression using the pre-saturation method. All experimental results were repeated at least three times while keeping all conditions consistent.

### Calculation of CO_2_R performance

The product FE in CO_2_R were all obtained with bare catalysts without surface coating. Once we identify the best-performing catalyst, we coated the catalyst with a carbon layer for the long-term stability test. It showed the same performance as that without the carbon layer coating. The coating of the carbon layer only made the electrical field more homogeneous during long-term operation which was reported in the previous study^2^.

The Faradaic efficiencies (FEs) of the gas and liquid products were calculated using the following equations:

3 $${{FE}}_{{gas}}(\%)=\alpha \times n\times F/Q=\alpha \times {C}_{{gas}}\times {f}_{{CO}2}\times t\times F/(22.4\times Q)\times 100\%,$$

4 $${{FE}}_{{liquid}}(\%)=\alpha \times n\times F/Q=\alpha \times V\times \rho \times F/(M\times Q)\times 100\%,$$

where *f*_*CO2*_ is the CO_2_ flow rate, *C*_*gas*_ is the gas concentration, *α* is the number of electrons transferred for each product, *t* is the reaction time, ρ is the density of the liquid products, *M* is the relative molecular mass of the liquid products, *V* is calculated using the standard ^1^H NMR curve, and *Q* (*A*·*S*) is the total electric charge.

The half-cell C_2+_ energy efficiency was calculated as follows:

5 $${{EE}}_{{cathodic}{half}-{cell}}=	(1.23-{E}_{{ethylene}})\times {{FE}}_{{ethylene}}/(1.23-{E}_{{applied}}) \\ 	+(1.23-{E}_{{ethanol}})\times {{FE}}_{{ethanol}}/(1.23-{E}_{{applied}}),$$

The full-cell C_2+_ energy efficiency was calculated as follows:

6 $${{EE}}_{{full}-{cell}}=	(1.23-{E}_{{ethylene}})\times {{FE}}_{{ethylene}}/{E}_{{applied}}+(1.23-{E}_{{ethanol}})\\ 	 \times {{FE}}_{{ethanol}}/{E}_{{applied}},$$

where *E*_*applied*_ is the potential during full-cell CO_2_R, *E*_*ethylene*_ is the thermodynamic potential (vs. RHE) of ethylene for CO_2_R, which is 0.06 V for ethylene, and *E*_*ethanol*_ is the thermodynamic potential (vs. RHE) of ethanol for CO_2_R, which is 0.09 V for ethanol^39^.

The C_2+_ selectivity was calculated as (the rate of C_2+_ product formation (R_product_))/(the rate of total product formation (R_tot_)), as follows:

7 $${{{{{{\rm{C}}}}}}}_{2+}\,{{{{{\rm{selectivity}}}}}}=	(2{{{{{{\rm{R}}}}}}}_{{{{{{\rm{ethylene}}}}}}}+2{{{{{{\rm{R}}}}}}}_{{{{{{\rm{acetate}}}}}}}+2{{{{{{\rm{R}}}}}}}_{{{{{{\rm{ethanol}}}}}}}+3{{{{{{\rm{R}}}}}}}_{{{{{{\rm{n}}}}}}-{{{{{\rm{propanol}}}}}}})/ \\ 	({{{{{{\rm{R}}}}}}}_{{{{{{\rm{CO}}}}}}}+{{{{{{\rm{R}}}}}}}_{{{{{{\rm{formate}}}}}}}+{{{{{{\rm{R}}}}}}}_{{{{{{\rm{methane}}}}}}}+2{{{{{{\rm{R}}}}}}}_{{{{{{\rm{ethylene}}}}}}}+2{{{{{{\rm{R}}}}}}}_{{{{{{\rm{acetate}}}}}}} \\ 	+2{{{{{{\rm{R}}}}}}}_{{{{{{\rm{ethanol}}}}}}}+3{{{{{{\rm{R}}}}}}}_{{{{{{\rm{n}}}}}}-{{{{{\rm{propanol}}}}}}})\times 100\%,$$

The single-pass C_2+_ yield was calculated as (the rate of C_2+_ product formation (R_product_))/(the flow rate of CO_2_ (*f*_CO2_)), as follows:

8 $${{{{{{\rm{C}}}}}}}_{2+}\,{{{{{\rm{yield}}}}}}=(2{{{{{{\rm{R}}}}}}}_{{{{{{\rm{ethylene}}}}}}}+2{{{{{{\rm{R}}}}}}}_{{{{{{\rm{acetate}}}}}}}+2{{{{{{\rm{R}}}}}}}_{{{{{{\rm{ethanol}}}}}}}+3{{{{{{\rm{R}}}}}}}_{{{{{{\rm{n}}}}}}-{{{{{\rm{propanol}}}}}}})/{{fCO}}_{2}\times 100\%,$$

where R is the rate of C_2+_ product formation, as determined by gas chromatography analysis of the product.

### DFT calculations

The Vienna ab initio simulation package (VASP)^40^ was used for density functional theory (DFT) calculations. The revised Perdew–Burke–Ernzerhof (rPBE) functional^41^ was used with the basic projector-augmented wave (PAW) method with a cut-off energy of 400 eV. The bulk structures (Cu, Rh, Pt, and Ag) were optimized using a Monkhorst–Pack k-point of 4 × 4 × 4, the (211) surfaces were built first to build the activity trend for CO_2_R, where, in general, metal (211) surfaces were reported more active for catalytic C–C coupling compared to other low-index metal surfaces^7^. Hereafter, as the most promising metal performing higher activity, and the (111), (211), and (100) surfaces for Cu were built with four layers comprising 64, 48, and 64 atoms, respectively. All the surface structures were calculated with half of the atoms fixed from the bottom, and the rest of the atoms were relaxed. The CuZn alloy was then obtained from the screening as the most promising alloy performing higher activity for CO_2_R, which was built based on a Cu:Zn ratio of 0.9:0.1 (Cu_0.9_Zn_0.1_). Accordingly, 7, 5, and 7 Cu atoms were replaced by Zn on the Cu(111), (211), and (100) surfaces, respectively (Supplementary Fig. 2). Monkhorst–Pack k-points of 2 × 2 × 1, 3 × 2 × 1, and 2 × 2× 1 were used for the (111), (211), and (100) surfaces, respectively. The transition states were located using the method of constrained optimisation which has been widely used in many previous works^18,24,25^, with a force convergency of 0.05 eV Å^−1^ for both structural optimization and transition state calculations. No protonation barriers were considered in the microkinetic modelling.

### Free energy corrections

All calculated energies, including adsorption and gas energies, were corrected to the free energy at 298 K, as follows:

9 $${{{{{{\rm{E}}}}}}}_{{{{{{\rm{cor}}}}}}}={{{{{{\rm{E}}}}}}}_{{{{{{\rm{cor}}}}}}}^{{{{{{\rm{ZPE}}}}}}}+{{{{{{\rm{E}}}}}}}_{{{{{{\rm{cor}}}}}}}^{{{{{{\rm{U}}}}}}}+{{{{{{\rm{E}}}}}}}_{{{{{{\rm{cor}}}}}}}^{{{{{{\rm{P}}}}}}}+{{{{{{\rm{E}}}}}}}_{{{{{{\rm{cor}}}}}}}^{{{{{{\rm{S}}}}}}},$$

where ${{{{{{\rm{E}}}}}}}_{{{{{{\rm{cor}}}}}}}^{{{{{{\rm{ZPE}}}}}}}$ is the zero-point energy correction, ${{{{{{\rm{E}}}}}}}_{{{{{{\rm{cor}}}}}}}^{{{{{{\rm{U}}}}}}}$ and ${{{{{{\rm{E}}}}}}}_{{{{{{\rm{cor}}}}}}}^{{{{{{\rm{P}}}}}}}$ are the temperature (inner energy) and pressure corrections, respectively, and ${{{{{{\rm{E}}}}}}}_{{{{{{\rm{cor}}}}}}}^{{{{{{\rm{S}}}}}}}$ is the entropy correction (Supplementary Table 7). At equilibrium, the chemical potential of a pair of (H^+^ + e^−^) ions at 0 V_RHE_ is referred to as the half chemical potential of H_2_ molecules considering the computational hydrogen electrode (CHE) approximation^42^. Therefore, the chemical potential of OH^−^ was obtained as:

10 $${\triangle {{\mbox{G}}}}_{{{{\mbox{OH}}}}^{-}}={{{\mbox{G}}}}_{{{{\mbox{H}}}}_{2}{{\mbox{O}}}}-{{{\mbox{G}}}}_{{{{\mbox{H}}}}^{+}},$$

The potential-dependent reaction free energy was calculated using the CHE approximation:

11 $${\triangle {{\mbox{G}}}}_{{{\mbox{U}}}}={{{\mbox{G}}}}_{{{{\mbox{U}}}}_{0}}={{{{{\rm{e}}}}}}\left({{\mbox{U}}}-{{{\mbox{U}}}}_{0}\right),$$

where U and U_0_ are electrode potentials (U and 0 V_RHE_, respectively).

### Solvent effect corrections

Adsorption stabilization has been reported to mainly originate from the formation of H-bonds, namely, the solvent effect. In this study, solvation effects were simulated based on an implicit model, VASP_sol_ (Supplementary Table 8). The solvation effects were also examined using explicit models, which validated the results from the implicit model (Supplementary Table 8). Note that the solvent effect for OCCO* was simulated using polarised explicit water models, for which the uncertainty of the adsorption energy has been reported to be negligible^20^.

### Energy global optimization

By considering the elementary steps in the simplified reaction network, all possible pathways can be enumerated^24,25^. The elementary step with the highest limiting energy was defined as the limiting step in a specific pathway (*r*_A_, *r*_B_, and *r*_C_ in Supplementary Fig. 3). The pathway with the lowest limiting energy among all possible pathways was chosen as the most favoured pathway (red path in Supplementary Fig. 3), with the relevant limiting energy defined as the G_RPD_-limiting energy (G_RPD_), as follows:

12 $${{{\mbox{G}}}}_{{{\mbox{RPD}}}}={\min }_{{{\mbox{i}}}}\left[{\max }_{{{\mbox{j}}}}\left({{{\mbox{G}}}}_{{{\mbox{i}}},{{\mbox{j}}}}\right)\right],$$

where ${{{\mbox{G}}}}_{{{\mbox{i}}},{{\mbox{j}}}}$ refers to the reaction energy for elementary step *j* in pathway *i*. As a result, the reaction pathway with globally optimal limiting energy can be determined (red path, Supplementary Fig. 3) rather than an empirical step-by-step comparison from reactants to products (blue and black paths, Supplementary Fig. 3).

### Rate simulation

Microkinetic modelling was used to simulate the reaction rate of CO_2_R-to-C_2+_ on Cu and CuZn surfaces, where the temperature was set to 298 K. The kinetic constant for all elementary steps (Supplementary Table 5) was obtained based on the Arrhenius equation, as follows:

13 $${{{{{\rm{k}}}}}}={{{{{\rm{A}}}}}}\times {{{{{{\rm{e}}}}}}}^{-\frac{{{{\mbox{E}}}}_{{{\mbox{a}}}}}{{{\mbox{RT}}}}},$$

where *A* refers to the pre-factor, *Ea* refers to the activation barrier, *R* refers to the molar gas constant, and *T* refers to the temperature. The steady state was located at d*θ*/d*t* = 0, and the reaction rate was obtained, described by the production rate of C_2_H_4_.

### COMSOL simulations

We used a commercial software *COMSOL* to perform the simulation:^43^ The potential and flow fields were governed by the Poisson equation, Stokes equations, and the continuity equations as follows:

14 $$\varepsilon {\nabla }^{2}\psi=\mathop{\sum }\limits_{i}F{z}_{i}{c}_{i},$$

15 $$\rho \frac{\partial u}{\partial t}=-\nabla p+\mu {\nabla }^{2}u-\mathop{\sum }\limits_{i}F{z}_{i}{c}_{i}\nabla \psi,$$

16 $$\nabla u=0,$$

17 $$\frac{\partial {c}_{i}}{\partial t}=-\nabla \left(-{D}_{i}\nabla {c}_{i}-\frac{{z}_{i}F{D}_{i}}{{RT}}{c}_{i}\nabla \psi+{c}_{i}u\right),$$

Where *ℇ* is the electrical permittivity, *Ψ* is the electric potential, *F* is the Faraday constant, *z*_*i*_ is the valence of species i, *c*_*i*_ is the ion concentration of species i, *ρ* is the density, *u* is the flow field, *t* is the time, *p* is the pressure, and *μ* is the dynamic viscosity. The mass transfer processes of ion were described by the Nernst Planck equation, where *D*_*i*_ is the diffusivity of species i, *R* is the gas constant, and *T* is the temperature.

Similar to the previous report^34^, we set the parameters for simulation as follows: we set the diffusion coefficient of 1.957 × 10^−9^ m^2^/s for K^+^ and the applied surface voltage of −1.7 V on the surface for performing the simulations using the above governing equations.

Importantly, in the electro-chaotic systems, the vertex chaos or ion convection is mainly determined by the electrostatic forces and not by the fluid inertia^34,43^. The ion concentrations near surfaces are mainly governed by the applied voltages. After a few microseconds, the concentrations of ions in the 30 nm pores reach a quasi-steady state, an order of magnitude higher than that on the flat surface or in the 150 nm pores. The numerical simulations showed that 30 nm pores increased the potassium concentrations near the surfaces. As a result, hydrogen evolution reaction (HER) is suppressed.

## Supplementary information

Supplementary Information

## Data Availability

Source data to generate figures and tables are available from the corresponding authors.
