# Supplementary material for: Accelerating electrochemical CO2 reduction to multi-carbon products via asymmetric intermediate binding at confined nanointerfaces
Source: Nat Commun. 2023 Mar 9;14:1298. doi: 10.1038/s41467-023-36926-x (PMC9998885; doi:10.1038/s41467-023-36926-x)
Supplement: Supplementary file 1 — Supplementary Information [file 41467_2023_36926_MOESM1_ESM.pdf]

Supplementary Information

Accelerating electrochemical CO_2 reduction to multi-carbon products via asymmetric intermediate binding at confined nanointerfaces

Jin Zhang[†,1], Chenxi Guo[†,2], Susu Fang[3], Xiaotong Zhao[1], Le Li[1], Haoyang Jiang[1], Zhaoyang Liu[1], Ziqi Fan[1], Weigao Xu[3], Jianping Xiao[*,2,4], Miao Zhong[*,1]

[1]College of Engineering and Applied Sciences, Jiangsu Key Laboratory of Artificial Functional Materials, National Laboratory of Solid State Microstructures, Collaborative Innovation Center of Advanced Microstructure, Nanjing University, Nanjing 210023, China.

[2]State Key Laboratory of Catalysis, Dalian National Laboratory for Clean Energy, Dalian Institute of Chemical Physics, Chinese Academy of Sciences, Zhongshan Road 457, Dalian 116023, China.

[3]Key Laboratory of Mesoscopic Chemistry, School of Chemistry and Chemical Engineering, Nanjing University, Nanjing 210023, China.

[4]University of Chinese Academy of Sciences, Beijing 100049, China.

[†] These authors contributed equally to this work.

[(*)] Correspondence and requests for materials should be addressed to Miao Zhong (miaozhong@nju.edu.cn) and Jianping Xiao (xiao@dicp.ac.cn)

Supplementary Figures

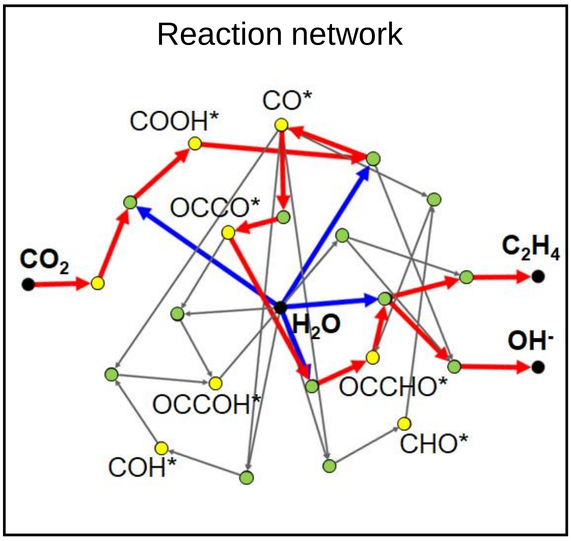

Supplementary Fig. 1 | Simplified reaction network for CO_2R to C_{2+} with three C–C coupling pathways: CO*–CO*, CO*–CHO*, and CO*–COH*. The black, green, and yellow points refer to the reactant/product, intermediate states, and transition states, respectively. The red and blue arrows show an energetically favourable pathway for C_{2+} production. The mathematical algorithm for the construction of the reaction network can be found in Method.

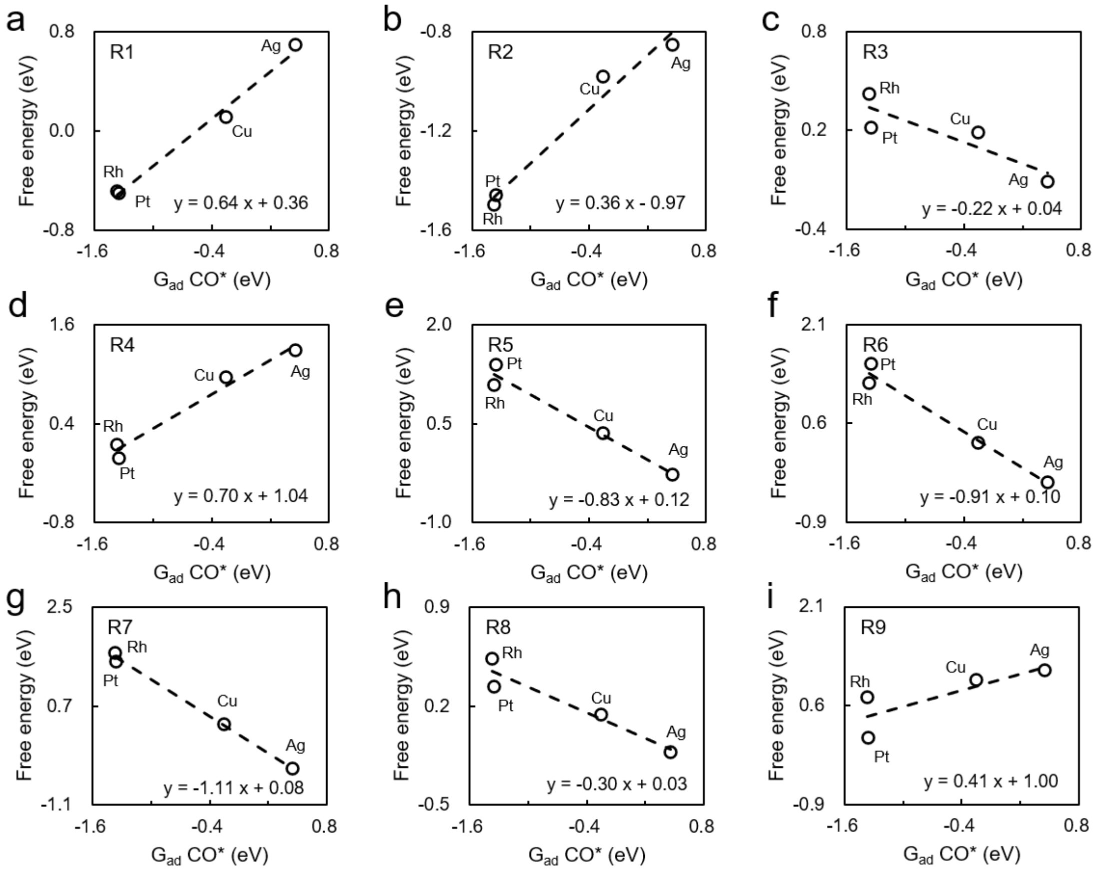

Supplementary Fig. 2 | Scaling relations between the reaction free energies of (a–i) R1–R9 (Supplementary Table 5) and the CO* adsorption energies on Pt, Rh, Cu, and Ag.

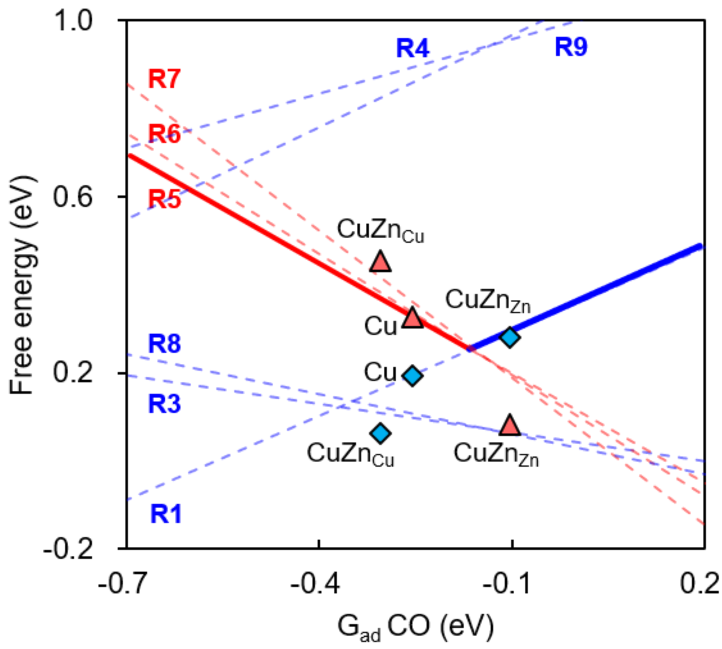

Supplementary Fig. 3 | Reaction phase diagram for CO_2R to C_{2+} at −0.6 V_{RHE}. The dashed lines (red: C–C coupling steps, blue: protonation steps) indicate the reaction free energies for all considered elementary steps. The solid lines indicate the G_{RPD}-limiting steps and energies. The triangles (R5) and diamonds (R1) show the calculated energies on Cu and CuZn alloys (the subscript indicates the adsorption site). R1–R9 are detailed in Supplementary Table 5.

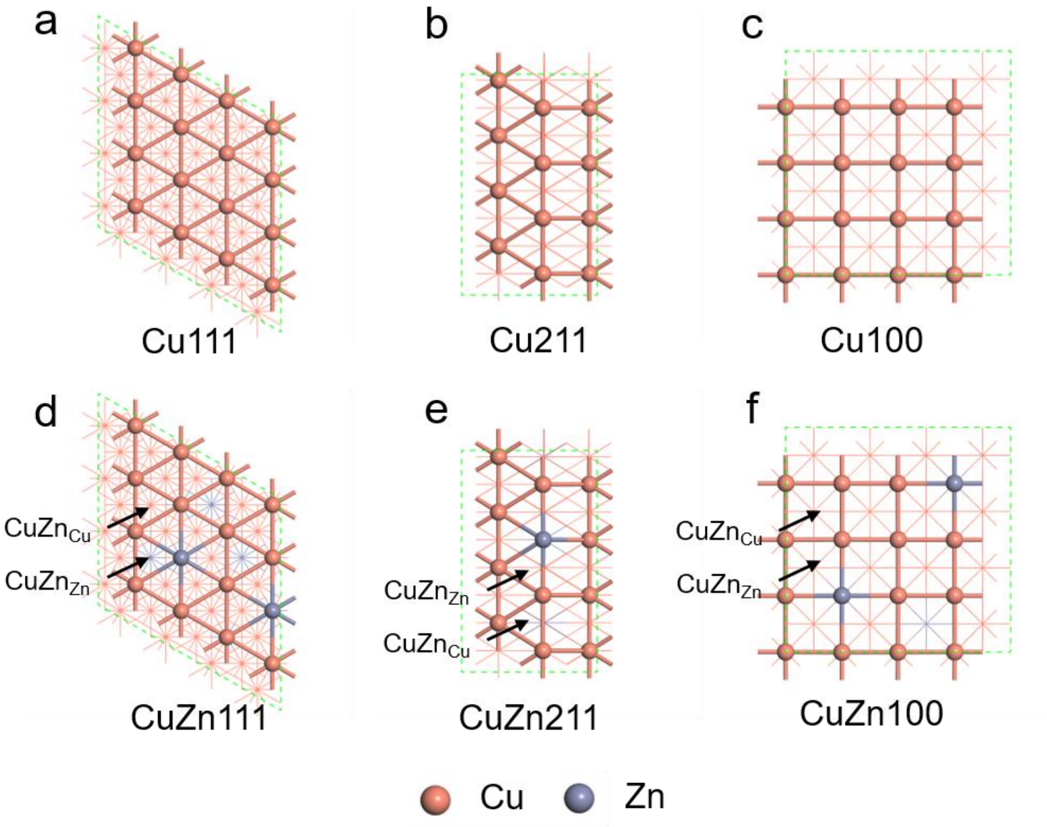

Supplementary Fig. 4 | Surface structures of (a) Cu111, (b) Cu211, (c) Cu100, (d) CuZn111, (e) CuZn211, and (f) CuZn100. $CuZn_{Cu}$ and $CuZn_{Zn}$ refer to Cu and CuZn active sizes, respectively.

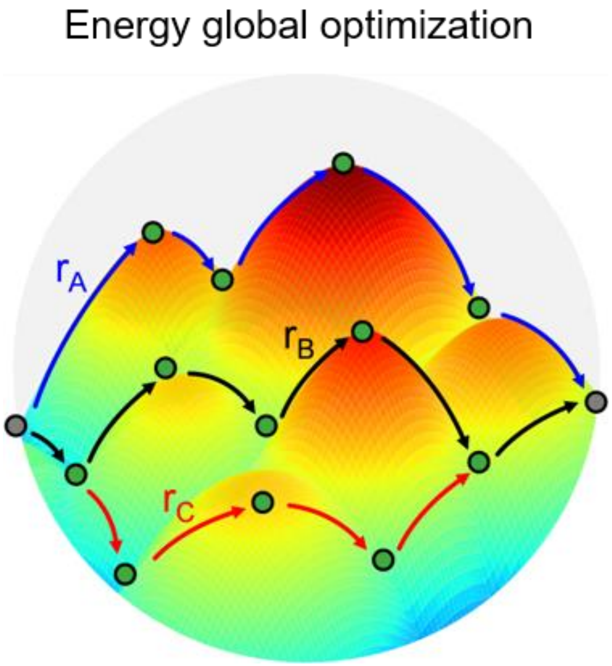

Supplementary Fig. 5 | Energy-global-optimisation scheme. The red, blue, and black paths represent different reaction channels, where r_A, r_B, r_C refer to the reaction rates of the corresponding limiting steps in the relevant paths. The red path was determined as the favored energy-global pathway.

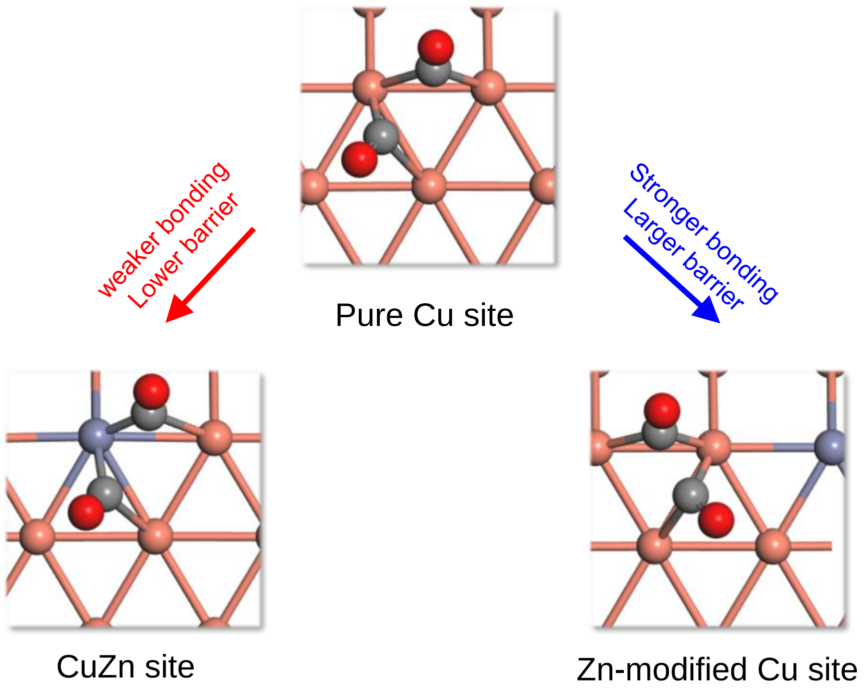

Supplementary Fig. 6 | CO*–CO* coupling on the alloyed CuZn211 and Cu211 surface. The $CuZn_{Zn}$ bridge site shows a weaker CO* binding, which results in a lower CO*–CO* coupling barrier, while a stronger binding ability was obtained for the $CuZn_{Cu}$ site with a higher coupling barrier for CO*–CO*.

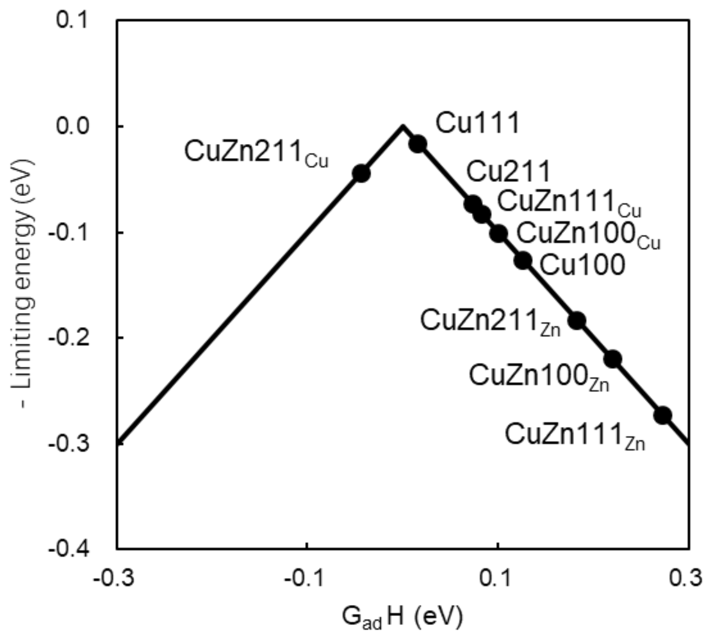

Supplementary Fig. 7 | HER activity on (111), (211), and (100) surfaces of Cu and CuZn.

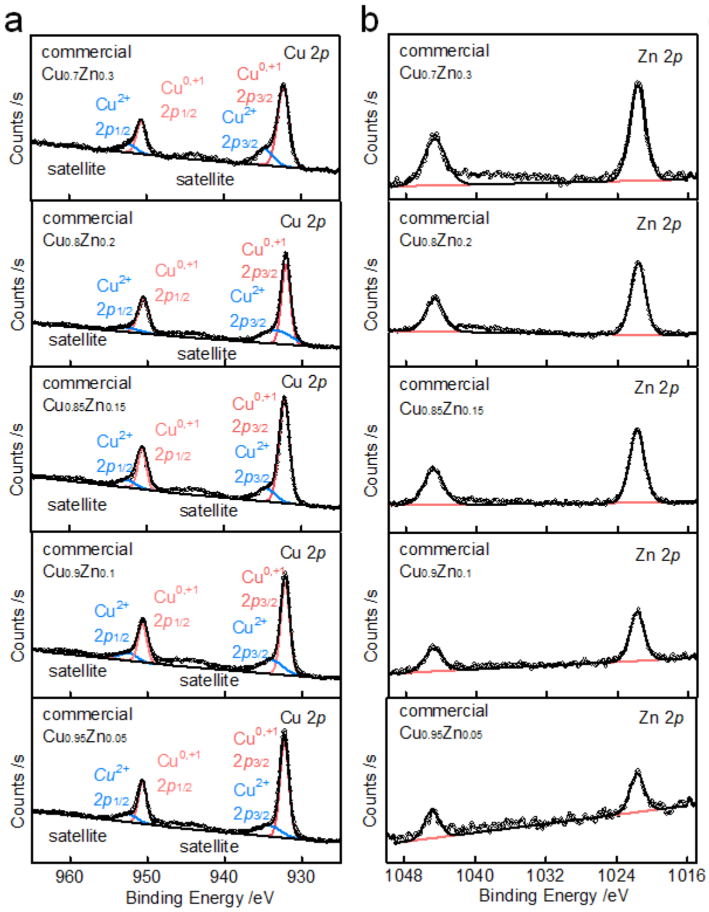

c

Atomic Concentration Table		
Element	Wt /%	rel. error /% 1 sigma
Cu	70.2	0.2
Zn	29.8	0.2
Total:	100.0	

Atomic Concentration Table		
Element	Wt /%	rel. error /% 1 sigma
Cu	79.8	0.3
Zn	20.2	0.3
Total:	100.0	

Atomic Concentration Table		
Element	Wt /%	rel. error /% 1 sigma
Cu	85.3	0.3
Zn	14.7	0.3
Total:	100.0	

Atomic Concentration Table		
Element	Wt /%	rel. error /% 1 sigma
Cu	89.5	0.2
Zn	10.5	0.2
Total:	100.0	

Atomic Concentration Table		
Element	Wt /%	rel. error /% 1 sigma
Cu	94.3	0.2
Zn	5.7	0.2
Total:	100.0	

Supplementary Fig. 8 | XPS analysis of wet-etched Cu_xZn_{1-x} (x = 0.95, 0.9, 0.85, 0.8, 0.7) model catalysts fabricated by wet chemical etching of the commercial $Cu_{0.6}Zn_{0.4}$ powder (Sigma-Aldrich, Product No.: 593583-5G, <150 nm): (a) Cu 2*p* spectra, (b) Zn 2*p* spectra, and (c) ratios of Cu and Zn.

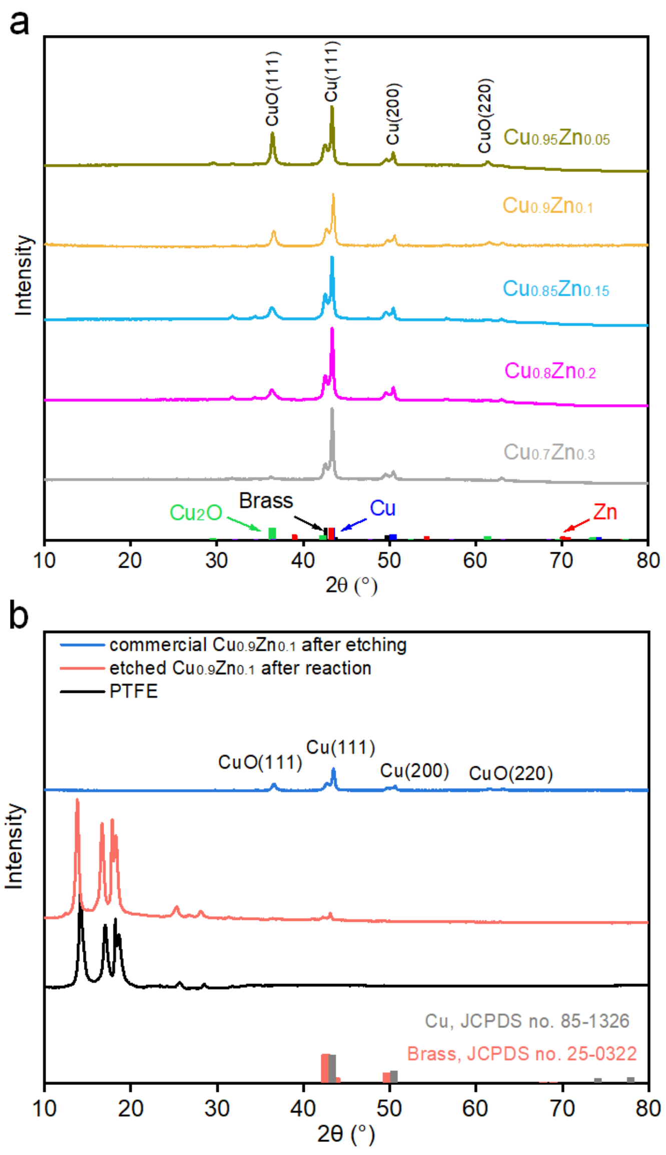

Supplementary Fig. 9 | (a) XRD patterns of Cu_xZn_{1-x} (x = 0.95, 0.9, 0.85, 0.8, 0.7) model catalysts fabricated by wet chemical etching of the commercial $Cu_{0.6}Zn_{0.4}$ powder (Sigma-Aldrich, Product No.: 593583-5G, <150 nm), (b) XRD patterns of wet-etched $Cu_{0.9}Zn_{0.1}$ catalysts before and after CO_2R. The XRD peaks correspond to Cu (JCPDS No. 04-0836) and brass (JCPDS No. 25-0322), which originated from the commercial $Cu_{0.6}Zn_{0.4}$ powder, and Cu_2O (JCPDS No. 05-0667) and ZnO (JCPDS No. 75-1526), which formed during the wet-etching process. Also, only metallic Cu diffraction peaks were observed with wet-etched $Cu_{0.9}Zn_{0.1}$ catalysts after CO_2R, in line with the Pourbaix diagram and suggesting that metallic Cu-Zn catalysts for CO_2R.

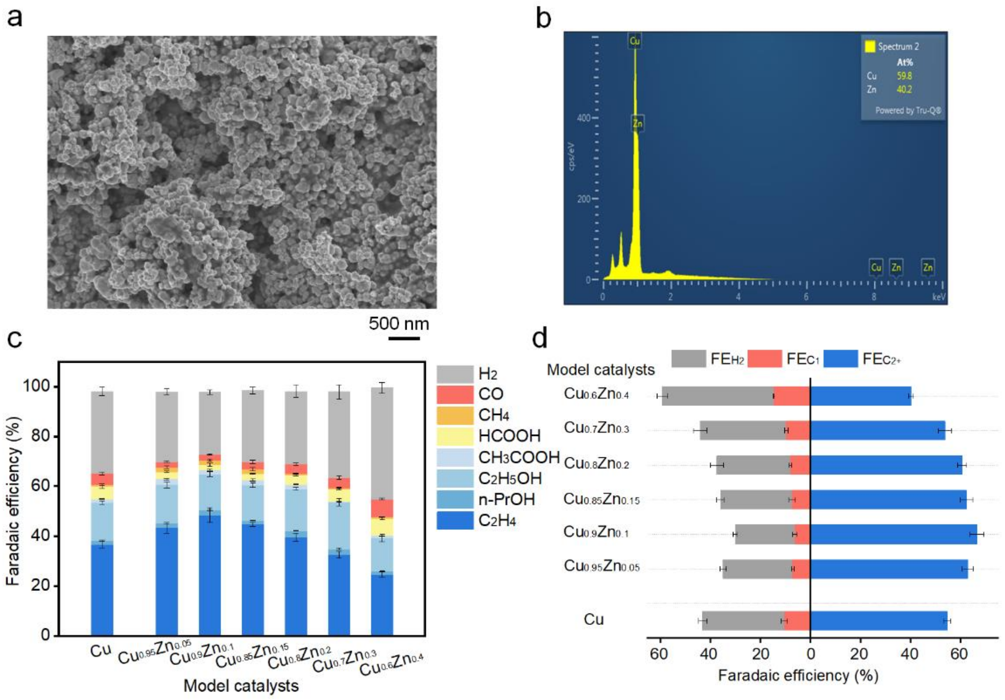

Supplementary Fig. 10 | (a–b) SEM and SEM-EDX results of the commercial $Cu_{0.6}Zn_{0.4}$ catalyst. Electrochemical CO_2R performance of the Cu_xZn_{1-x} (x = 0.95, 0.9, 0.85, 0.8, 0.7, 0.6) model catalysts fabricated by wet chemical etching of the commercial $Cu_{0.6}Zn_{0.4}$ powder (Sigma-Aldrich, Product No.: 593583-5G, <150 nm): (c) FEs of all products at 150 mA cm^{-2}, and (d) FEs of H_2, C_1, and C_{2+} products at 150 mA cm^{-2}. Error bars represent the standard deviation based on three independent measurements.

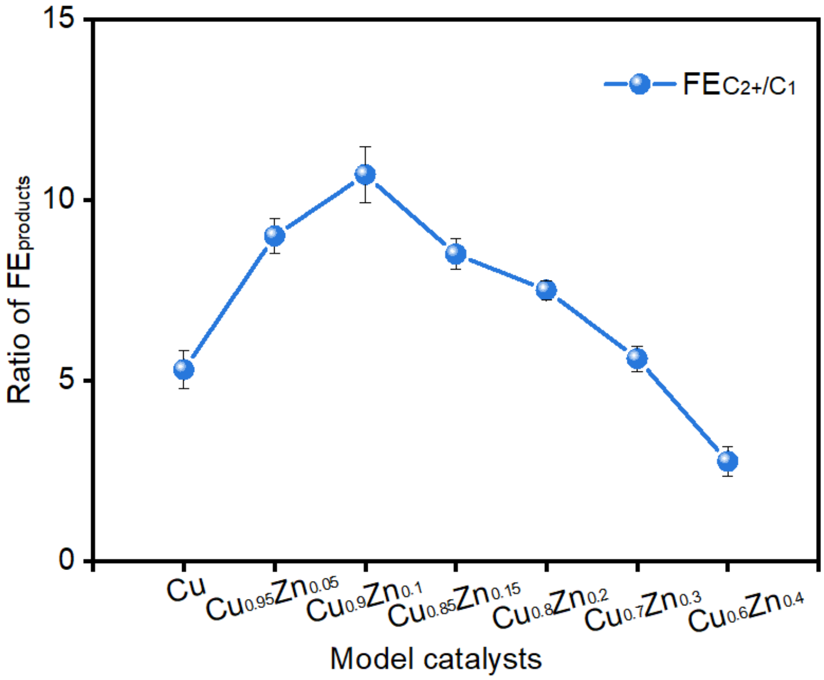

Supplementary Fig. 11 | C_{2+}/C_1 ratios of the Cu and Cu_xZn_{1-x} (x = 0.95, 0.9, 0.85, 0.8, 0.7, 0.6) model catalysts fabricated by wet chemical etching of the commercial $Cu_{0.6}Zn_{0.4}$ powder (Sigma-Aldrich, Product No.: 593583-5G, <150 nm). Error bars represent the standard deviation based on three independent measurements.

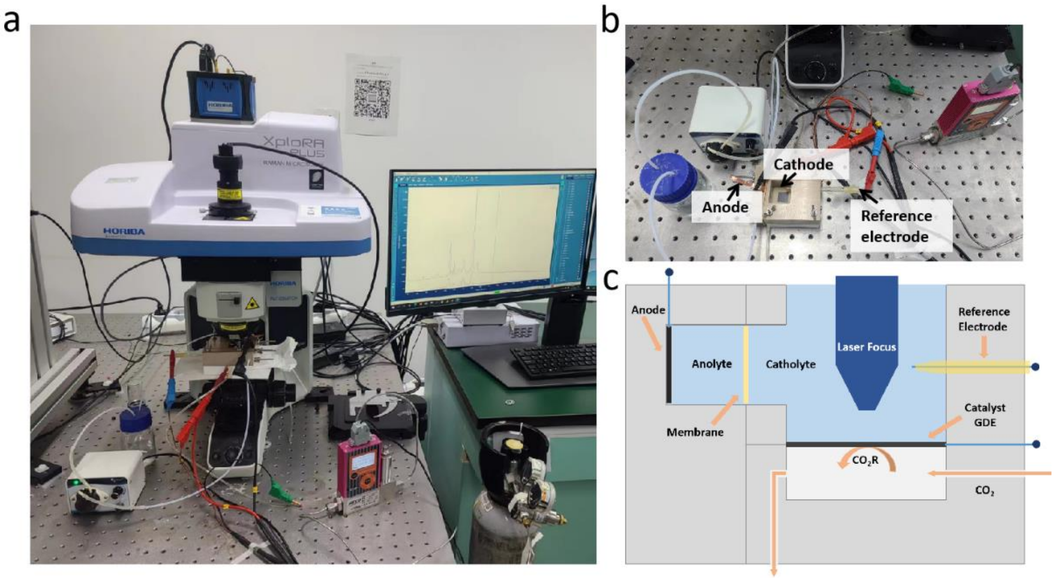

Supplementary Fig. 12 | (a–b) Photographs showing the experimental setup for *in situ* Raman measurement. (c) Schematic of the homemade flow cell with a three-electrode setup for carrying out *in situ* Raman study.

The Raman flow cell is composed of three chambers. CO_2 flows through the backside of the gas diffusion electrode in the gas chamber. Catholyte and anolyte were separated in two liquid chambers by an ion exchange membrane. In the electrolyte at pH 13.5, an anion exchange membrane (AEM, Fumasep FAB-PK-130, Fuel Cell Store) was used. In the electrolytes at pH 1, 4, 7, a cation exchange membrane (CEM, NafionTM 117, Fuel Cell Store) was used.

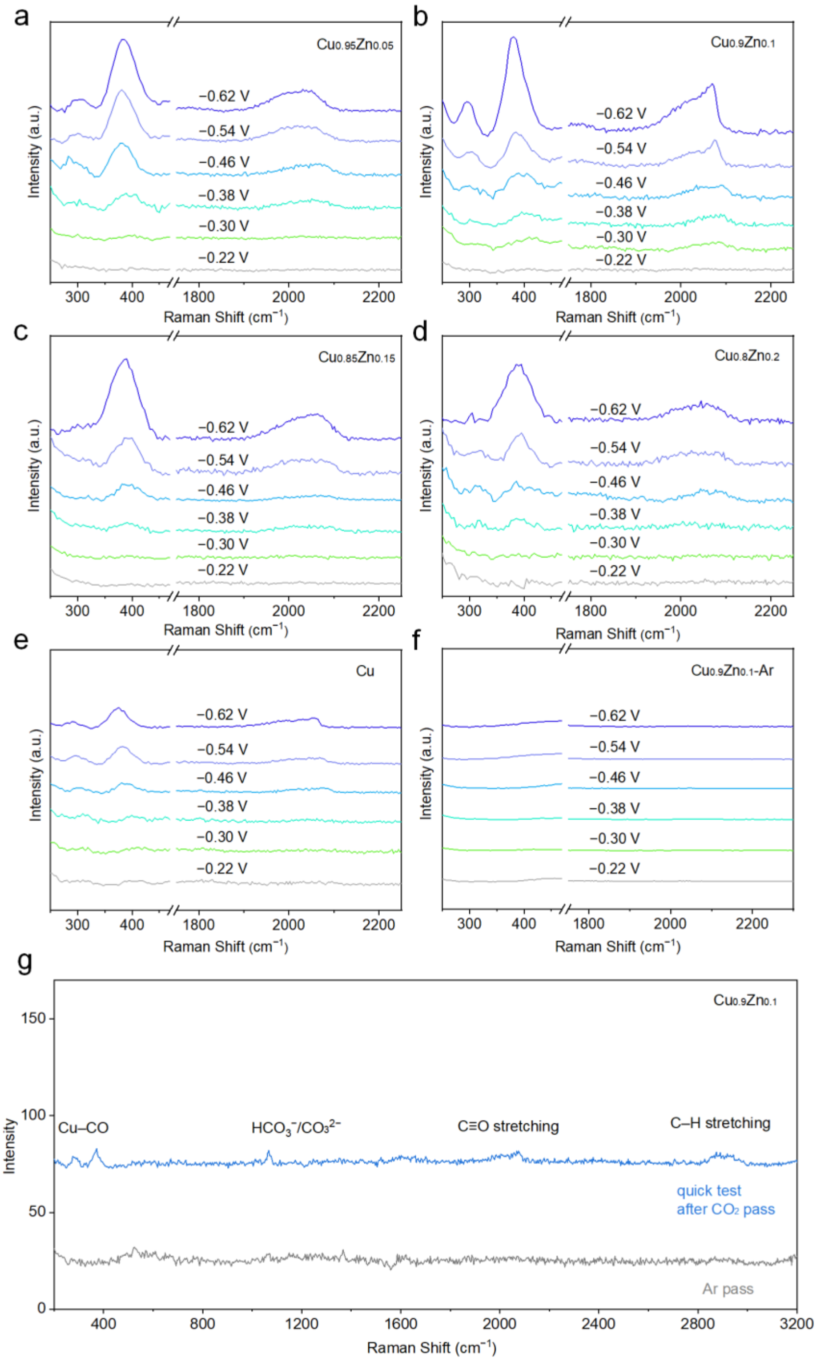

Supplementary Fig. 13 | *In situ* Raman spectra of nanoporous (a) $Cu_{0.95}Zn_{0.05}$, (b) $Cu_{0.9}Zn_{0.1}$, (c) $Cu_{0.85}Zn_{0.15}$, (d) $Cu_{0.8}Zn_{0.2}$, and (e) Cu catalysts during electrochemical CO_2R at various potentials. (f) *In situ* Raman spectra of $Cu_{0.9}Zn_{0.1}$ in Ar-saturated KOH at various applied potentials without flowing CO_2. (g) *In situ* Raman spectra of $Cu_{0.9}Zn_{0.1}$ at −0.38 V_{RHE} in Ar-saturated KOH (gray curve) and KOH right after switching Ar to CO_2 (blue curve). All potentials are with respect to the reversible hydrogen electrodes (RHE).

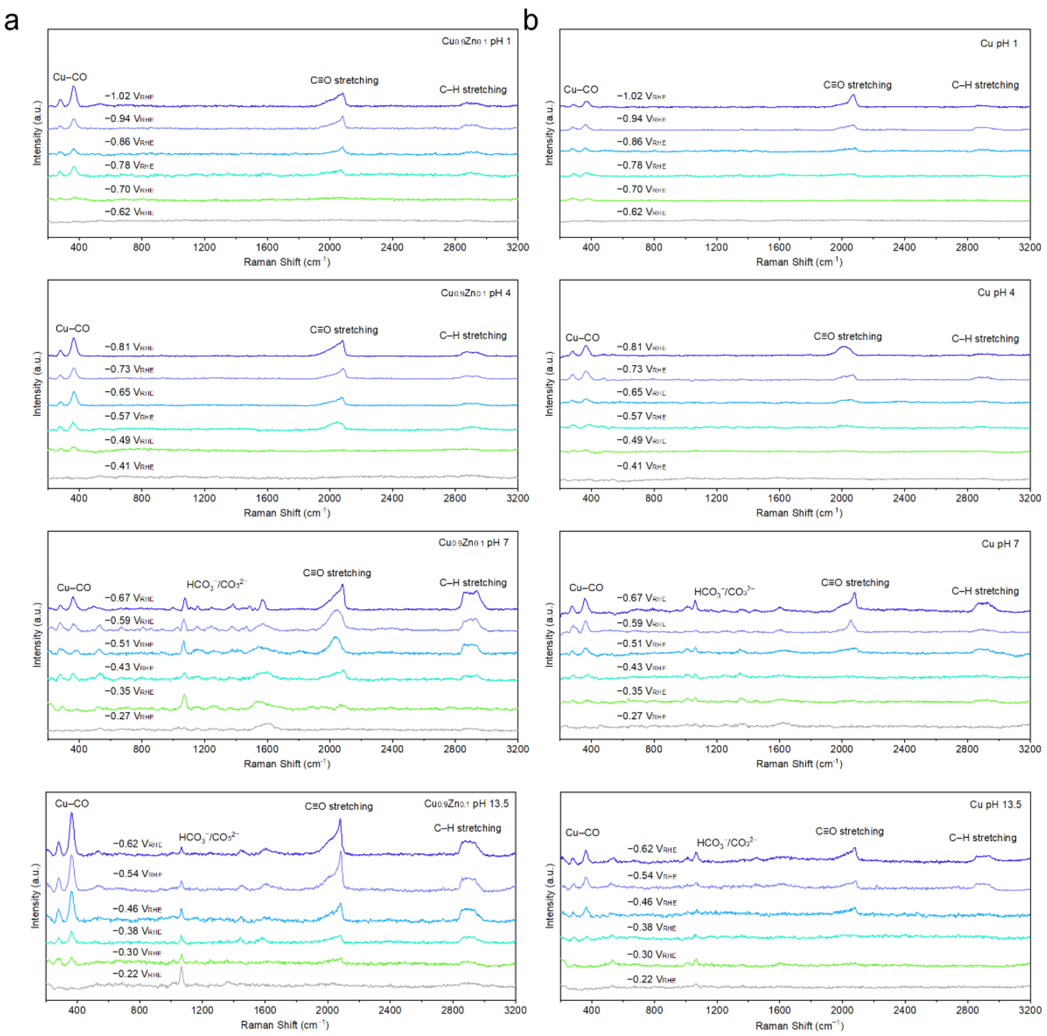

Supplementary Fig. 14 | *In situ* Raman spectra of (a) $Cu_{0.9}Zn_{0.1}$ and (b) Cu at different applied potentials in different electrolytes at pH 1, 4, 7, and 13.5.

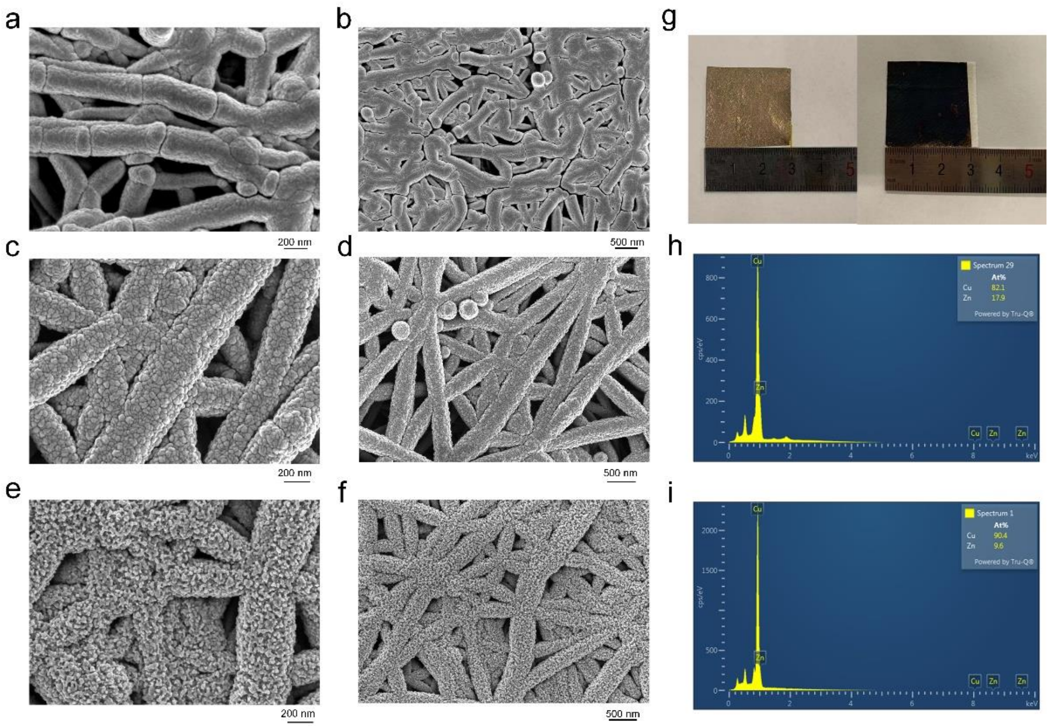

Supplementary Fig. 15 | (a, b) SEM images of sputtered pristine Cu catalysts (200 nm) on top of PTFE. SEM images of sputtered $Cu_{0.9}Zn_{0.1}$ catalysts (c, d) before and (e, f) after wet etching with corresponding (g) optical photograph of the co-sputtered $Cu_{0.9}Zn_{0.1}$ catalysts on PTFE before (left) and after (right) wet etching. EDS results for sputtered $Cu_{0.9}Zn_{0.1}$ catalysts (h) before and (i) after wet etching.

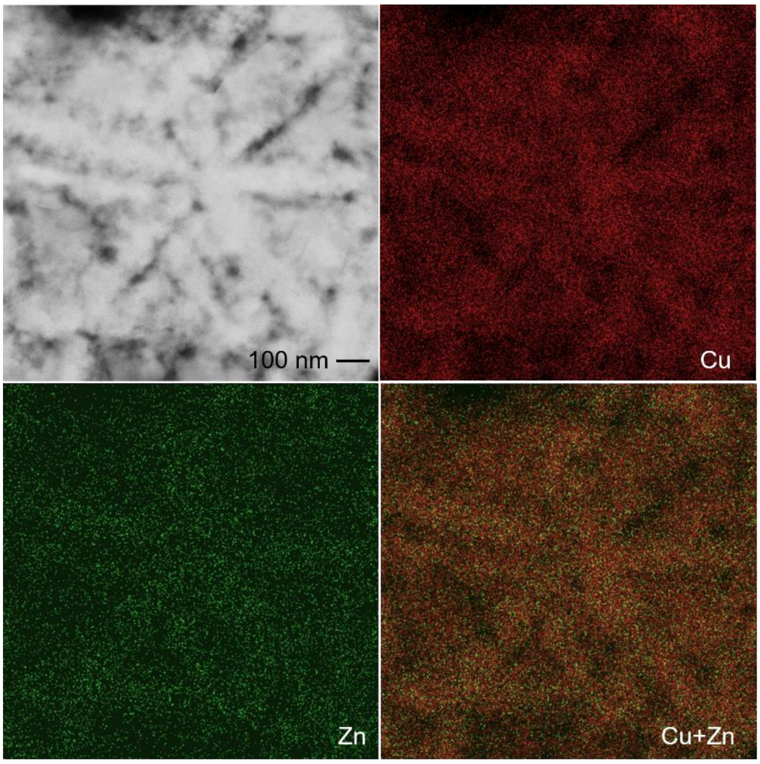

Supplementary Fig. 16 | STEM image and EDS elemental mapping results for the nanoporous $Cu_{0.9}Zn_{0.1}$ catalyst fabricated by co-sputtering and wet chemical etching.

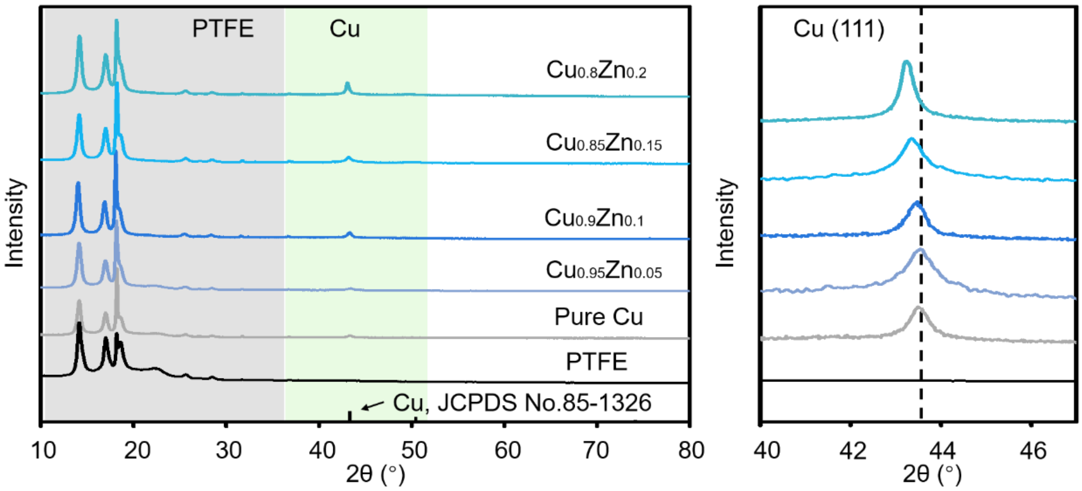

Supplementary Fig. 17 | XRD patterns of nanoporous Cu_yZn_{1-y} (y = 0.95, 0.9, 0.85, 0.8) and Cu catalysts fabricated by co-sputtering and wet chemical etching.

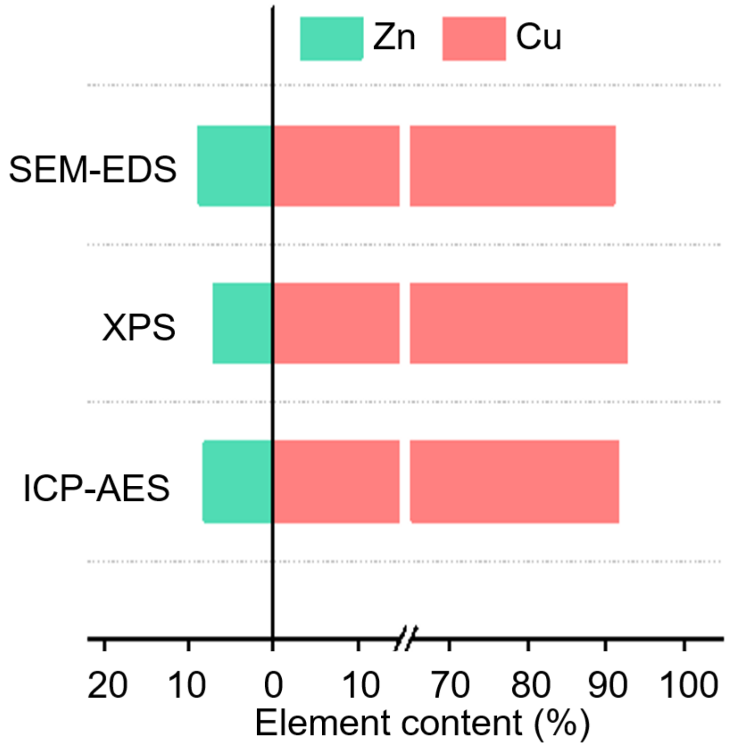

Supplementary Fig. 18 | Cu/Zn atomic ratio in $Cu_{0.9}Zn_{0.1}$ (fabricated by co-sputtering and wet chemical etching), as determined by SEM-EDS, XPS, and ICP-AES.

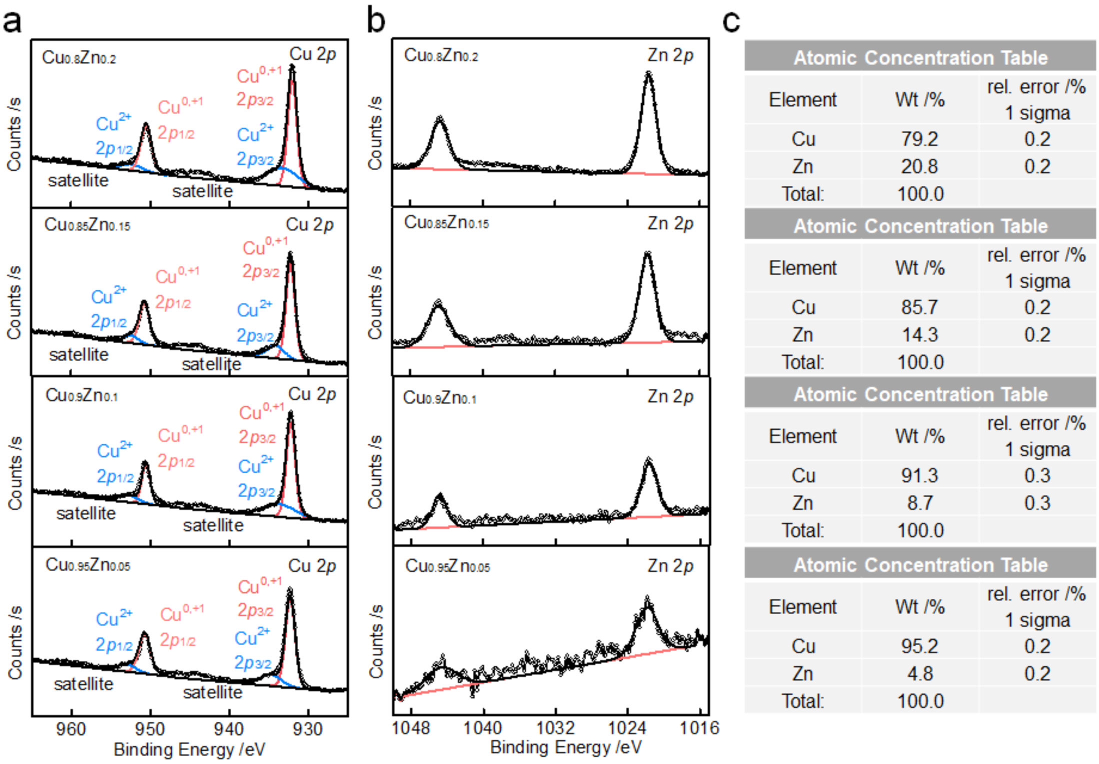

Supplementary Fig. 19 | XPS analysis of the nanoporous Cu_yZn_{1-y} (y = 0.95, 0.9, 0.85, 0.8) catalysts fabricated by co-sputtering and wet chemical etching: (a) Cu 2p spectra, (b) Zn 2p spectra, and (c) ratios of Cu and Zn.

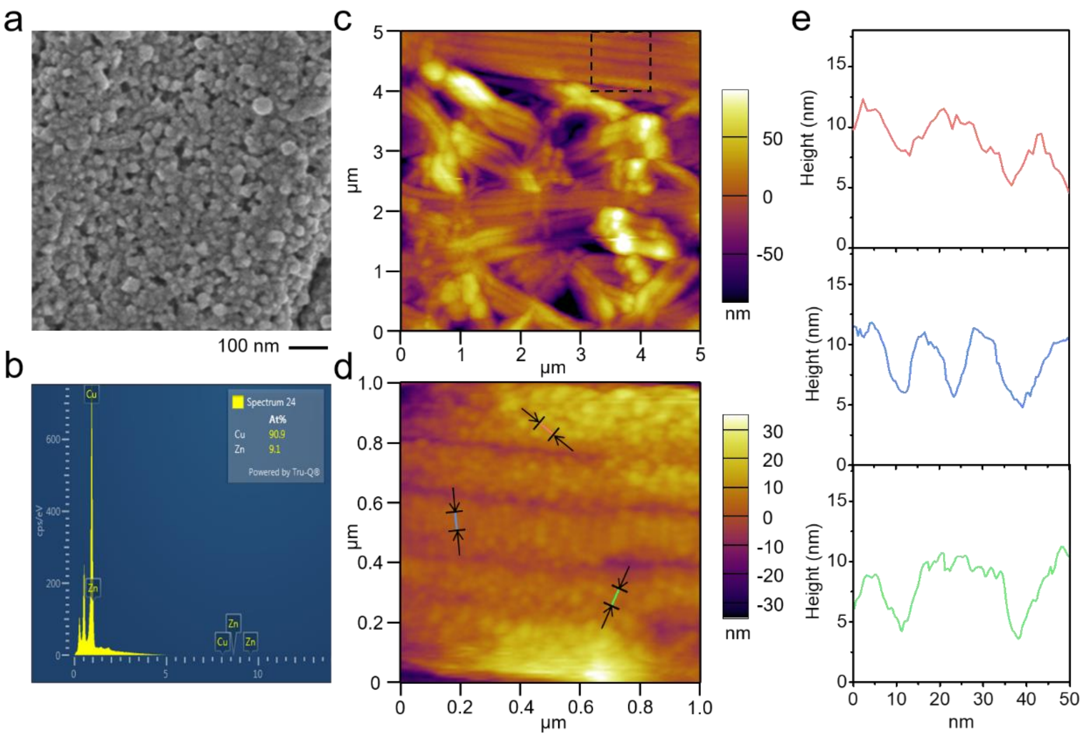

Supplementary Fig. 20 | (a) SEM image, (b) EDS spectrum, (c, d) atomic force microscopy image, and (e) corresponding pore diameter measurement for the nanoporous $Cu_{0.9}Zn_{0.1}$ catalyst (fabricated by co-sputtering and wet chemical etching) with 10 nm pores.

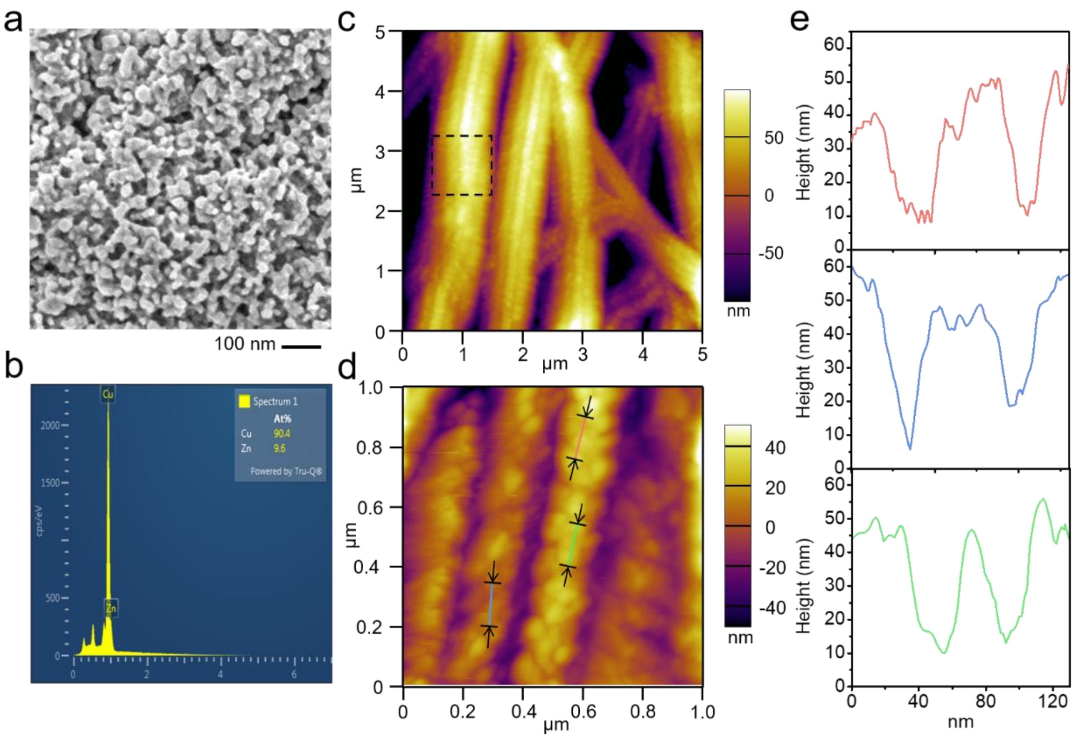

Supplementary Fig. 21 | (a) SEM image, (b) EDS spectrum, (c, d) atomic force microscopy image, and (e) corresponding pore diameter measurement for the nanoporous $Cu_{0.9}Zn_{0.1}$ catalyst (fabricated by co-sputtering and wet chemical etching) with 30 nm pores.

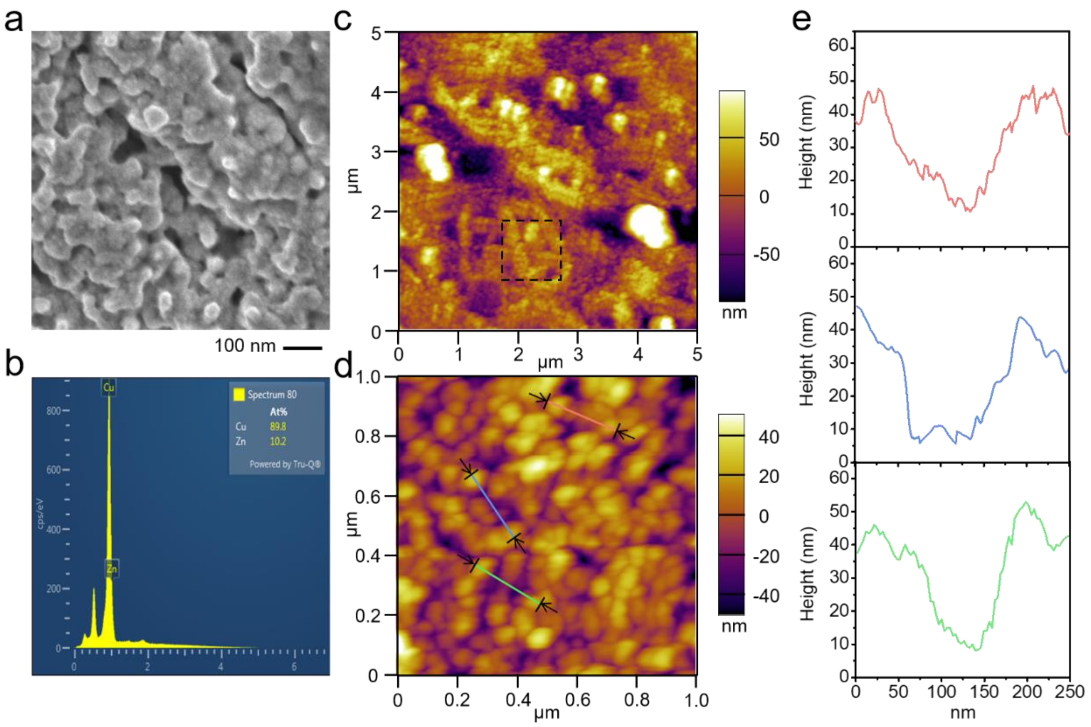

Supplementary Fig. 22 | (a) SEM image, (b) EDS spectrum, (c, d) atomic force microscopy image, and (e) corresponding pore diameter measurement for nanoporous $Cu_{0.9}Zn_{0.1}$ catalyst (fabricated by co-sputtering and wet chemical etching) with 150 nm pores.

EDS analysis showed that the surface and bulk Cu/Zn ratios were ~10 for all the nanoporous $Cu_{0.9}Zn_{0.1}$ catalysts (Supplementary Figs. 20–22). Atomic force microscopy was used to determine the pore sizes and depths of the various nanoporous $Cu_{0.9}Zn_{0.1}$ catalysts (10, 30, and 150 nm pores).

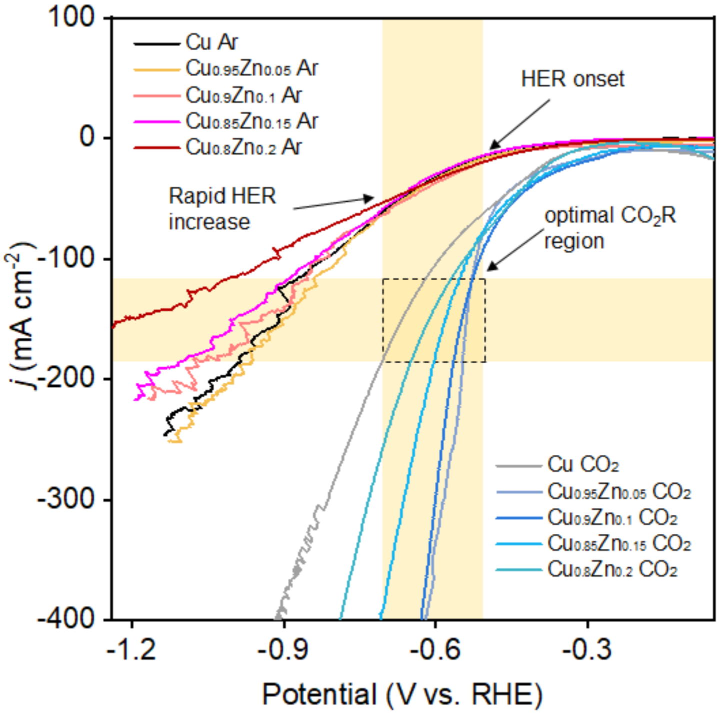

Supplementary Fig. 23 | The linear sweep voltammetry curve (LSV) in Ar-saturation and CO_2-saturation 0.75 M KOH electrolytes with Cu_yZn_{1-y} (y = 0.95, 0.9, 0.85, 0.8) and Cu catalysts (fabricated by co-sputtering and wet chemical etching).

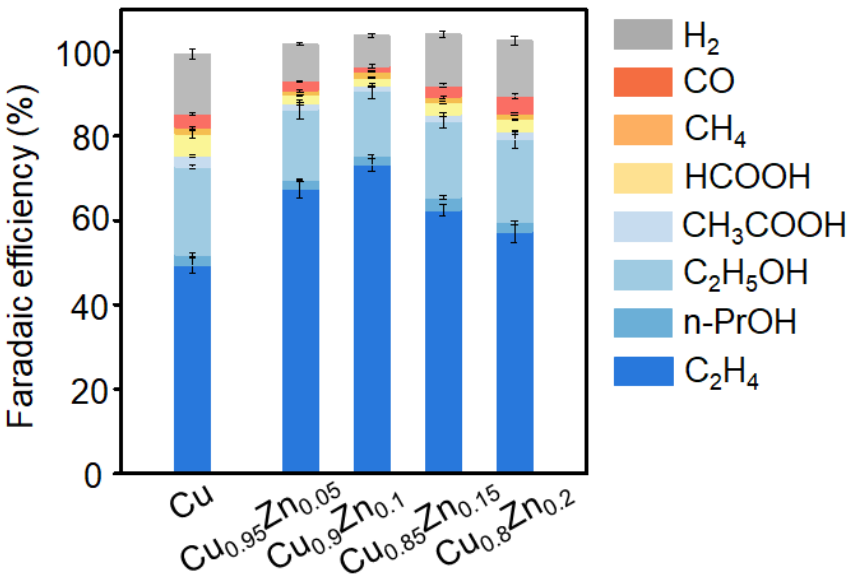

Supplementary Fig. 24 | Electrochemical CO_2R performance of the nanoporous Cu_yZn_{1-y} (y = 0.95, 0.9, 0.85, 0.8) catalysts (fabricated by co-sputtering and wet chemical etching) at 150 mA cm^{-2} current densities. Error bars represent the standard deviation based on three independent measurements.

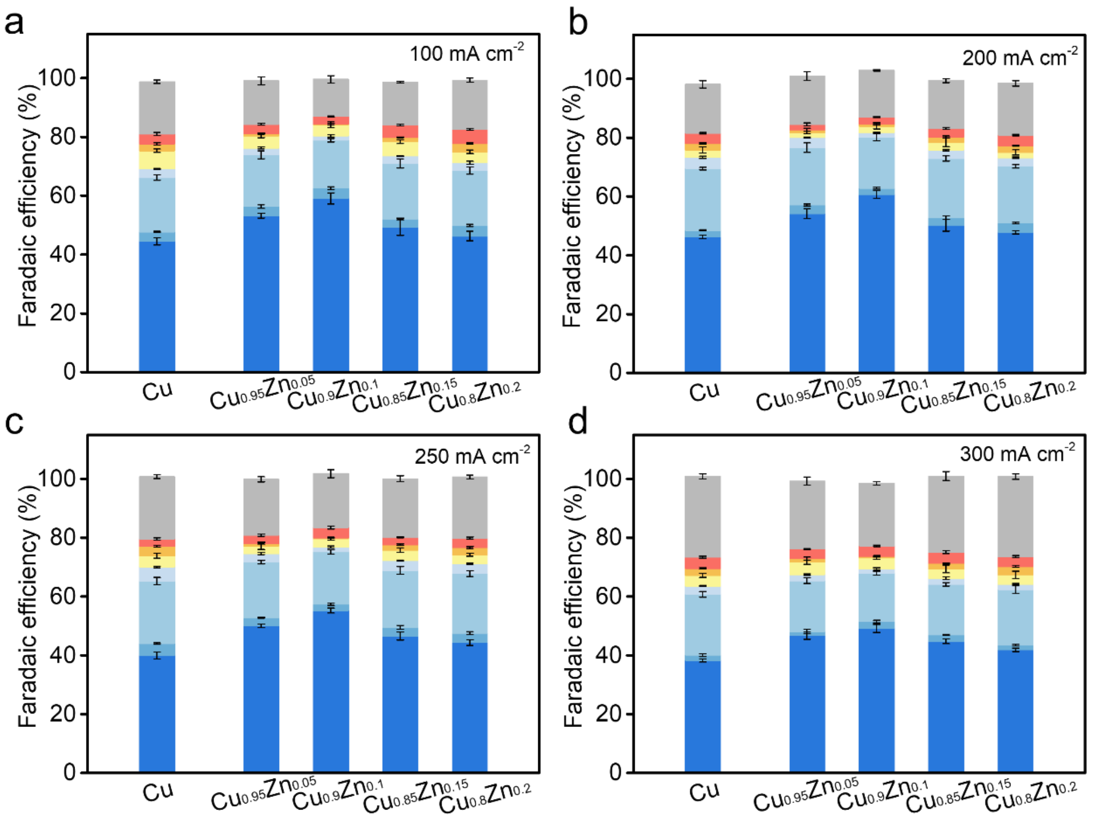

Supplementary Fig. 25 | Electrochemical CO_2R performance of the nanoporous Cu_yZn_{1-y} (y = 0.95, 0.9, 0.85, 0.8) catalysts (fabricated by co-sputtering and wet chemical etching) at different current densities: (a) 100 mA cm^{-2}, (b) 200 mA cm^{-2}, (c) 250 mA cm^{-2}, and (d) 300 mA cm^{-2}. Error bars represent the standard deviation based on three independent measurements.

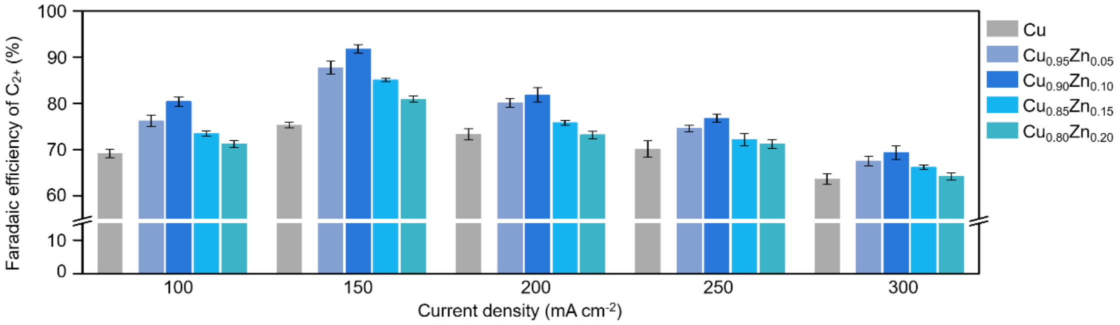

Supplementary Fig. 26 | FEs of the CO_2 reduction products with Cu_yZn_{1-y} (y = 0.95, 0.9, 0.85, 0.8) and Cu catalysts (fabricated by co-sputtering and wet chemical etching) at various current densities (100–300 mA cm^{-2}). Error bars represent the standard deviation based on three independent measurements.

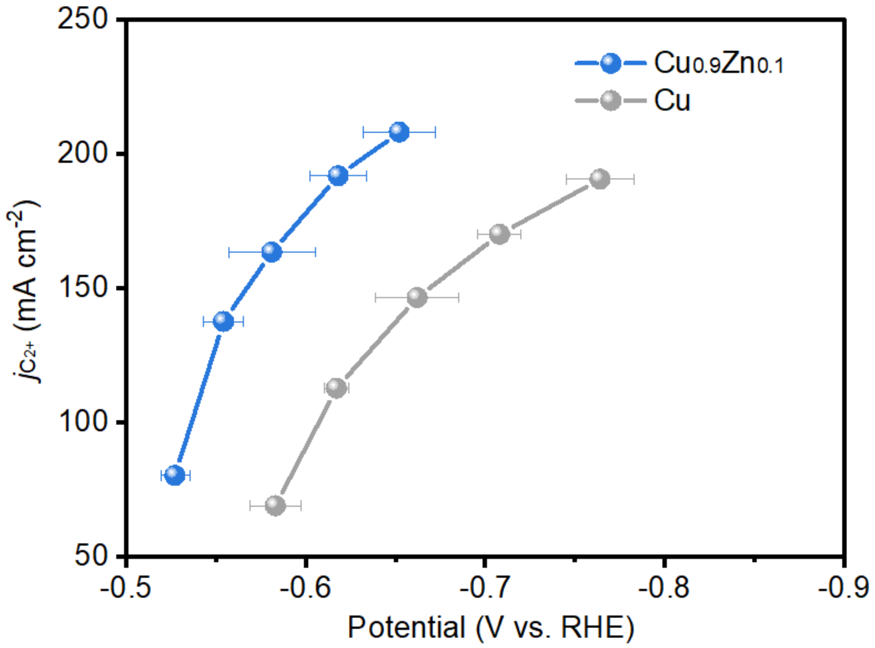

Supplementary Fig. 27 | Partial current densities for C_{2+} products versus using nanoporous $Cu_{0.9}Zn_{0.1}$ and Cu catalysts (fabricated by co-sputtering and wet chemical etching). Error bars represent the standard deviation based on three independent measurements.

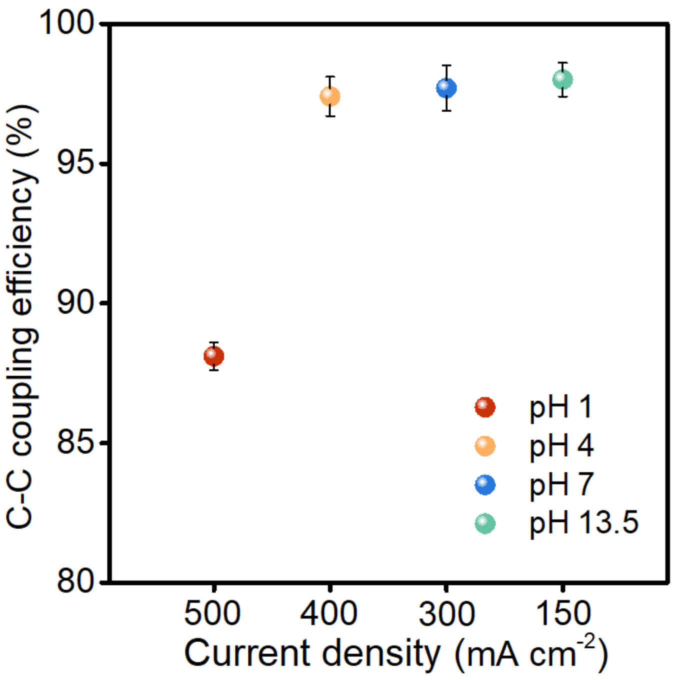

Supplementary Fig. 28 | C–C coupling efficiency at different current densities in pH 1, 4, 7 and 13.5 electrolytes. Error bars represent the standard deviation based on three independent measurements.

C–C coupling efficiency was defined as the amount of CO_2 consumed to produce C_{2+} versus the amount of CO_2 consumed to produce all C_1–C_3 products.

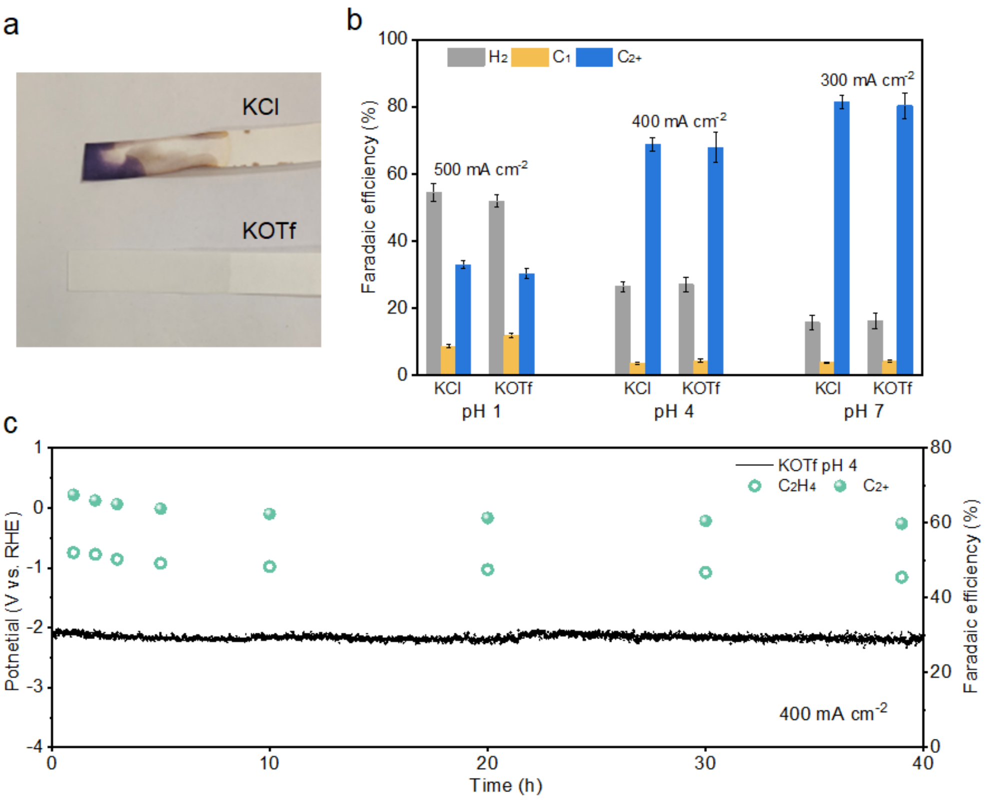

Supplementary Fig. 29 | (a) Optical images of the starch potassium iodide test papers to detect Cl_2 generation on the anode side under the conditions: (1) CO_2R with 3 M KCl anolyte (pH 1) at 500 mA cm^{-2}, and (2) CO_2R with 3 M KOTf (pH 1) at 500 mA cm^{-2}. (b) The CO_2R performance at pH 1, 4, and 7 in 3 M KCl and 3 M KOTf catholytes, respectively. The CO_2R experiments were carried out at optimized current densities: 500 mA cm^{-2} at pH 1, 400 mA cm^{-2} at pH 4, and 300 mA cm^{-2} at pH 7. Error bars represent the standard deviation based on three independent measurements. (c) Electrochemical CO_2R stability curves (black line) and ethylene and C_{2+} FEs (hollow sphere and solid sphere) of the nanoporous $Cu_{0.9}Zn_{0.1}$ catalyst (fabricated by co-sputtering and wet chemical etching) at 400 mA cm^{-2} in 3 M KOTf electrolyte (pH 4).

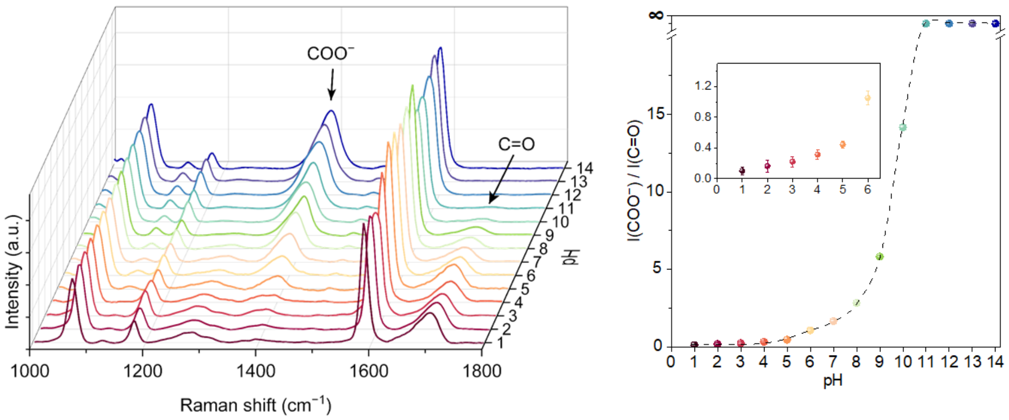

Supplementary Fig. 30 | Raman spectra of *p*-MBA and the peak intensity ratio of COO^- (1393 cm^{-1}) and C=O (1702 cm^{-1}) at pH from 1 to 14. The peak intensities were calibrated according to the intensity of the normalized 1077 cm^{-1} band. Error bars represent the standard deviation based on three independent measurements.

According to the report by Halas and co-workers, *para*-mercaptobenzoic acid (*p*-MBA) can be used as a nanoscale pH meter to monitor the local pH near the electrode surface during the electrochemical reactions. At low pH, the carboxylate group in *p*-MBA was mostly protonated, and at high pH values, the carboxylate group was mostly deprotonated. These changes in molecular structure were reflected in the Raman spectra, specifically the bands at 1393 cm^{-1} (COO^-) and 1702 cm^{-1} (C=O). Thus, the pH value can be obtained from the intensity ratio of the two peaks: (COO^-)/(C=O). The (COO^-)/(C=O) ratios as a function of the electrolyte pH were plotted as a calibration curve via measured the Raman spectra of *p*-MBA in electrolytes at different pH from 1 to 14. The (COO^-)/(C=O) ratios at pH 4–10 showed good reproducibility, while it varied largely at pH < 4 or pH > 10. Hence, we measured the surface pH on electrodes in the range of pH 4–10 during CO_2R.

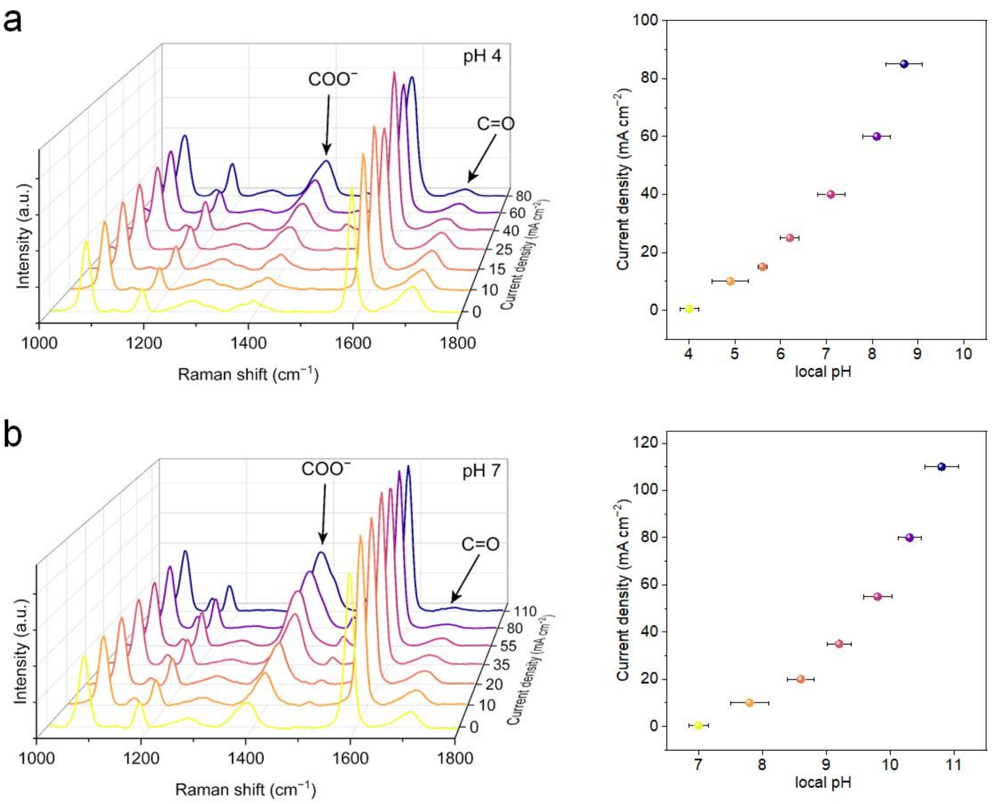

Supplementary Fig. 31 | Raman spectra of the *p*-MBA coated $Cu_{0.9}Zn_{0.1}$ during CO_2R at different current densities at (a) pH 4 and (b) pH 7 and the estimated surface pH at different current densities. The peak intensities were all normalized to that of the 1077 cm^{-1} band. Error bars represent the standard deviation based on three measurements.

We coated $Cu_{0.9}Zn_{0.1}$ with *p*-MBA, and performed *in situ* Raman observation during CO_2R at pH 4 and 7 at different current density. The intensity of $(COO^-)/(C{=}O)$ ratios as a function of the current densities in the range of 0–80 mA cm^{-2} to estimate the surface pH. We confirmed that the surface pH increased linearly with the increase of current density. An increase in local pH could increase the surface potential versus standard hydrogen electrode (SHE), which thus increased the electrical field on the electrode surface. As a result, we observed increased CO_2R performance with the increase of applied potential.

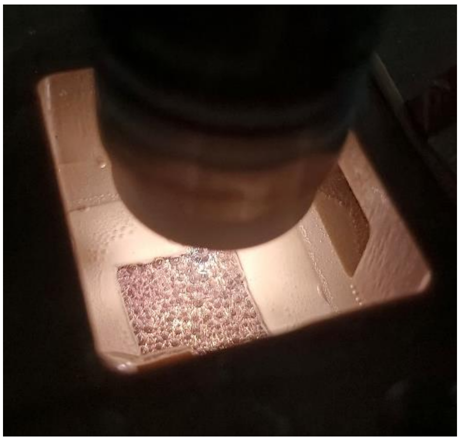

Supplementary Fig. 32 | The optical photograph of a $Cu_{0.9}Zn_{0.1}$ electrode during CO_2R at >80 mA cm^{-2}. Large amounts of bubbles appeared on the catalyst surfaces.

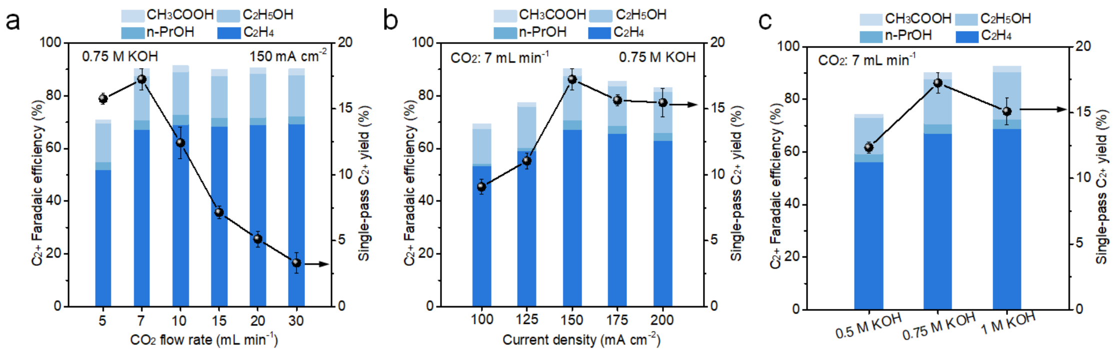

Supplementary Fig. 33 | C_{2+} FE and single-pass C_{2+} yield for the nanoporous $Cu_{0.9}Zn_{0.1}$ catalyst (fabricated by co-sputtering and wet chemical etching) under various test conditions: (a) different CO_2 flow rates, (b) different current densities and (c) different electrolytes. Error bars represent the standard deviation based on three independent measurements.

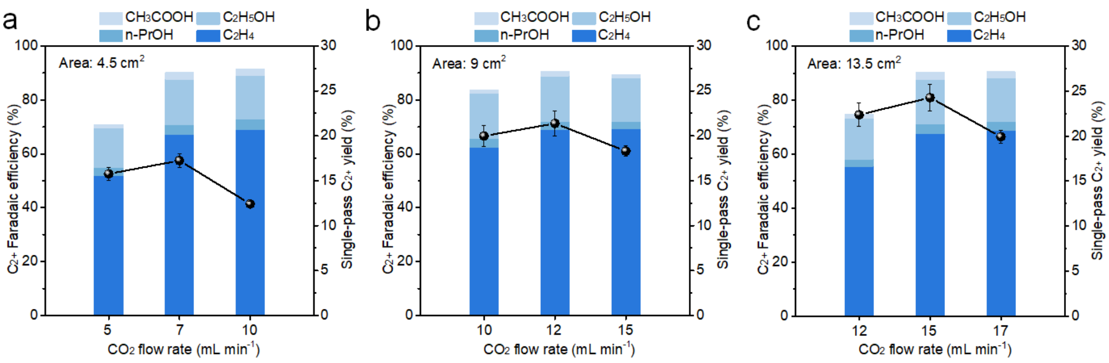

Supplementary Fig. 34 | Single-pass C_{2+} yield and C_{2+} FE for the nanoporous $Cu_{0.9}Zn_{0.1}$ catalyst (fabricated by co-sputtering and wet chemical etching) in serpentine-channel cells: (a) 4.5 cm^2, (b) 9 cm^2, and (c) 13.5 cm^2. Error bars represent the standard deviation based on three independent measurements.

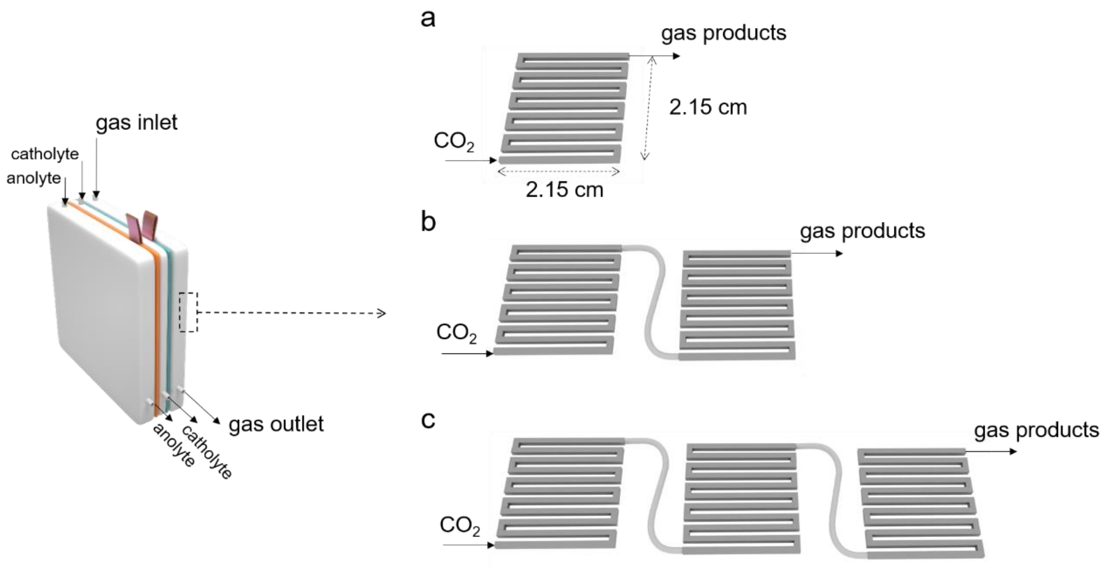

Supplementary Fig. 35 | Schematic of the CO_2R cells: (a) 4.5 cm^2, (b) 9 cm^2, (c) 13.5 cm^2. The dimension of the serpentine channel is 1 mm × 1 mm × 21.5 mm.

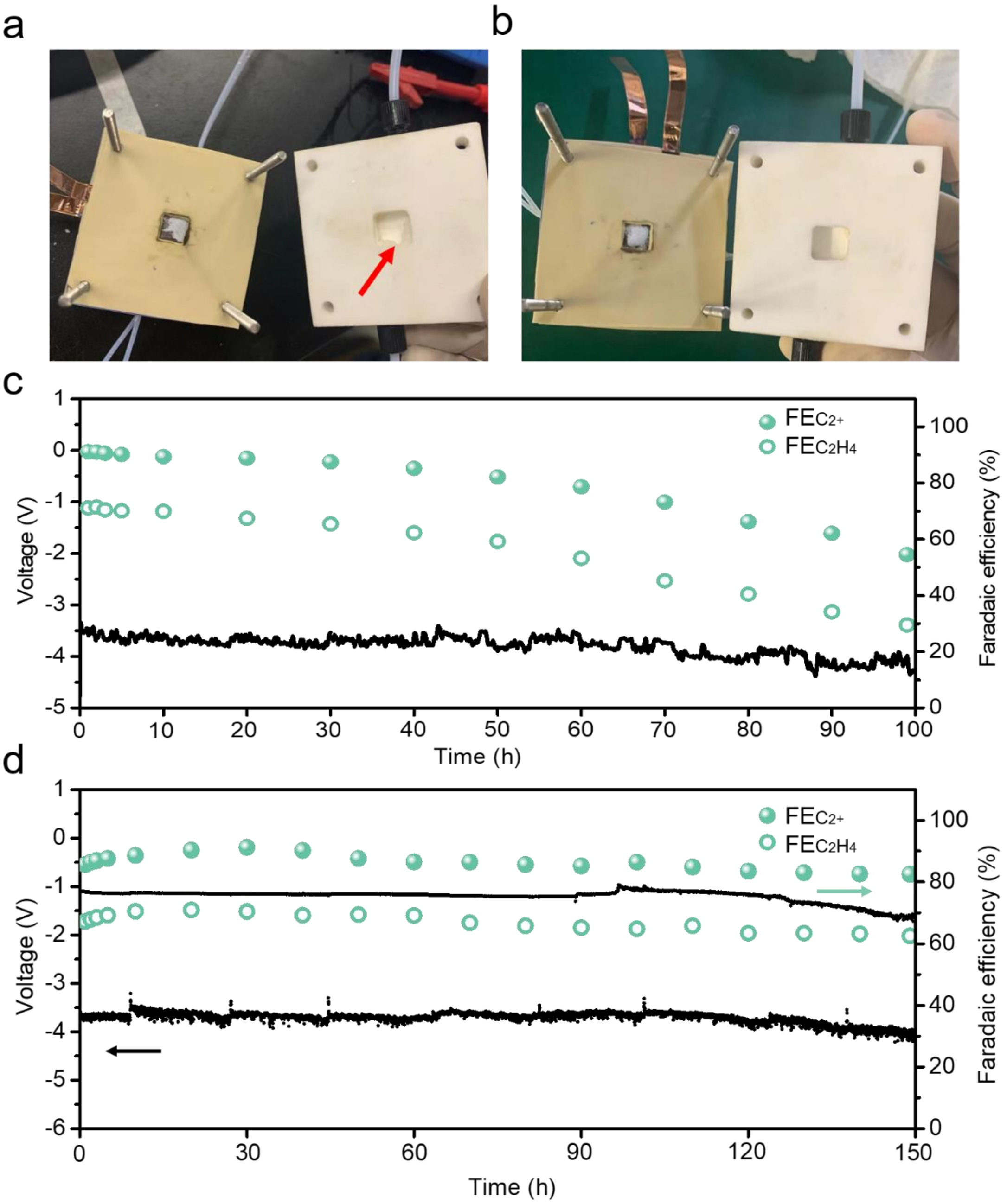

Supplementary Fig. 36 | Optical photographs showing (a) carbonate precipitation on the backside of the PTFE substrate and in the cathodic chamber of the flow cell device after the 100 hours stability test using conventional chronopotentiometry, and (b) no carbonate precipitation after the 150 hours stability test using an alternating regenerative current density. Electrochemical CO_2R stability curves and ethylene and C_{2+} FEs of the graphite/carbon nanoparticles/$Cu_{0.9}Zn_{0.1}$/PTFE catalysts at 150 mA cm^{-2} in 0.75 M KOH electrolyte obtained using (c) conventional chronopotentiometry and (d) alternating regenerative current densities.

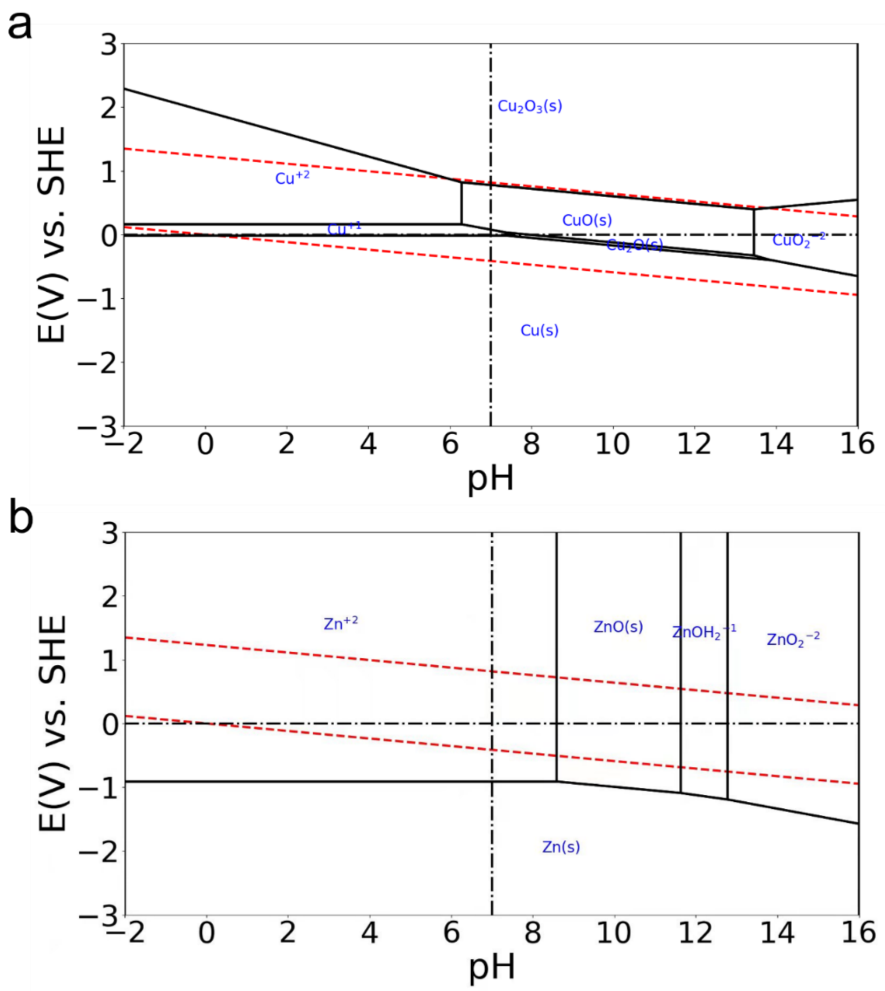

Supplementary Fig. 37 | Pourbaix diagrams of (a) Cu and (b) Zn.

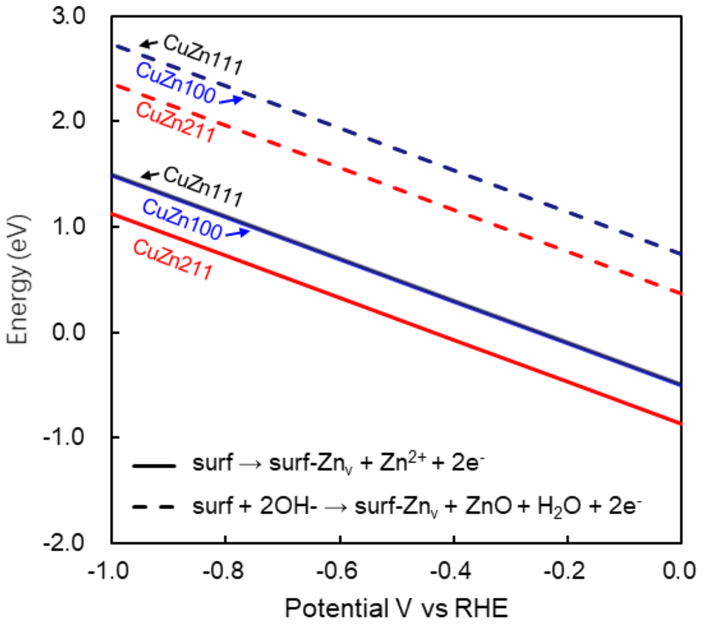

Supplementary Fig. 38 | Stability of CuZn surfaces described by the solution energy of Zn (solid lines) and formation energy of ZnO (dashed lines) at different electrode potentials. The solution energy was calculated following the conversion of i) "surf → surf-Zn_v + Zn" where the energy of Zn was referenced to Zn bulk, and ii) "Zn → Zn^{2+} + $2e^-$" where the energy change was calculated based on the redox potential. The formation energy was calculated following the conversion of i) "surf + $2OH^-$ → surf-Zn_v + ZnO + H_2O + $2e^-$" where the formation energy of ZnO was referenced to ZnO bulk, and the energy change of "$2OH^-$ → H_2O + $2e^-$" was calculated based on the approximation of the computational hydrogen electrode (CHE) model.

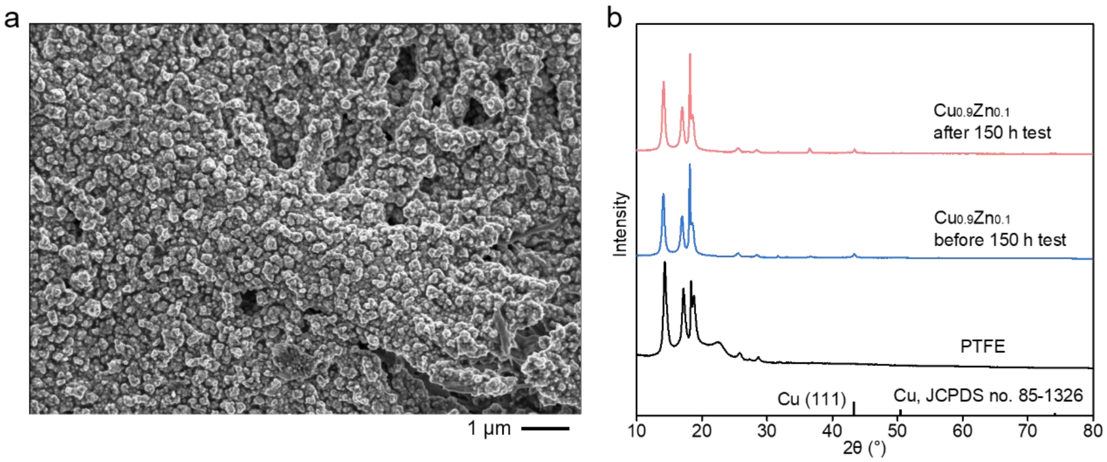

Supplementary Fig. 39 | (a) SEM image and (b) XRD patterns of the nanoporous $Cu_{0.9}Zn_{0.1}$ catalyst (fabricated by co-sputtering and wet chemical etching) after the 150 hours stability test.

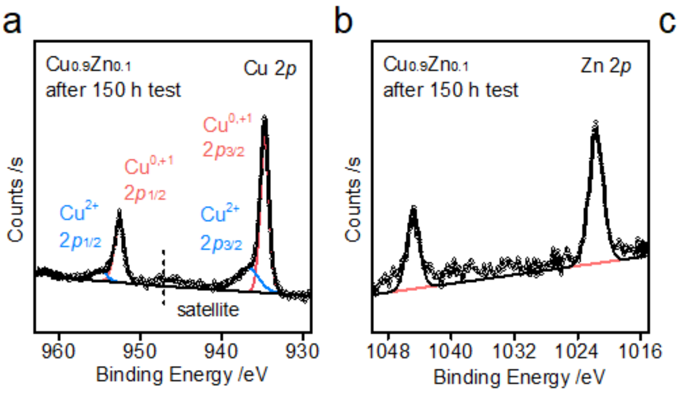

c

Atomic Concentration Table		
Element	Wt /%	rel. error /% 1 sigma
Cu	91.1	0.2
Zn	8.9	0.2
Total:	100.0	

Supplementary Fig. 40 | XPS analysis of the nanoporous $Cu_{0.9}Zn_{0.1}$ catalyst (fabricated by co-sputtering and wet chemical etching) after the 150 hours stability test: (a) Cu 2*p* spectra, (b) Zn 2*p* spectra, and (c) ratios of Cu and Zn.

The origin data of gas chromatography and NMR were supplied in Supplementary Figs. 41–47.

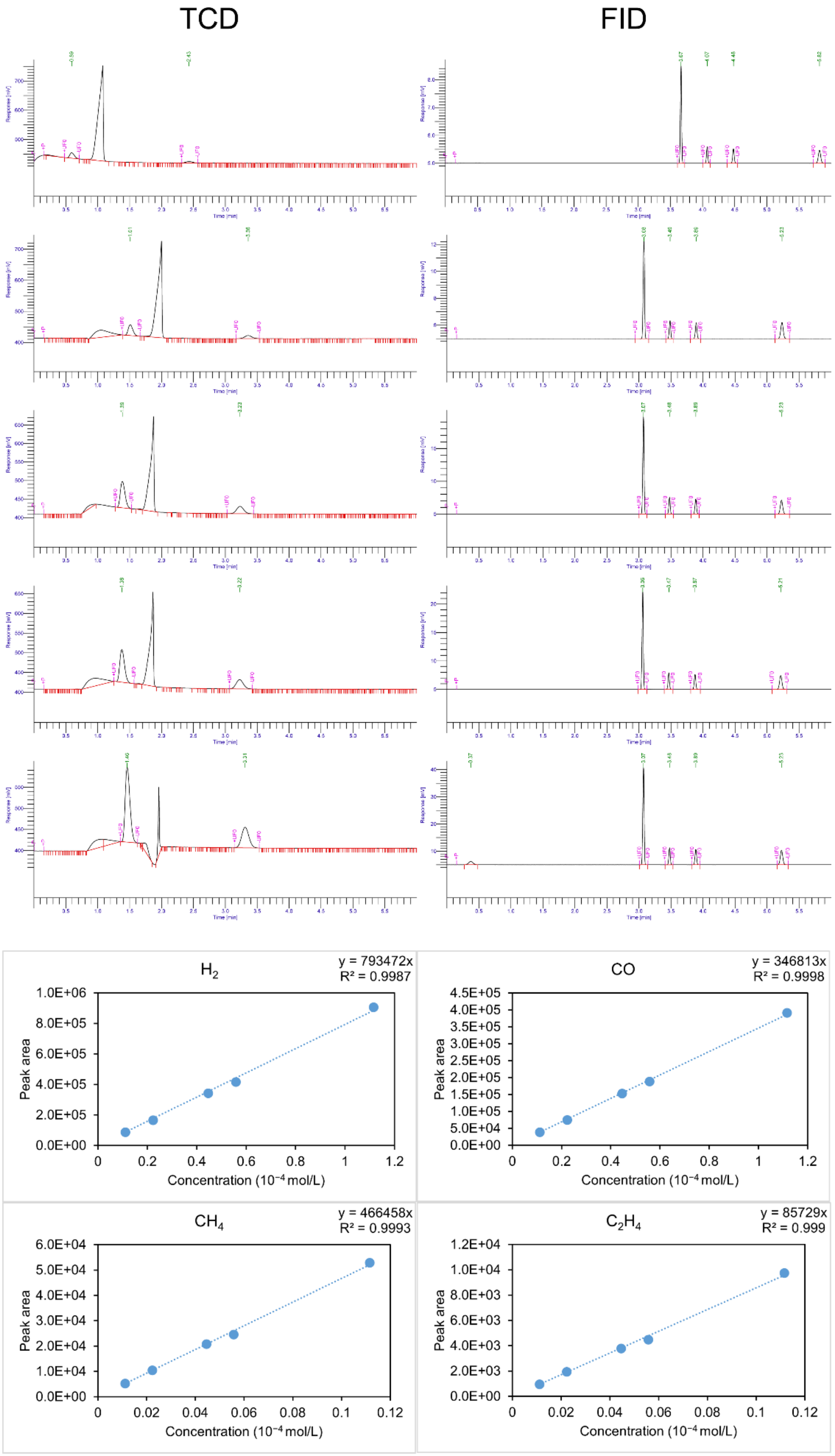

Supplementary Fig. 41 | The gas chromatography (Perkin Elmer Clarus 680) calibration curves of gaseous products.

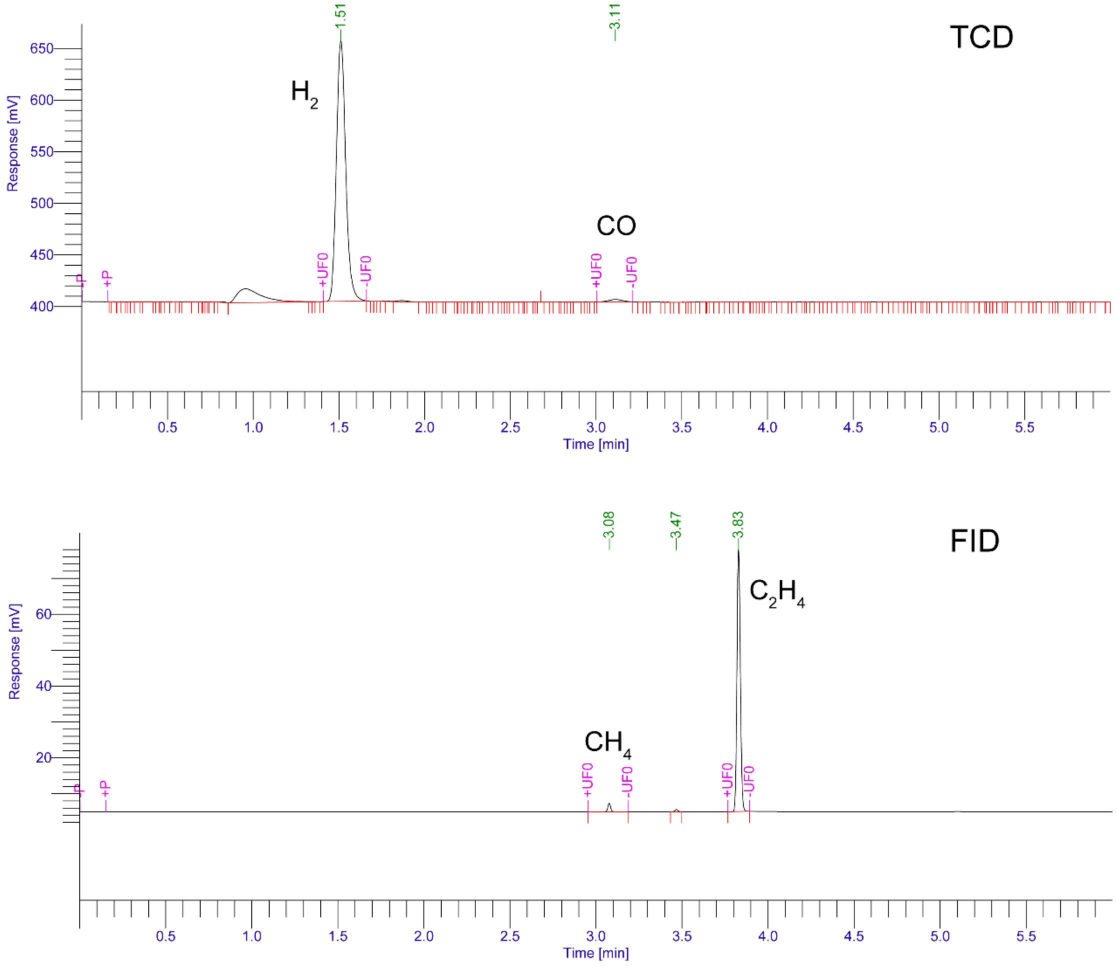

Supplementary Fig. 42 | The gas chromatography (Perkin Elmer Clarus 680) data of $Cu_{0.9}Zn_{0.1}$ (fabricated by co-sputtering and wet chemical etching) with the best C_2H_4 FE at pH 13.5 electrolyte on 150 mA cm^{-2} current density.

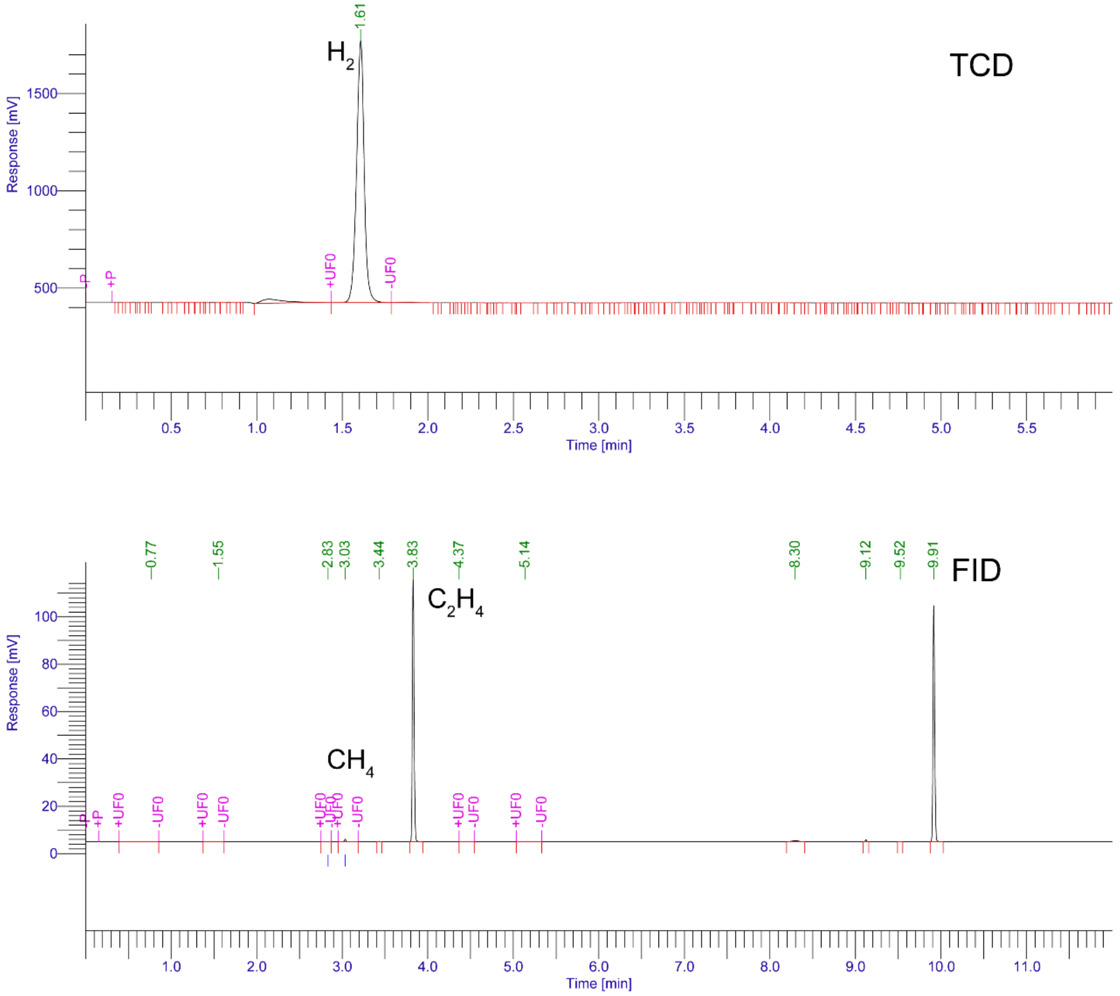

Supplementary Fig. 43 | The gas chromatography (Perkin Elmer Clarus 680) data of $Cu_{0.9}Zn_{0.1}$ (fabricated by co-sputtering and wet chemical etching) with the best C_2H_4 FE at pH 4 electrolyte on 400 mA cm^{-2} current density.

Supplementary Table 1 | Calculated catalytic performance based on the raw data acquired by Perkin Elmer Clarus 680 is listed as follows:

Catalyst	j (mA cm^{-2})	pH	Product			
			H_2 (%)	CH_4 (%)	CO (%)	C_2H_4 (%)
$Cu_{0.9}Zn_{0.1}$	400	4	FE*: 26.5	FE: 0.1	FE: 1.5	FE: 52.1
$Cu_{0.9}Zn_{0.1}$	150	13.5	FE: 7.3	FE: 0.5	FE: 1.3	FE: 73.2

* FE represents Faradaic efficiency;

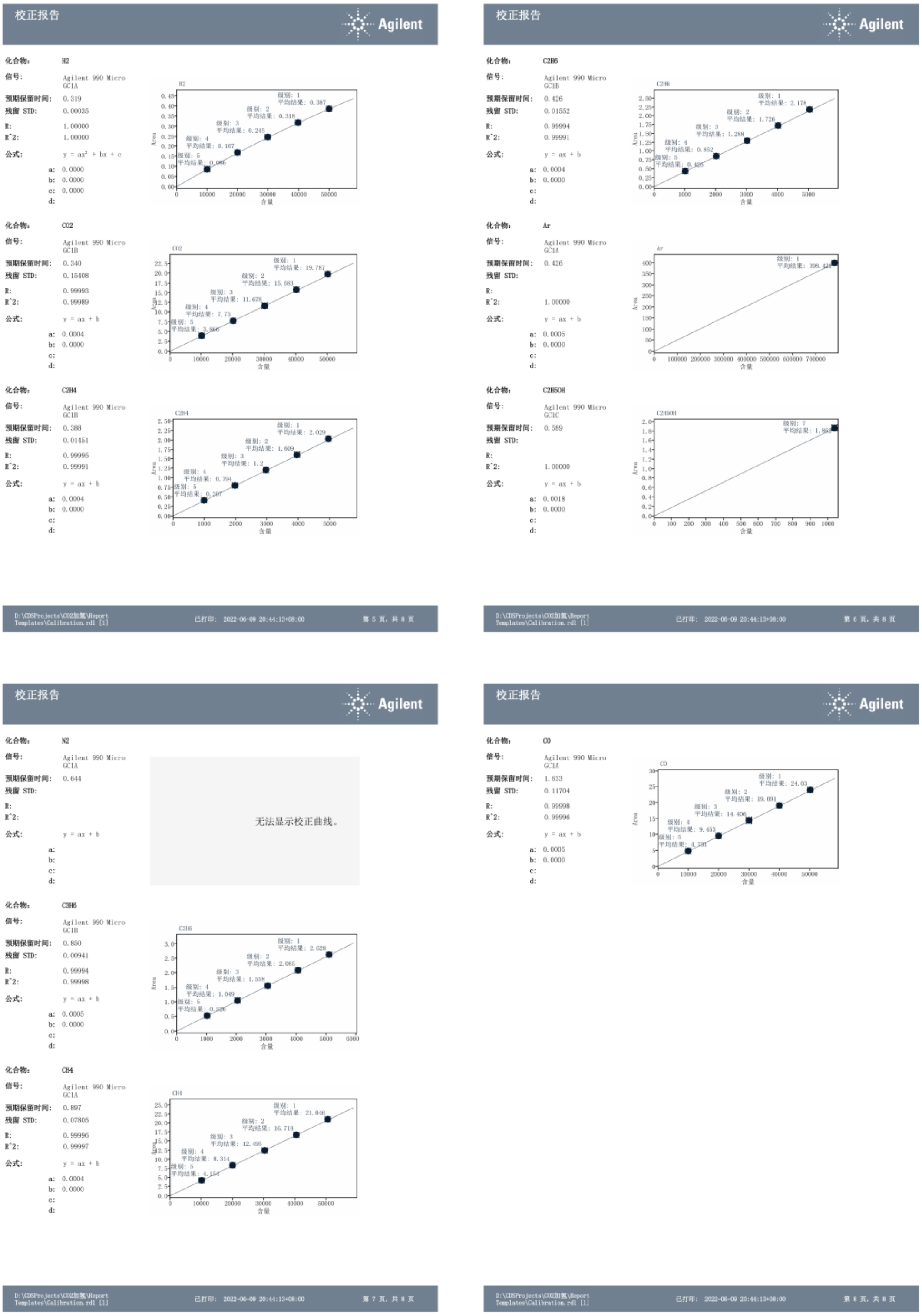

Supplementary Fig. 44 | The gas chromatography (Agilent 990 Micro) calibration curves of gaseous products.

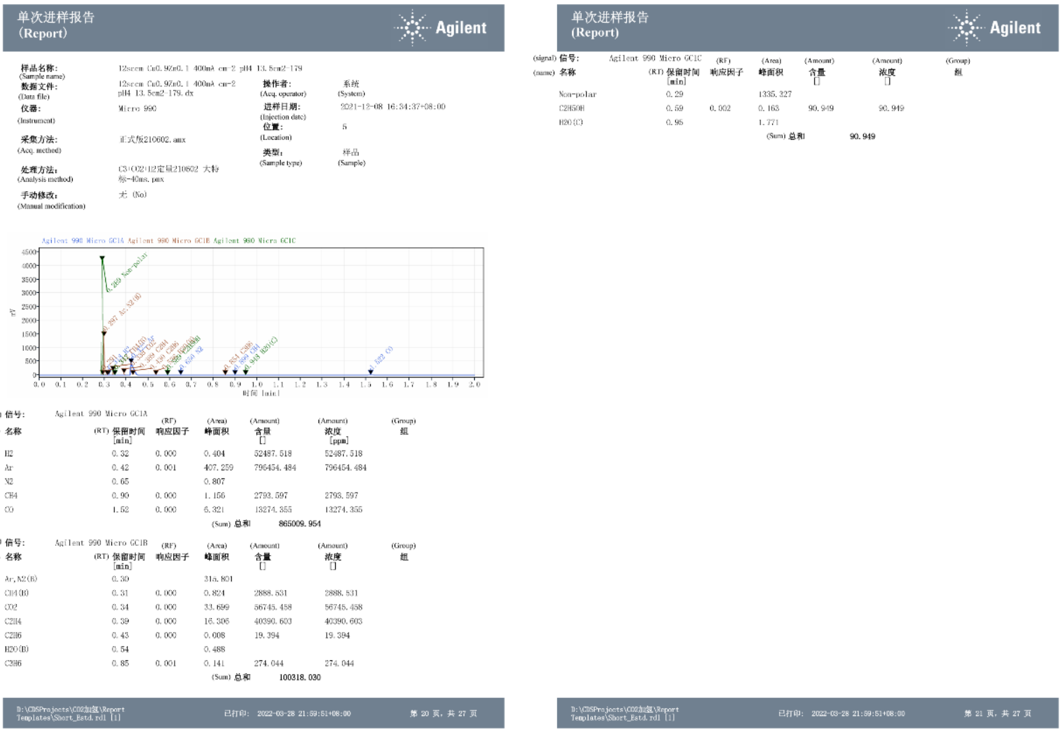

Supplementary Fig. 45 | The gas chromatography (Agilent Micro 990) data of $Cu_{0.9}Zn_{0.1}$ (fabricated by co-sputtering and wet chemical etching) at pH 4 electrolyte on 400 mA cm^{-2} current density at a flow rate of 12 sccm.

Supplementary Table 2 | Calculated catalytic performance based on the raw data acquired by Agilent Micro 990 GC system is listed as follows:

Catalyst	j (mA cm^{-2})	pH	Product			
			H_2* (%)	CH_4 (%)	CO (%)	C_2H_4 (%)
$Cu_{0.9}Zn_{0.1}$	400	4	FE**:11.9<br>SPY***:/	FE: 3.0<br>SPY: 0.8	FE: 2.5<br>SPY: 2.9	FE: 55.0<br>SPY: 24.2

* The amount of H_2 was overestimated due to the use of He carrier gas for the µTCD detector in the Agilent Micro GC 990 system;
** FE represents Faradaic efficiency;
*** SPY represents single-pass yield.

Supplementary Table 3 | C_{2+} SPY of $Cu_{0.9}Zn_{0.1}$ was calculated as: $SPY_{C2+} = SPY_{C2H4} + SPY_{CH3COOH} + SPY_{C2H5OH} + SPY_{n\text{-}PrOH}$, SPY of C_{2+} liquid products are listed as follow:

Catalyst	j (mA cm^{-2})	pH	Product			
			HCOOH (%)	CH_3COOH (%)	C_2H_5OH (%)	n-PrOH (%)
$Cu_{0.9}Zn_{0.1}$	400	4	FE: 2.0<br>SPY: 2.1	FE: 1.7<br>SPY: 0.9	FE: 13.4<br>SPY: 3.9	FE: 1.7<br>SPY: 0.9

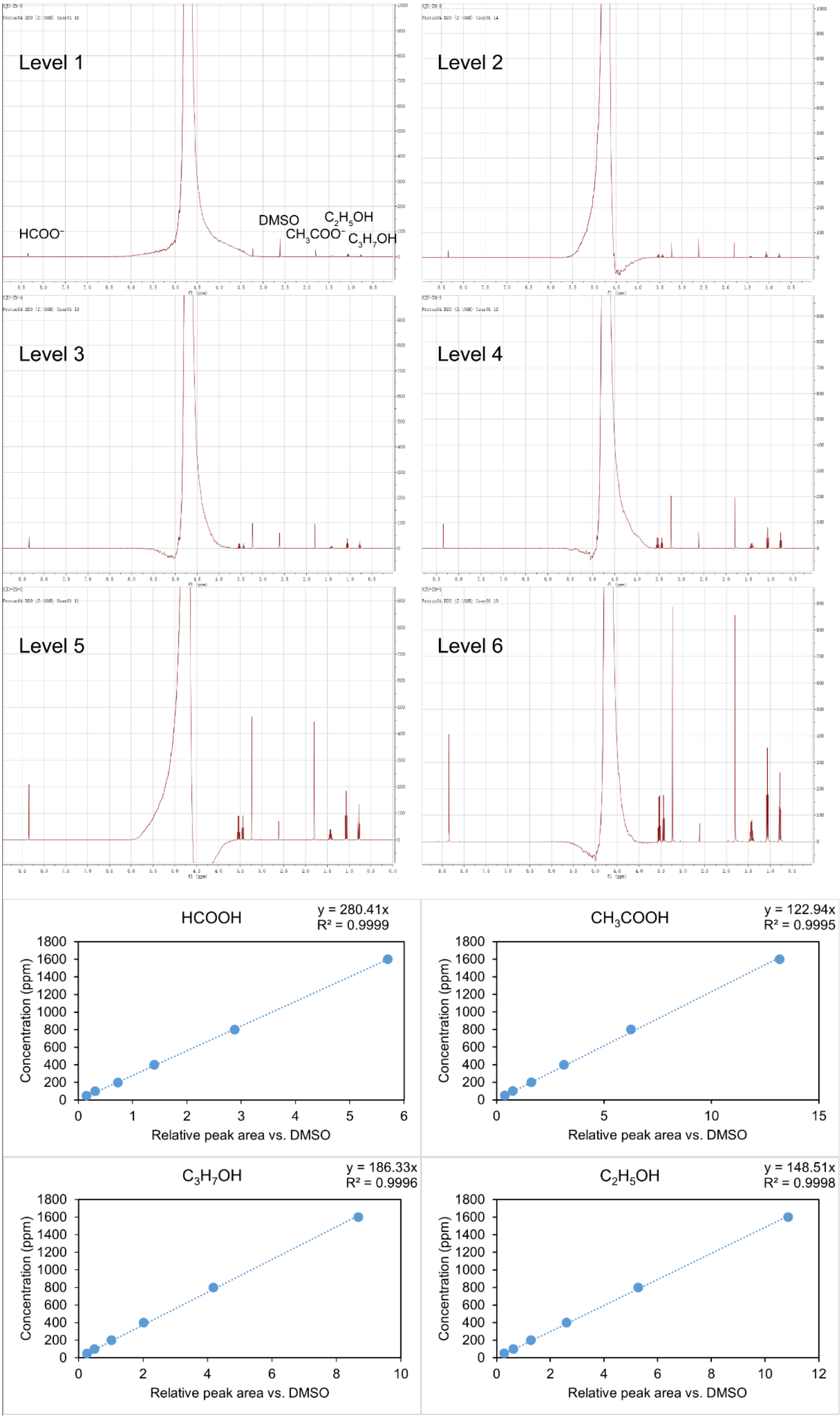

Supplementary Fig. 46 | The NMR calibration curve with all liquid products.

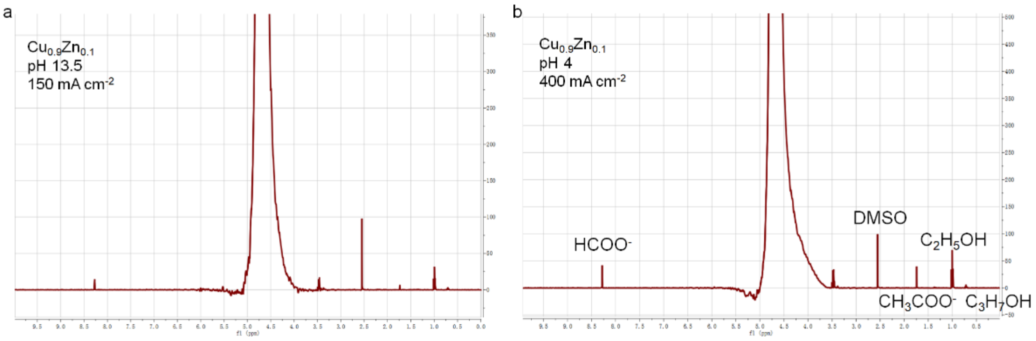

Supplementary Fig. 47 | The NMR data of (a) $Cu_{0.9}Zn_{0.1}$ (fabricated by co-sputtering and wet chemical etching) at pH 4 electrolyte on 400 mA cm^{-2} current density at a flow rate of 12 sccm, and (b) $Cu_{0.9}Zn_{0.1}$ (fabricated by co-sputtering and wet chemical etching) at pH 13.5 electrolyte on 150 mA cm^{-2} current density at a flow rate of with 15 sccm.

Supplementary Table 4 | Calculated catalytic performance based on the raw data acquired by NMR is listed as follows:

Catalyst	j (mA cm^{-2})	pH	Product			
			HCOOH (%)	CH_3COOH (%)	C_2H_5OH (%)	n-PrOH (%)
$Cu_{0.9}Zn_{0.1}$	400	4	FE: 2.0	FE: 1.7	FE: 13.4	FE: 1.7
$Cu_{0.9}Zn_{0.1}$	150	13.5	FE: 2.0	FE: 1.2	FE: 15.4	FE: 2.0

Supplementary Table 5 | Elementary steps for CO_2R to C_{2+}.

No.	Elementary steps
R1	$CO_2 + H_2O + e^- + * \rightarrow COOH* + OH^-$
R2	$COOH* + e^- \rightarrow CO* + OH^-$
R3	$CO* + H_2O + e^- \rightarrow CHO* + OH^-$
R4	$CO* + H_2O + e^- \rightarrow COH* + OH^-$
R5	$2CO* \rightarrow OCCO* + *$
R6	$CO* + CHO* \rightarrow OCCHO* + *$
R7	$CO* + COH* \rightarrow OCCOH* + *$
R8	$OCCO* + H_2O + e^- \rightarrow OCCHO* + OH^-$
R9	$OCCO* + H_2O + e^- \rightarrow OCCOH* + OH^-$
R10	$OCCHO* + 5H_2O + 7e^- \rightarrow C_2H_4 + 7OH^- + *$
R11	$OCCOH* + 5H_2O + 7e^- \rightarrow C_2H_4 + 7OH^- + *$

After C–C bond formation, subsequent protonation is relatively facile, especially under cathodic potentials, and thus was not considered in our enumerated major C–C coupling mechanisms.

Supplementary Table 6 | All possible pathways obtained by considering the elementary steps in Supplementary Table 5.

Path I	Path II
$CO_2 + H_2O + e^- + * \rightarrow COOH* + OH^-$	$CO_2 + H_2O + e^- + * \rightarrow COOH* + OH^-$
$COOH* + e^- \rightarrow CO* + OH^-$	$COOH* + e^- \rightarrow CO* + OH^-$
$CO* + H_2O + e^- \rightarrow CHO* + OH^-$	$CO* + H_2O + e^- \rightarrow COH* + OH^-$
$CO* + CHO* \rightarrow OCCHO* + *$	$CO* + COH* \rightarrow OCCOH* + *$
$OCCHO* + 5H_2O + 7e^- \rightarrow C_2H_4 + 7OH^- + *$	$OCCOH* + 5H_2O + 7e^- \rightarrow C_2H_4 + 7OH^- + *$

Path III	Path IV
$CO_2 + H_2O + e^- + * \rightarrow COOH* + OH^-$	$CO_2 + H_2O + e^- + * \rightarrow COOH* + OH^-$
$COOH* + e^- \rightarrow CO* + OH^-$	$COOH* + e^- \rightarrow CO* + OH^-$
$2CO* \rightarrow OCCO* + *$	$2CO* \rightarrow OCCO* + *$
$OCCO* + H_2O + e^- \rightarrow OCCHO* + OH^-$	$OCCO* + H_2O + e^- \rightarrow OCCOH* + OH^-$
$OCCHO* + 5H_2O + 7e^- \rightarrow C_2H_4 + 7OH^- + *$	$OCCOH* + 5H_2O + 7e^- \rightarrow C_2H_4 + 7OH^- + *$

Path V	Path VI
$CO_2 + H_2O + e^- + * \rightarrow COOH* + OH^-$	$CO_2 + H_2O + e^- + * \rightarrow COOH* + OH^-$
$COOH* + e^- \rightarrow CO* + OH^-$	$COOH* + e^- \rightarrow CO* + OH^-$
$CO* + H_2O + e^- \rightarrow CHO* + OH^-$	$CO* + H_2O + e^- \rightarrow COH* + OH^-$
$CO* + CHO* \rightarrow OCCHO* + *$	$CO* + COH* \rightarrow OCCOH* + *$
$OCCHO* + OH^- \rightarrow OCCO* + H_2O + e^-$	$OCCOH* + OH^- \rightarrow OCCO* + H_2O + e^-$
$OCCO* + H_2O + e^- \rightarrow OCCOH* + OH^-$	$OCCO* + H_2O + e^- \rightarrow OCCHO* + OH^-$
$OCCOH* + 5H_2O + 7e^- \rightarrow C_2H_4 + 7OH^- + *$	$OCCHO* + 5H_2O + 7e^- \rightarrow C_2H_4 + 7OH^- + *$

Supplementary Table 7 | Free energy corrections for adsorbates and gas molecules.

	Free energy correction (eV)
COOH*	0.49
CO*	0.11
CHO*	0.31
COH*	0.39
OCCO*	0.25
OCCHO*	0.47
OCCOH*	0.59
CO(g)	−0.39
H_2O(g)	−0.01
H_2(g)	−0.04
CO_2(g)	−0.27
C_2H_4(g)	0.82

Supplementary Table 8 | Solvent effects for adsorbates.

Solvent effect	Implicit model (eV)	Explicit model (eV)
COOH*	−0.21	−0.27
CO*	−0.04	−0.04
CHO*	−0.09	−0.09
COH*	−0.24	−0.20
OCCO*	−0.25	−0.20
OCCHO*	−0.15	−0.14
OCCOH*	−0.23	−0.26

Supplementary Table 9 | Cu and Zn atomic concentrations in the Cu_xZn_{1-x} (x = 0.95, 0.9, 0.85, 0.8, 0.7) model catalysts (fabricated by wet chemical etching of the commercial $Cu_{0.6}Zn_{0.4}$ powder, Sigma-Aldrich, Product No.: 593583-5G, <150 nm), as determined by SEM-EDS, XPS, and ICP-AES.

Catalyst	Atomic Concentration (%)					
	SEM-EDS		XPS		ICP-AES	
	Cu	Zn	Cu	Zn	Cu	Zn
Commercial $Cu_{0.95}Zn_{0.05}$	93.5	6.5	94.3	5.7	94.5	5.5
Commercial $Cu_{0.9}Zn_{0.1}$	90.5	9.5	89.5	10.5	90.8	9.2
Commercial $Cu_{0.85}Zn_{0.15}$	85.2	14.8	85.3	14.7	86.1	13.9
Commercial $Cu_{0.8}Zn_{0.2}$	80.9	19.1	79.8	20.2	80.1	19.9
Commercial $Cu_{0.7}Zn_{0.3}$	72.6	27.4	70.2	29.8	71.5	28.5

Supplementary Table 10 | FEs of all products obtained by CO_2R with the Cu_xZn_{1-x} (x = 0.95, 0.9, 0.85, 0.8, 0.7, 0.6) model catalysts (fabricated by wet chemical etching of the commercial $Cu_{0.6}Zn_{0.4}$ powder, Sigma-Aldrich, Product No.: 593583-5G, <150 nm) at 150 mA cm^{-2}. Errors represent the standard deviation based on three independent measurements.

Catalyst	j (mA cm^{-2})	FE (%)									
		Hydrogen	CO	Methane	formate	ethlyene	acetate	ethanol	n-propanol	C_{2+}	Total
Cu	150	32.8±1.6	4.6±0.5	0.6±0.2	5.1±0.8	36.6±1.3	1.4±0.1	15.3±0.7	1.6±0.2	54.9±1.3	98.0±1.9
Commercial $Cu_{0.95}Zn_{0.05}$	150	27.9±1.2	2.3±0.3	1.8±0.4	2.9±0.2	43.3±2.2	2.3±0.3	15.5±1.2	1.8±0.3	62.9±2.2	97.8±2.0
Commercial $Cu_{0.9}Zn_{0.1}$	150	24.9±0.8	2.4±0.2	1.7±0.4	2.1±0.6	48.4±2.8	1.7±0.2	14.5±1.0	2.0±0.2	66.6±2.7	97.7±2.4
Commercial $Cu_{0.85}Zn_{0.15}$	150	28.7±1.4	3.0±0.6	1.8±0.6	2.5±0.2	44.7±0.7	2.1±0.4	14.2±0.9	1.5±0.1	62.5±2.4	98.5±1.4
Commercial $Cu_{0.8}Zn_{0.2}$	150	29.3±2.5	3.7±0.4	0.5±0.2	3.9±0.4	39.6±1.6	1.7±0.1	16.9±0.5	2.5±0.1	60.7±1.8	98.1±2.9
Commercial $Cu_{0.7}Zn_{0.3}$	150	34.5±2.7	4.1±0.5	0.4±0.1	5.1±0.3	32.5±1.4	0.2±0.1	18.7±1.5	2.4±0.3	53.8±2.5	97.9±2.5
Commercial $Cu_{0.6}Zn_{0.4}$	150	44.6±2.2	7.2±0.4	0.6±0.1	6.8±0.4	24.6±1.2	1.0±0.3	13.4±1.5	1.3±0.2	40.3±0.8	99.5±1.0

Supplementary Table 11 | Cu and Zn atomic concentrations in the nanoporous Cu_yZn_{1-y} (y = 0.95, 0.9, 0.85, 0.8) catalysts (fabricated by co-sputtering and wet chemical etching), as determined by SEM-EDS, XPS, and ICP-AES.

Catalyst	Atomic Concentration (%)					
	SEM-EDS		XPS		ICP-AES	
	Cu	Zn	Cu	Zn	Cu	Zn
$Cu_{0.95}Zn_{0.05}$	95.5	4.5	95.2	4.8	95.2	4.8
$Cu_{0.9}Zn_{0.1}$	90.4	9.6	91.3	8.7	91.6	8.4
$Cu_{0.85}Zn_{0.15}$	85.1	14.9	85.7	14.3	84.1	15.9
$Cu_{0.8}Zn_{0.2}$	78.4	21.6	79.2	20.8	81.3	18.7

Supplementary Table 12 | FEs of all products obtained by CO_2R with the nanoporous Cu_yZn_{1-y} (y = 0.95, 0.9, 0.85, 0.8) and Cu catalysts (fabricated by co-sputtering and wet chemical etching) at various applied current densities in pH 13.5 electrolyte. Errors represent the standard deviation based on three independent measurements.

Catalyst	j (mA cm^{-2})	FE (%)									
		hydrogen	CO	methane	formate	Ethlyene	Acetate	Ethanol	n-propanol	C_{2+}	Total
	100	17.8±0.6	3.3±0.4	2.4±0.4	6.2±0.6	44.5±1.2	2.9±0.2	18.5±0.9	3.2±0.2	69.1±0.9	98.8±2.3
	150	14.3±1.3	3.2±0.3	1.5±0.2	5.2±0.8	49.3±1.7	2.7±0.2	20.8±0.4	2.5±0.4	75.3±0.6	99.5±1.5
Cu	200	16.7±1.2	3.4±0.2	2.3±0.2	2.5±0.5	46.3±0.6	3.8±0.3	21.0±0.4	2.2±0.1	73.3±1.2	98.2±2.5
	250	21.2±0.6	2.5±0.4	3.2±0.3	3.8±0.5	40.0±1.2	4.8±0.2	21.3±1.2	4.0±0.2	70.1±1.8	100.8±0.8
	300	27.5±0.8	3.8±0.3	2.4±0.2	3.6±0.3	38.3±0.6	2.8±0.2	20.7±0.9	1.8±0.4	63.6±1.2	100.9±0.9
	100	14.8±1.3	3.1±0.3	0.8±0.2	4.2±0.6	53.2±0.8	2.3±0.3	17.6±1.3	3.1±0.6	76.2±1.2	99.1±1.3
	150	8.8±0.3	2.2±0.2	1.1±0.3	2.0±0.2	67.4±2.1	1.4±0.2	16.8±2.3	2.1±0.4	87.7±1.4	101.8±1.9
$Cu_{0.95}Zn_{0.05}$	200	16.5±1.5	1.8±0.4	0.9±0.2	1.7±0.3	54.2±1.6	3.5±0.1	19.5±1.6	2.9±0.3	80.1±1.0	101.0±0.9
	250	19.0±0.8	2.8±0.3	0.8±0.2	2.7±0.8	50.1±0.6	2.9±0.3	18.9±0.6	2.7±0.1	74.6±0.7	99.9±1.4
	300	23.2±1.3	3.2±0.1	1.2±0.3	4.2±0.5	46.7±1.2	2.1±0.2	17.3±0.9	1.4±0.6	67.5±1.0	99.3±2.2
	100	12.6±1.2	2.6±0.2	0.2±0.1	3.8±1.0	59.1±1.9	1.5±0.4	16.3±0.6	3.5±0.4	80.4±1.0	99.6±2.2
	**150**	**7.3±0.4**	**1.3±0.1**	**0.5±0.2**	**2.0±0.2**	**73.2±1.8**	**1.2±0.2**	**15.4±1.8**	**2.0±0.4**	**91.8±1.9**	**102.9±1.7**
$Cu_{0.9}Zn_{0.1}$	200	15.9±0.2	2.3±0.2	0.8±0.1	2.1±0.5	60.7±1.2	1.6±0.1	17.4±1.2	2.1±0.2	81.8±1.6	102.9±0.5
	250	18.3±1.3	3.5±0.5	0.3±0.1	2.9±0.5	55.2±0.8	1.6±0.4	17.8±0.7	2.2±0.4	76.8±0.9	101.8±0.9
	300	21.2±0.6	3.6±0.2	0.5±0.2	3.8±0.3	49.2±1.3	1.4±0.4	16.5±0.7	2.3±0.4	69.4±1.5	98.5±1.6
	100	14.5±0.3	4.2±0.3	1.5±0.1	4.9±0.6	49.4±2.9	2.5±0.1	18.9±1.5	2.7±0.3	73.5±0.6	98.6±1.2
	150	12.2±0.7	2.7±0.2	1.3±0.4	2.9±0.3	62.4±1.3	1.8±0.4	17.8±1.2	3.1±0.3	85.1±0.4	104.2±1.2
$Cu_{0.85}Zn_{0.15}$	200	16.2±0.6	2.9±0.3	1.9±0.2	2.6±0.3	50.2±2.0	2.9±0.2	20.1±0.8	2.6±0.3	75.8±0.8	99.4±1.8
	250	19.9±1.0	2.5±0.1	1.9±0.2	3.6±0.7	46.6±1.3	3.3±0.2	19.4±1.3	2.9±0.4	72.2±1.3	100.1±2.0
	300	25.9±1.5	3.8±0.4	1.9±0.1	3.2±0.5	44.8±0.8	2.0±0.5	17.2±0.6	2.2±0.1	66.2±0.5	101.0±0.8
	100	16.7±0.6	4.8±0.2	2.9±0.2	3.7±0.6	46.3±1.6	2.6±0.5	18.7±1.2	3.6±0.4	71.2±0.8	99.3±1.1
	150	13.2±1.1	4.2±0.3	1.4±0.3	3.0±0.3	57.3±2.5	1.7±0.3	19.7±1.9	2.2±0.4	80.9±0.7	102.7±1.4
$Cu_{0.8}Zn_{0.2}$	200	17.6±0.9	3.6±0.2	2.3±0.1	1.8±0.4	47.8±0.5	2.9±0.3	19.3±0.6	3.2±0.2	73.2±0.8	98.5±2.5
	250	20.8±0.7	3.2±0.3	2.5±0.3	3.0±0.4	44.4±1.0	3.4±0.1	20.2±0.9	3.2±0.4	71.2±1.0	100.7±1.0
	300	27.2±0.9	3.4±0.3	2.8±0.3	3.2±0.4	41.9±0.6	1.8±0.2	18.9±1.2	1.6±0.2	64.2±0.8	100.8±0.8

Supplementary Table 13 | FEs of all products obtained by CO_2R with the nanoporous $Cu_{0.9}Zn_{0.1}$ catalyst (fabricated by co-sputtering and wet chemical etching) at various applied current densities in pH 1, 4, 7, and 13.5 electrolytes. Errors represent the standard deviation based on three independent measurements.

pH	*j* (mA cm^{-2})	FE (%)									
		hydrogen	CO	methane	formate	Ethlyene	Acetate	Ethanol	n-propanol	C_{2+}	Total
1	200	60.3±1.8	5.1±0.2	1.1±0.3	1.5±0.7	19.0±1.2	0.4±0.1	6.9±0.3	0.2±0.1	26.5±0.8	94.8±2.3
	300	59.0±2.0	4.9±0.3	1.5±0.2	1.5±0.3	20.5±1.7	0.3±0.1	8.4±0.5	0.6±0.2	29.8±0.7	96.7±1.9
	400	58.0±2.2	4.2±0.3	0.8±0.1	1.6±0.4	21.4±0.6	1.5±0.2	6.6±0.7	0.7±0.1	30.2±1.3	94.8±1.5
	**500**	**54.5±2.6**	**6.4±0.4**	**1.1±0.2**	**1.2±0.5**	**23.5±1.2**	**0.6±0.1**	**8.3±1.1**	**0.5±0.1**	**32.9±1.2**	**96.1±1.8**
	600	59.3±2.4	5.9±0.2	1.7±0.1	2.3±0.2	17.4±0.6	0.6±0.1	5.2±0.8	0.6±0.1	23.8±1.0	93.0±2.5
4	200	39.0±1.3	4.2±0.3	1.5±0.3	5.3±0.4	34.0±0.8	0.9±0.1	7.7±0.2	3.4±0.6	46±1.8	96.0±2.6
	300	29.0±1.2	2.9±0.2	1.0±0.1	4.3±0.3	43.0±0.6	1.2±0.2	13.3±0.5	2.2±0.1	59.7±1.2	96.9±1.4
	**400**	**26.5±1.5**	**1.5±0.4**	**0.1±0.1**	**2.0±0.4**	**52.1±1.2**	**1.7±0.2**	**13.4±0.4**	**1.7±0.2**	**68.9±2.0**	**99.0±1.2**
	500	38.7±0.8	1.9±0.3	1.0±0.2	1.5±0.3	40.5±1.1	1.5±0.3	12.5±1.2	0.4±0.1	54.9±0.5	98.0±1.3
	600	52.2±1.8	2.9±0.1	0.1±0.1	0.2±0.1	29.2±0.8	1.2±0.2	9.8±0.2	0.6±0.1	39.2±0.8	94.6±0.8
7	200	19.3±1.1	2.3±0.2	0.3±0.1	3.0±0.3	58.2±1.6	0.4±0.1	11.5±1.4	3.3±0.5	73.4±1.3	98.3±0.7
	**300**	**15.8±2.2**	**1.5±0.4**	**0.8±0.3**	**1.5±0.3**	**64.1±2.4**	**1.4±0.3**	**14.5±1.6**	**1.5±0.3**	**81.5±2.0**	**101.1±1.5**
	400	24.1±1.2	1.7±0.3	0.4±0.1	1.3±0.2	58.0±1.2	1.5±0.4	12.1±0.5	2.6±0.2	74.2±0.8	101.7±0.6
	500	34.6±1.2	1.9±0.3	0.8±0.2	0.6±0.1	45.9±1.0	2.7±0.4	13.7±0.6	2.4±0.3	64.7±1.7	102.6±1.2
	600	45.7±1.5	1.7±0.2	0.2±0.1	0.7±0.1	36.4±1.4	2.1±0.3	11.3±0.4	2.6±0.1	52.4±1.4	100.7±0.9
13.5	100	12.6±1.2	2.6±0.2	0.2±0.1	3.8±1.0	59.1±1.9	1.5±0.4	16.3±0.6	3.5±0.4	80.4±1.0	99.6±2.2
	**150**	**7.3±0.4**	**1.3±0.1**	**0.5±0.2**	**2.0±0.2**	**73.2±1.8**	**1.2±0.2**	**15.4±1.8**	**2.0±0.4**	**91.8±1.9**	**102.9±1.7**
	200	15.9±0.2	2.3±0.2	0.8±0.1	2.1±0.5	60.7±1.2	1.6±0.1	17.4±1.2	2.1±0.2	81.8±1.6	102.9±0.5
	300	21.2±0.6	3.6±0.2	0.5±0.2	3.8±0.3	49.2±1.3	1.4±0.4	16.5±0.7	2.3±0.4	69.4±1.5	98.5±1.6

Supplementary Table 14 | pH of catholyte and anolyte before and after CO_2 reduction.

Electrolyte	Catholyte		Anolyte	
pH 1	0 h	1.05	0 h	0.99
	1 h	1.06	1 h	0.99
	2 h	1.08	2 h	1.03
	3 h	1.14	3 h	1.05
pH 4	0 h	4.09	0 h	0.96
	1 h	3.99	1 h	0.98
	2 h	3.95	2 h	1.03
	3 h	3.84	3 h	1.09
pH 7	0 h	7.12	0 h	7.15
	1 h	7.15	1 h	7.19
	2 h	7.21	2 h	7.28
	3 h	7.26	3 h	7.43
pH 13.5	0 h	13.52	0 h	13.55
	1 h	13.51	1 h	13.52
	2 h	13.51	2 h	13.51
	3 h	13.51	3 h	13.49

Supplementary Table 15 | pH of catholyte and anolyte of 3 M KOTf and 0.01 M KH_2PO_4 at pH 4 before and after CO_2 reduction.

Electrolyte	Catholyte		Anolyte	
3 M KOTf + 0.01 M KH_2PO_4 (pH 4)	0 h	3.96	0 h	3.96
	1 h	3.98	1 h	3.99
	2 h	3.98	2 h	4.03
	3 h	4.01	3 h	4.03

Supplementary Table 16 | Single-pass yields of $Cu_{0.9}Zn_{0.1}$ (fabricated by co-sputtering and wet chemical etching) at various CO_2 flow rates in pH 13.5 during CO_2R in serpentine-channel reaction areas with 4.5, 9, 13.5 cm^2. Errors represent the standard deviation based on three independent measurements.

Area (cm^2)	CO_2 flow rate ($mL\ min^{-1}$)	j ($mA\ cm^{-2}$)	Single-pass yield (%)							
			CO	methane	formate	Ethlyene	Acetate	Ethanol	n-propanol	Total C_{2+}
4.5	5	150	2.6±0.2	0.3±0.1	2.4±0.4	11.7±0.9	0.5±0.1	3.1±0.3	0.5±0.1	15.8±0.7
	7	150	1.4±0.4	0.1±0.1	0.8±0.2	13.5±1.3	0.5±0.1	2.6±0.8	0.6±0.2	17.3±0.8
	10	150	1.1±0.1	0.1±0.1	0.7±0.2	10.1±0.6	0.2±0.1	1.7±0.2	0.5±0.1	12.5±0.3
9	10	150	1.5±0.4	0.5±0.1	1.1±0.2	14.8±1.3	0.5±0.2	3.9±0.5	0.8±0.2	20.0±1.2
	12	150	1.3±0.2	0.2±0.1	0.8±0.2	17.2±1.8	0.5±0.1	3.1±0.8	0.6±0.1	21.4±1.4
	15	150	1.0±0.2	0.1±0.1	0.7±0.1	15.2±1.6	0.3±0.1	2.4±0.5	0.4±0.1	18.3±0.6
13.5	12	150	2.6±0.2	1.0±0.1	2.0±0.2	16.5±1.2	0.7±0.2	4.4±0.6	0.8±0.2	22.4±1.3
	15	150	1.8±0.1	0.8±0.2	1.1±0.1	19.0±0.5	1.0±0.2	3.9±0.8	1.0±0.1	24.9±1.5
	17	150	1.8±0.2	0.9±0.1	0.7±0.1	15.2±1.5	0.7±0.1	3.3±0.4	0.7±0.2	19.9±0.7

Supplementary Table 17 | Single-pass C_{2+} yield of $Cu_{0.9}Zn_{0.1}$ (fabricated by co-sputtering and wet chemical etching) at different CO_2 flow rates and current densities in pH 1, 4, 7 and 13.5 electrolytes in a 13.5 cm^2 cell. Errors represent the standard deviation based on three independent measurements.

pH	Area (cm^2)	j ($mA\ cm^{-2}$)	CO_2 flow rate ($mL\ min^{-1}$)	Single-pass C_{2+} yield (%)				
				Ethlyene	Acetate	Ethanol	n-propanol	Total C_{2+}
1	13.5	500	10	10.4±1.4	0.4±0.1	1.5±0.3	0.2±0.1	12.5±0.9
			12	13.8±0.9	0.6±0.1	1.9±0.4	0.3±0.1	16.6±0.5
			15	8.1±1.6	0.2±0.1	1.5±0.5	0.1±0.1	9.9±1.1
4	13.5	400	10	20.3±0.9	1.0±0.3	3.7±0.8	0.9±0.2	25.9±0.6
			12	24.5±1.5	0.9±0.1	4.3±0.4	1.1±0.3	30.8±1.5
			15	17.5±0.7	0.9±0.4	2.5±0.2	0.7±0.1	21.6±0.4
7	13.5	300	10	18.5±1.3	0.8±0.2	2.4±0.5	0.7±0.1	22.4±0.8
			12	21.6±1.2	1.5±0.5	2.9±0.8	0.7±0.2	26.7±0.9
			15	15.7±0.9	0.7±0.2	1.5±0.6	0.3±0.1	18.3±0.5
13.5	13.5	150	12	16.5±1.2	0.7±0.2	4.4±0.6	0.8±0.2	22.4±1.3
			15	19.0±0.5	1.0±0.2	3.9±0.8	1.0±0.1	24.9±1.5
			17	15.2±1.5	0.7±0.1	3.3±0.4	0.7±0.2	19.9±0.7

Supplementary Table 18 | CO_2 utilization efficiency in different pH solutions (ratio of CO_2 undissolved into the electrolytes to total CO_2 consumption).

	fCO_{2inlet} (ml/min)	$fCO_{2outlet}$ (ml/min)	CO_2 utilization efficiency (%)
pH 1	12	11.70	97.5±1.5
pH 4	12	10.98	91.5±1.5

The formula for CO_2 utilization efficiency for multiple uses is as follows:

$$\text{In the case of no electricity, } U_{CO_2} = \frac{f_{CO_2outlet}}{f_{CO_2inlet}} \times 100\%, \quad (1)$$

Where $f_{CO_2outlet}$ is the flow rate of the CO_2 gas at the outlet of the gas chamber, f_{CO_2inlet} is the flow rate of CO_2 gas at the inlet of the gas chamber. The above CO_2 utilization efficiency calculation included the residual CO_2 for multiple uses.

Supplementary Table 19 | Carbon balance and CO_2 utilization efficiency in pH 4 electrolyte.

j (mA cm^{-2})	$f_{CO_2 inlet}$ (ml/min)	$f_{CO_2 outlet}$ (ml/min)	$f_{CO_2 dissolved}$ (ml/min)	$f_{CO_2\ to\ gas}$ (ml/min)	$f_{CO_2\ to\ liquid}$ (ml/min)	$f_{CO_2\ residue}$ (ml/min)	CO_2 utilization efficiency (%)
400	12.0	12.04	1.02	4.10	0.68	4.12	82.41

We calculated CO_2 utilization efficiency for the single pass in which the residual CO_2 is considered as waste that can not be recycled.[S18] The formulas are as follow:

$$f_{CO_2 outlet} = f_{CO_2\ residue} + f_{CO} + f_{CH_4} + f_{C_2H_4} + f_{H_2}, \quad (2)$$

$$f_{CO_2 inlet} = f_{CO_2 residue} + f_{CO_2 conversion} + f_{CO_2 dissolved}, \quad (3)$$

$$f_{CO_2 conversion} = f_{CO_2\ to\ gas} + f_{CO_2\ to\ liquid}, \quad (4)$$

Where $f_{CO_2\ residue}$ is the unreacted CO_2 flow rate in the gas outlet during CO_2R. $f_{CO_2 conversion}$ is the consumed CO_2 flowrate which is electrochemically converted into all products. $f_{CO_2 dissolved}$ is the consumed CO_2 flow rate via the reaction with OH^-. $f_{CO_2\ to\ gas}$ is the consumed CO_2 flowrate which is electrochemically converted into all gas products (carbon monoxide, methane, ethylene). $f_{CO_2\ to\ liquid}$ is the consumed CO_2 flow rate for electrocatalytic reduction to all liquid products (formate, acetate, ethanol, n-propanol).

Supplementary Table 20 | Comparison of electrochemical CO_2-to-C_{2+} performance for the CuZn catalysts in this work and previously reported systems.

Catalyst	**C_{2+} yield (%)**	**CO_2 utilization efficiency (%)**	C_{2+} Faradaic efficiency (%)	Current density (mA cm^{-2})	Full-cell EE (%)	Stability (h)	Cathodic electrolyte	Ref.
$Cu_{0.9}Zn_{0.1}$	**31±2**	**91±1**	69±2	400	/	>35	3 M KCl (pH was adjusted to 4 using H_2SO_4)	This work
	**27±2**	**82±2**	81±2	300	/	>70	3 M KCl (pH was adjusted to 7 using KOH)	
	**24±1**	**65±1**	91±2	150	31±2	150	0.75 M KOH (pH was 13.5)	
CAL-modified Cu	/	77	40	1200	/	12	1 M H_3PO_4 + 2 M KCl	[s1]
Boron doped Cu	/	/	79	70	/	40	0.1 M KCl	[s2]
Molecular modified Cu	/	/	75	120	20	190	0.1 M $KHCO_3$	[s3]
Cu nanocubes (100) facets	/	/	60	68	/	/	0.25 M $KHCO_3$	[s4]
Electro-deposited Cu	/	/	75	150	27	30	2 M KCl	[s5]
FeTPP[Cl]/Cu	/	/	73	110	13	12	0.1 M $KHCO_3$	[s6]
Cu-Al	<5	/	85	400	/	50	1 M KOH	[s7]
Abrupt Cu Interface	<5	/	83	75-100	34	150	7 M KOH	[s8]
Fluorine modified Cu	16.5	/	80	1600	/	/	0.75 M KOH	[s9]
	<5	/	84	800	37	/	2.5 M KOH	
Porous Cu	/	/	55	200	/	2	1 M KOH	[s10]
Multihollow Cu_2O	/	/	75	342	/	3	2 M KOH	[s11]
Cu-CIBH	/	/	70	250	20	60	7 M KOH	[s12]

"/" means not reported.

Supplementary Table 21 | Comparison of CO_2-to-C_{2+} single-pass yields for the CuZn catalysts in this work with previous electrochemical and thermal catalytic systems.

Catalyst	CO_2 flow rate (mL min^{-1})	CO_2 conversion (%)	C_{2-4} selectivity (%)	C_{2-4} yield (%)	Ref.
**$Cu_{0.9}Zn_{0.1}$ (pH 4)**	**12**	**33±2**	**91±2**	**31±2**	**This work**
**$Cu_{0.9}Zn_{0.1}$ (pH 7)**	**12**	**28±1**	**93±1**	**27±2**	
**$Cu_{0.9}Zn_{0.1}$ (pH 13.5)**	**15**	**26±1**	**94±2**	**24±1**	
Abrupt Cu Interface	50	5	80	4	[s8]
Fluorine modified Cu	20	19	86	16.5	[s9]
Fe–Co/K/Al_2O_3	12	31	69	21	[s13]
K-Fe-Co/ZrO_2	15	42	52	22	[s14]
Cu–Zn–Al/modified-HB	12	27	45	13	[s15]
K-Fe15	5	45	47	21	[s16]
K-Fe/MOF	5	40	34	14	[s17]

Supplementary Table 22 | Comparison of electrochemical performance for the CuZn catalysts in this work with the previous reports.

Catalysts	Cu:Zn ratios	Device	Electrolyte	j (mA cm^{-2})	Major product, FE (%)	Stability (h)	Ref.
**Cu-ZnO**	**7:3**	H-Cell	0.1 M $KHCO_3$ (pH~7)	50	Methane, 70	5	*J. Am. Chem. Soc.* **141**, 19879-19887 (2019)
**Phase-separated Cu-Zn nanowires**	**1:1**	H-Cell	0.1 M $KHCO_3$ (pH~7)	5-20	CO, 86	15	*ACS Catal.* **12**, 2741-2748 (2022)
**Phase-segregated CuZn**	**4:1**	H-Cell	0.1 M $KHCO_3$ (pH~7)	25	Ethanol, 29<br>C_{2+}, 51.4	5	*ACS Catal.* **6**, 8239-8247 (2016)
**CuZn nanoparticles**	**4:1**	Flow-cell	1 M KOH (pH~14)	200	C_{2+}, ~25–10	5	*Angew. Chem. Int. Ed.* **61**, e202201913 (2022)
**Laser-prepared CuZn alloy**	**4:1**	H-Cell	0.1 M $KHCO_3$ (pH~7)	3	Ethylene, 33<br>C_{2+}, 34	15	*Langmuir* **34**, 13544-13549 (2018)
**Nanoporous $Cu_{0.9}Zn_{0.1}$**	**9:1**	**Flow-cell**	**pH 1-13.5**	**150**	**C_2H_4 73±2**<br>**C_{2+} 91±2**	**150**	**This work**

We suggest that the active site, proposed mechanism, and CO_2R performance are different between this work and the priorly reported CuZn work. In short, the relatively low Zn ratios of ≤10% enable abundant Cu-rich Cu-Zn site and Cu-Cu hollow site for asymmetric CO* bindings to improve C–C coupling. The other references suggested weakened CO* binding on surfaces including the Zn-rich Cu-Zn site, Cu-Zn-balanced Cu-Zn site, and Zn site surrounded by Cu. The performance is different between this work and the other publications.

Supplementary Table 23 | Cu and Zn atomic concentrations in $Cu_{0.9}Zn_{0.1}$ catalyst (fabricated by co-sputtering and wet chemical etching) before and after the 150 hours stability test, as determined by ICP-AES.

Catalyst	Atomic Concentration (%)		
	Cu	Zn	real error (%) 1 sigma
$Cu_{0.9}Zn_{0.1}$ before 150 h stability test	91.6	8.4	0.2
$Cu_{0.9}Zn_{0.1}$ after 150 h stability test	91.1	8.9	0.2

Supplementary References

S1 Huang, J. E. et al. CO_2 electrolysis to multicarbon products in strong acid. *Science* **372**, 1074-1078 (2021).

S2 Zhou, Y. et al. Dopant-induced electron localization drives CO_2 reduction to C_2 hydrocarbons. *Nat. Chem.* **10**, 974-980 (2018).

S3 Li, F. et al. Molecular tuning of CO_2-to-ethylene conversion. *Nature* **577**, 509-513 (2020).

S4 Zhang, X. et al. Selective and high current CO_2 electro-reduction to multicarbon products in near-neutral KCl electrolytes. *J. Am. Chem. Soc.* **143**, 3245-3255 (2021).

S5 Jiang, K. et al. Metal ion cycling of Cu foil for selective C–C coupling in electrochemical CO_2 reduction. *Nat. Catal.* **1**, 111-119 (2018).

S6 Li, F. et al. Cooperative CO_2-to-ethanol conversion via enriched intermediates at molecule–metal catalyst interfaces. *Nat. Catal.* **3**, 75-82 (2020).

S7 Zhong, M. et al. Accelerated discovery of CO_2 electrocatalysts using active machine learning. *Nature* **581**, 178-183 (2020).

S8 Dinh, C.-T. et al. CO_2 electroreduction to ethylene via hydroxide-mediated copper catalysis at an abrupt interface. *Science* **360**, 783-787 (2018).

S9 Ma, W. et al. Electrocatalytic reduction of CO_2 to ethylene and ethanol through hydrogen-assisted C–C coupling over fluorine-modified copper. *Nat. Catal.* **3**, 478-487 (2020).

S10 Lv, J.-J. et al. A highly porous copper electrocatalyst for carbon dioxide reduction. *Adv. Mater.* **30**, 1803111 (2018).

S11 Yang, P.-P. et al. Protecting copper oxidation state via intermediate confinement for selective CO_2 electroreduction to C_{2+} fuels. *J. Am. Chem. Soc.* **142**, 6400-6408 (2020).

S12 García de Arquer, F. P. et al. CO_2 electrolysis to multicarbon products at activities greater than 1 A cm^{-2}. *Science* **367**, 661-666 (2020).

S13 Satthawong, R., Koizumi, N., Song, C. & Prasassarakich, P. Light olefin synthesis from CO_2 hydrogenation over K-promoted Fe–Co bimetallic catalysts. *Catal. Today* **251**, 34-40 (2015).

S14 Li, W. et al. The anti-sintering catalysts: Fe–Co–Zr polymetallic fibers for CO_2 hydrogenation to C2= –C4= –rich hydrocarbons. *J. CO_2 Util.* **23**, 219-225 (2018).

S15 Fujiwara, M., Satake, T., Shiokawa, K. & Sakurai, H. CO2 hydrogenation for C2+ hydrocarbon synthesis over composite catalyst using surface modified HB zeolite. *Appl. Catal. B-Environ* **179**, 37-43 (2015).

S16 Visconti, C. G. et al. CO_2 hydrogenation to lower olefins on a high surface area K-promoted bulk Fe-catalyst. *Appl. Catal. B-Environ* **200**, 530-542 (2017).

S17 Ramirez, A., Gevers, L., Bavykina, A., Ould-Chikh, S. & Gascon, J. Metal organic framework-derived iron catalysts for the direct hydrogenation of CO_2 to short chain olefins. *ACS Catal.* **8**, 9174-9182 (2018).

S18 Ma, M. et al. Insights into the carbon balance for CO_2 electroreduction on Cu using gas diffusion electrode reactor designs. *Energy Environ. Sci.* **13**, 977-985 (2020).
